# Sodium chloride in the tumor microenvironment enhances T cell metabolic fitness and cytotoxicity

**DOI:** 10.1038/s41590-024-01918-6

**Published:** 2024-08-28

**Authors:** Dominik Soll, Chang-Feng Chu, Shan Sun, Veronika Lutz, Mahima Arunkumar, Mariam Gachechiladze, Sascha Schäuble, Maha Alissa-Alkhalaf, Trang Nguyen, Michelle-Amirah Khalil, Ignacio Garcia-Ribelles, Michael Mueller, Katrin Buder, Bernhard Michalke, Gianni Panagiotou, Kai Ziegler-Martin, Pascal Benz, Philipp Schatzlmaier, Karsten Hiller, Hannes Stockinger, Maik Luu, Kilian Schober, Carolin Moosmann, Wolfgang W. Schamel, Magdalena Huber, Christina E. Zielinski

**Affiliations:** 1 Technical University of Munich, Munich, Germany; 2 Department of Infection Immunology, Leibniz Institute for Natural Product Research and Infection Biology, Hans Knöll Institute, Jena, Germany; 3 Institute of Systems Immunology, Philipps-University Marburg, Marburg, Germany; 4 Department of Microbiome Dynamics, Institute for Natural Product Research and Infection Biology, Hans Knöll Institute, Jena, Germany; 5 Institute of Biology III, Faculty of Biology and Signalling Research Centres BIOSS and CIBSS, University of Freiburg, Freiburg, Germany; 6 Center of Chronic Immunodeficiency, University Clinics and Medical Faculty, University of Freiburg, Freiburg, Germany; 7 Spemann Graduate School of Biology and Medicine, University of Freiburg, Freiburg, Germany; 8 Department of Bioinformatics and Biochemistry, Braunschweig Integrated Centre of Systems Biology, Technical University of Braunschweig, Braunschweig, Germany; 9 Leibniz Institute for Aging, Jena, Germany; 10 Research Unit Analytical BioGeoChemistry, Helmholtz Center Munich—German Research Center for Environmental Health (GmbH), Neuherberg, Germany; 11 Jena University Hospital, Friedrich Schiller University, Jena, Germany; 12 Chair for Cellular Immunotherapy, Medizinische Klinik und Poliklinik II, Universitätsklinikum Würzburg, Würzburg, Germany; 13 Medical University of Vienna, Center for Pathophysiology, Infectiology and Immunology, Institute for Hygiene and Applied Immunology, Vienna, Austria; 14 Mikrobiologisches Institut—Klinische Mikrobiologie, Immunologie und Hygiene, Universitätsklinikum Erlangen und Friedrich-Alexander-Universität Friedrich-Alexander-Universität (FAU) Erlangen-Nürnberg, Erlangen, Germany; 15 FAU Profile Center Immunomedicine, FAU Erlangen-Nürnberg, Erlangen, Germany; 16 Institute of Microbiology, Faculty of Biological Sciences, Friedrich Schiller University Jena, Jena, Germany

**Keywords:** CD8-positive T cells, Immunological memory, Immunosurveillance, Lymphocyte activation

## Abstract

The efficacy of antitumor immunity is associated with the metabolic state of cytotoxic T cells, which is sensitive to the tumor microenvironment. Whether ionic signals affect adaptive antitumor immune responses is unclear. In the present study, we show that there is an enrichment of sodium in solid tumors from patients with breast cancer. Sodium chloride (NaCl) enhances the activation state and effector functions of human CD8^+^ T cells, which is associated with enhanced metabolic fitness. These NaCl-induced effects translate into increased tumor cell killing in vitro and in vivo. Mechanistically, NaCl-induced changes in CD8^+^ T cells are linked to sodium-induced upregulation of Na^+^/K^+^-ATPase activity, followed by membrane hyperpolarization, which magnifies the electromotive force for T cell receptor (TCR)-induced calcium influx and downstream TCR signaling. We therefore propose that NaCl is a positive regulator of acute antitumor immunity that might be modulated for ex vivo conditioning of therapeutic T cells, such as CAR T cells.

## Abstract (web summary)

In this paper and the related paper from Lugli and colleagues the authors show that high levels of NaCl inside the tumor have a beneficial effect on CD8^+^ T cells and their ability to control tumor growth as a result of enhanced T cell receptor activity.

## Main

CD8^+^ T cells with reactivity against tumors can mediate potent cytotoxic effector responses that rely on target specificity, potent effector functions and long-term maintenance. The tumor microenvironment paralyzes the antitumor immune response by limiting the infiltration of T cells, impairing T cell maintenance and suppressing effector functions^1^. Several lines of investigation have recently suggested a role for extracellular ions in modulating T cell effector responses. K^+^ ions were found to be enriched in the necrotic tumor microenvironment and to suppress TCR-driven T cell effector programs, while concomitantly promoting the stemness and thus self-renewal, expansion and multipotency of these cells^2,3^. This prompted the suggestion that K^+^ ions act as a tumor-induced ionic checkpoint influencing T cell effector functions. Elevated Na^+^ concentrations were demonstrated to strongly promote the differentiation of helper 17 T cells (T_H_17) cells under polarizing conditions and the upregulation of self-regulatory cytokines in these cells on restimulation^4–6^. Recently, Na^+^ ions were also shown to abrogate the immunosuppressive function of regulatory T cells (T_reg_ cells) through perturbation of mitochondrial respiration^7,8^. In addition, Na^+^ ions promoted T_H_2 cell responses through mTORC2 signaling with implications in the pathogenesis of allergic diseases^9^. However, the direct effect of Na^+^ ions on CD8^+^ T cells, and thus on antitumor cytotoxicity, is still unknown.

In the present study, we found increased intratumoral Na^+^ ion concentrations that left transcriptomic imprints on tumor-infiltrating immune cells. We therefore investigated how increased extracellular Na^+^ ion concentrations affected CD8^+^ T cell activation and effector functions and identified a molecular mechanism linking ionic imbalance to metabolic T cell reprogramming and tumor control.

## Results

### Sodium is increased in the tumor microenvironment

**Fig. 1:**
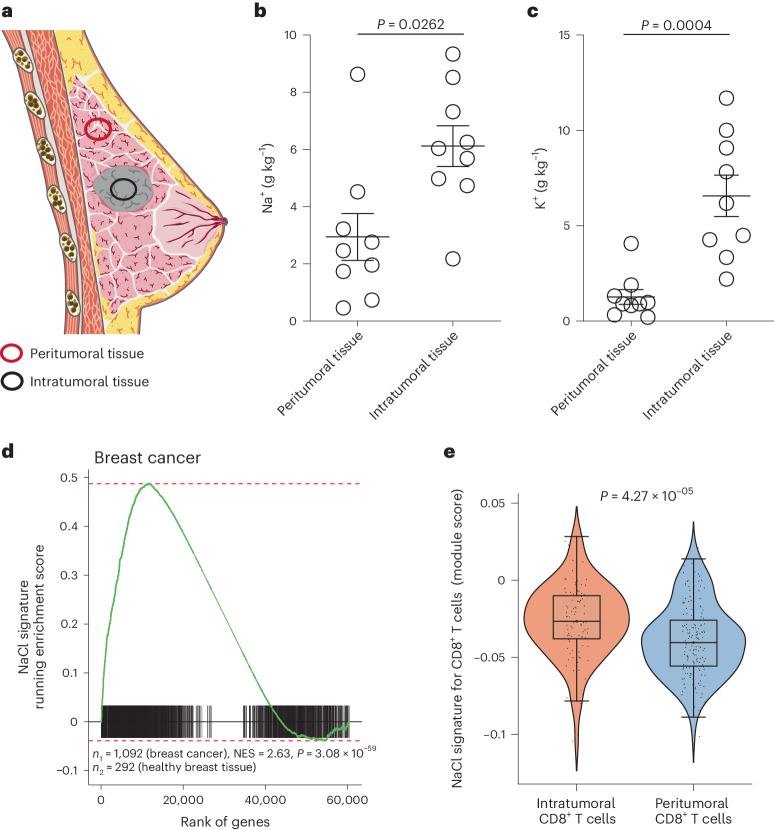
NaCl is highly enriched in solid tumors. **a**, Schematic presentation of intratumoral and peritumoral tissue biopsies from patients with breast cancer. **b**,**c**, Quantification of Na^+^ (**b**) and K^+^ (**c**) concentrations with ICP–OES in intratumoral and peritumoral biopsies from patients with breast cancer (*n* = 9; mean ± s.e.m., two-tailed, paired Student’s *t*-test). **d**, Preranked GSEA of patients with breast cancer from TCGA. Running enrichment scores and sorted positions of the 1,956 significantly upregulated genes from the NaCl signature are shown. The NaCl signature was generated by transcriptomic comparison of CD8^+^ memory T cells cultured under high versus low NaCl conditions (top 60 significantly upregulated DEGs). *P*, significance of the enrichment (one-tailed test for positive enrichment); *n*_1_ and *n*_2_, number of patients providing either solid tumor samples or healthy breast tissue samples, respectively. **e**, ScRNA-seq of intra- and peritumoral CD8^+^ T cells from patients with breast cancer (*n* = 3) (GEO accession no. GSE114727)^11^. The module score for the transcriptomic NaCl signature obtained from DEGs on direct transcriptomic comparison of CD8^+^CD45RA^−^ T cells from high compared with low NaCl conditions, as described in **d**, was tested in intra- and peritumoral CD8^+^ T cells. CD8^+^ T cells were identified using marker gene expression.

To assess the potential influence of ionic signals on the immune regulation of human CD8^+^ T cells in the tumor microenvironment, we first determined the concentrations of Na^+^ and K^+^ ions in intratumoral and peritumoral tissue of patients with breast cancer (Fig. 1a and Table 1). Fresh tissue biopsies were analyzed using inductively coupled plasma optical emission spectrometry (ICP–OES)^10^. We found significantly higher concentrations of Na^+^ ions in intratumoral breast cancer tissue than in patient-matched peritumoral tissue (Fig. 1b). In agreement with previous reports^2^, we also observed a significant accumulation of K^+^ ions in tumor tissue (Fig. 1c).

**Table 1:** Breast cancer patient clinical information

Patient no.	Sample type	Na^+^ (g kg^−1^)	K^+^ (g kg^−1^)	Sex	Tumor status	Nodal status	Metastasis	Grading	Stage according to UICC	Estrogen receptor (%)	Progesterone receptor (%)	Her2 status	Histological classification	Localization of breast tumor	Neoadjuvant therapy
1	Lesional tumor	2.18	3.34	F	pT1c, pTis (DCIS, non-high-grade with necrosis—intermediate malign)	pN0 (sn), (0/2)	MX	G2	IV	100	90	2+ (FISH negative)	Ductal	Right	No
2	Perilesional tissue	3.22	1.89												
3	Lesional tumor	4.98	4.27	F	pT1c (m), pTis	pN1a (1/13)	cM0	G2	IIA	95	70	Negative (DAKO-score 1+)	Lobular	Left	No
4	Perilesional tissue	0.735	0.337												
5	Lesional tumor	9.34	4.49	F	pT4b	pN2a (8/10)	MX	G1	IV	90		2+ (positiv).	Ductal	Left	No
6	Perilesional tissue	2.46	0.905												
7	Lesional tumor	4.74	6.18	F	pT1c	pN(sn)0 (0/2)	MX	G2, BRE-score: 6 (2 + 1 + 3)	IV	100	2	2+ (FISH negative)	Ductal	Left	No
8	Perilesional tissue	2.76	0.978												
9	Lesional tumor	8.52	2.21	F	pT1c	pN(sn)0 (0/3)	MX	G1	IV	10	100	2+ (FISH negative)	Mucinous	Left	No
10	Perilesional tissue	0.464	0.207												
11	Lesional tumor	6.54	11.7	F	pT2	pN1 (1/9)	MX	G2, BRE-score: 7 (1 + 3 + 3)	IV	100	70	Negative	Ductal	Right	No
12	Perilesional tissue														
13	Lesional tumor	6.04	9.08	F	pT2	pN0 (sn) (0/2)	MX	G2	IV	100	95	Negative (score 1+)	Ductal	Right	No
14	Perilesional tissue	1.74	0.91												
15	Lesional tumor	6.25	10	F	pT1c	pN(sn)0 (0/2)	cM0	G3, BRE-score 9	I	Negative	Negative	2+ (FISH negative)	Ductal	Left	No
16	Perilesional tissue	1.96	0.823												
17	Lesional tumor	5.69	11.7	F	pT1c(m), pTis	pN0sn (0/5)	cM0	G2 (invasive component), WHO grade II (DCIS)	I	90% positive in the invasive carcinoma, 90% in the in situ component	Positive (80%) invasive carcinoma, 20% in situ component	2+ (FISH negative)	Ductal	Left	No
18	Perilesional tissue	8.63	4.07												
19	Lesional tumor	7.32	7.83	F	pT4b	pN2a (7/7)	MX	G3, BRE-score: 8 (3 + 3 + 2)	IV	Negative	Negative	2+ (FISH negative)	Ductal	Right	No
20	Perilesional tissue	4.52	1.32												

cM0, no cancer spread; DCIS, ductal cancer in situ; F, female; MX, distant metastasis; UICC, Union for International Cancer Control.

Next, we explored whether the increased Na^+^ concentrations in the intratumoral microenvironment could influence antitumor immunity. Therefore, we investigated whether total tumor tissue, and specifically tumor-infiltrating human CD8^+^ T cells (CD8^+^ TILs), displayed a transcriptomic imprint of NaCl exposure. To this end, we first established a transcriptomic gene signature associated with NaCl exposure for CD8^+^ T cells by stimulating CD8^+^CD45RA^−^ T cells with CD3 and CD28 monoclonal antibodies (mAbs) for 5 d under low (standard 140 mM) and high (+67.5 mM) extracellular NaCl conditions in vitro. Significantly upregulated, differentially expressed genes (DEGs) after high NaCl treatment formed the gene set (NaCl signature) for further downstream analyses (Supplementary Table 1). Then, 36–1,278 patients per cancer type (14,171 samples of 9,554 patients in total) and 25 distinct tumor types from the publicly available TGCA (The Cancer Genome Atlas) and GTEx (Genotype-Tissue Expression) projects were investigated to evaluate the enrichment of the NaCl signature in tumor tissues compared with healthy tissues. Interestingly, we observed breast cancer and all other solid tumor entities (23 out of 25), except tumors in the thymus and paraganglia, displayed a significant enrichment of the NaCl signature relative to paired healthy tissues (Fig. 1d and Supplementary Fig. 1).

We next sought to determine whether the transcriptomic NaCl signature, which we found enriched in the overall tumor microenvironment, was also found in CD8^+^ TILs. We analyzed public single-cell RNA sequencing (scRNA-seq) data from matched tumoral and peritumoral tissues from patients with breast cancer^11^ (Supplementary Fig. 2a–c). In all three patients tested, we found a stronger enrichment of the NaCl signature in CD8^+^ TILs than in matched CD8^+^ T cells from the peritumoral breast tissue (Fig. 1e). These results were corroborated by using an independent NaCl signature that we established from a single-cell transcriptomic comparison of memory CD8^+^ T cells, which had been polyclonally stimulated under high and low NaCl conditions for 3 d (Supplementary Figs. 3 and 4a and Supplementary Table 2).

In essence, these data demonstrate that Na^+^ enrichment is a characteristic feature of the tumor microenvironment and that it significantly shapes the transcriptome of CD8^+^ TILs in humans.

### NaCl increases T cell activation and TCR signaling in CD8^+^ T cells

**Fig. 2:**
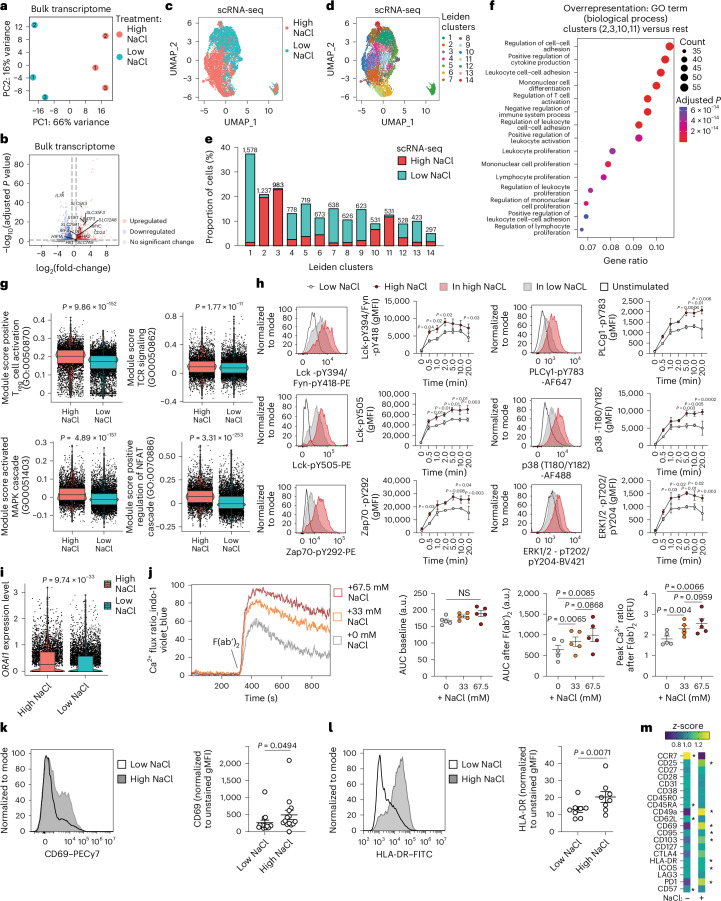
NaCl enhances the activation state of human CD8^+^ memory T cells. **a**, PCA projection after mRNA-seq of bulk human CD8^+^CD45RA^−^ T cells stimulated with CD3 and CD28 mAbs for 5 d under high and low NaCl conditions. The nos. 1–3 represent the individual blood donors. **b**, mRNA-seq analysis as in **a**. DEGs were identified using DESeq2 Wald’s test. **c**,**d**, ScRNA-seq of human CD8^+^CD45RA^−^ T cells stimulated with CD3 and CD28 mAbs for 3 d under high and low NaCl conditions.The UMAP shown is a representation visualizing the distribution of cells according to their respective treatment condition (**c**) and according to Leiden clustering (**d**). **e**, Proportion of CD8^+^CD45RA^−^ T cells from high and low NaCl conditions within the Leiden clusters after scRNA-seq as in **c**. Bray–Curtis dissimilarity testing was between high NaCl clusters (2, 3, 10, 11) and low NaCl clusters (1, 4–9, 12–14) (*P* = 1.5 × 10^−17^, Wilcoxon’s rank-sum test). **f**, GSEA showing the top 15 GO terms among all biological processes. **g**, Module scores for the indicated gene sets comparing CD8^+^CD45RA^−^ T cells from high and low NaCl conditions after scRNA-seq (Wilcoxon’s rank-sum test). **h**, Phospho-low cytometry of CD8^+^CD45RA^−^ T cells stimulated as in **a** after TCR crosslinking with anti-CD3 mAbs and anti-mouse IgG F(ab′)_2_ (*n* = 3; mean ± s.e.m., two-way ANOVA with Fisher’s least significant difference (LSD)). gMFI, geometric mean fluorescence intensity. **i**, ScRNA-seq analysis of CD8^+^CD45RA^−^ T cells stimulated as in **a** (Wilcoxon’s rank-sum test). **j**, Ca^2+^ flux measurement after expansion of human CD8^+^CD45RA^−^ T cells with CD3 and CD28 mAbs for 5 d in low and high NaCl conditions by flow cytometry. A representative plot shows the Ca^2+^ flux ratio in different NaCl conditions after anti-CD3 crosslinking F(ab′)_2_. Dot plots show the area under the curve (AUC) during baseline, after F(ab′)_2_ and the peak value of Ca^2+^ flux ratio after F(ab′)_2_. Data present the mean ± s.e.m. from individual donors (*n* = 5, one-way ANOVA with Tukey’s multiple-comparison test). a.u., arbitrary units; RFU, relative fluorescence units. **k**,**l**, Spectral flow cytometric analysis of CD8^+^CD45RA^−^ T cells stimulated as in **a** (*n* = 14 (**k**), *n* = 8 (**l**), mean ± s.e.m., two-tailed, paired Student’s *t*-test). **m**, Spectral flow cytometry. The differences in the percentages of cells positive for the shown markers were visualized by *z*-score (*n* = 4; ^*^P < 0.05. two-tailed, paired Student’s *t*-test). The asterisk is located on the treatment side (high or low NaCl) that shows significant upregulation.

To gain mechanistic insight into the effects of NaCl on CD8^+^ T cells, we next explored the specific effect of NaCl on the bulk transcriptome of human CD8^+^CD45RA^−^ T cells in vitro (Supplementary Fig. 5a, gating strategy). High NaCl conditions significantly changed the overall transcriptome of CD3- and CD28-stimulated human CD8^+^CD45RA^−^ T cells in three healthy donors as assessed by principal component analysis (PCA) (Fig. 2a). A total of 1,956 genes were significantly up- and 1,926 genes significantly downregulated, respectively (Supplementary Fig. 5b,c and Supplementary Table 1). *SGK1*, which encodes a NaCl-inducible kinase, was significantly upregulated on stimulation under high NaCl conditions in CD8^+^ memory T cells (Fig. 2b). In addition, several genes encoding members of the solute carrier group of membrane transport proteins were among the top 50 significantly upregulated DEGs (*SLC5A3*, *SLC35F3*, *SLC12A8* and *SLC29A1*), indicating enhanced uptake of various metabolites, such as sugar (*SLC5A3*) and amino acids (*SLC7A5*), and enhanced glycolytic metabolic processing (*HK1, HK2*) on exposure to high NaCl conditions (Fig. 2b and Supplementary Table 1). *MYC* and *HIF1A*, the target genes of the mammalian target of rapamycin (mTOR), which represents the central hub of mammalian metabolism^12^, were also significantly upregulated at high NaCl exposure. Notably, *BATF3*, which augments CD8^+^ T cell metabolic fitness, viability and memory development, was among the top upregulated genes^13^ (Fig. 2b). In addition, *LTA*, which encodes lymphotoxin α, a cytotoxic effector protein with implications in tumor killing^14^, was significantly upregulated by NaCl (Fig. 2b). Likewise, *IRF4* and *CD24* expression, which is crucial for the sustained expansion and effector function of cytotoxic CD8^+^ T cells, was significantly increased under high NaCl conditions^15,16^. Taken together, these data demonstrate NaCl-induced changes in the transcriptome of human CD8^+^ memory T cells, which point to an enhanced state of activation.

We then performed an scRNA-seq analysis of CD45RA^−^CD8^+^ T cells to dissect differential effects of NaCl on distinct CD8^+^ T cell types. Uniform Manifold Approximation and Projection (UMAP) analysis supported our conclusion that memory CD8^+^ T cells treated under high and low NaCl conditions clustered separately (Fig. 2c). Some 14 individual clusters (Fig. 2d,e), which we characterized in more detail by determining their respective marker genes (Supplementary Fig. 6a), were determined by Leiden clustering. Cluster 12, which clustered apart from all other clusters, was characterized by a high expression of mitochondrial genes indicating reduced viability (Supplementary Fig. 6b)^17^. The relative proportion of cells from the high NaCl treatment group was smaller than that from the low NaCl treatment group in this ‘low viability’ cluster, suggesting that NaCl exerts protective effects on cell death. To validate this conclusion, we experimentally tested the overall viability of CD8^+^ memory T cells on stimulation with a wide range of extracellular NaCl concentrations over a 5-d culture period. We found that neither viability, as determined by total cell number and cell cycles, nor frequency of dying or apoptotic cells changed in response to high NaCl conditions, as assessed by 7-aminoactinomycin D (7-AAD) and annexin V staining (Supplementary Fig. 7a–c).

Unbiased overrepresentation analysis demonstrated that activation, proliferation, differentiation and effector functions, which are known to be relevant for antitumor immunity, were among the top 15 enriched biological processes on comparison of clusters dominated by T cells from high compared with low NaCl conditions (clusters 2, 3, 10 and 11 versus the rest of the clusters) (Fig. 2f and Supplementary Fig. 4b). This finding was supported with a gene overrepresentation analysis comparing total CD8^+^ T cells from either high or low NaCl conditions (Supplementary Fig. 8). We found a significantly increased expression of the module score for T cell activation on the single-cell transcriptomic level, as well as for pathways representing proximal TCR activation and T cell activation downstream of the TCR, such as the MAPK (mitogen-activated protein kinase) and NFAT (nuclear factor of activated T cells) pathways (Fig. 2g). For functional validation, we systematically interrogated the proximal TCR signaling cascade by assessing phosphorylation of signaling proteins over time after TCR crosslinking following prestimulation of CD8^+^CD45RA^−^ T cells with CD3 and CD28 mAbs for 5 d under high and low NaCl conditions. We found significantly increased phosphorylation of Lck, ZAP70, p38 and ERK1/2 at early and late time points under high NaCl conditions, indicating increased and sustained TCR signaling (Fig. 2h). We also detected an increase in the phosphorylation of PLCγ1, which is known to activate *ORAI1*, a pore component of the Ca^2+^ release-activated Ca^2+^ channels which ensures sustained intracellular Ca^2+^ increase^18,19^. Furthermore, we found that the expression of *ORAI1* was significantly upregulated under high NaCl conditions, as assessed by scRNA-seq (Fig. 2i).

These findings prompted us to investigate whether extracellular changes in NaCl concentrations affected TCR stimulation-induced calcium influx (Ca^2+^ flux). Human CD8^+^CD45RA^−^ T cells were expanded with CD3 and CD28 mAbs for 5 d in low and increasing NaCl concentrations before Ca^2+^ flux was assessed in real time at the single-cell level by flow cytometry after TCR crosslinking with a secondary F(ab′)_2_ fragment. Interestingly, we observed a significant dose-dependent enhancement of Ca^2+^ flux on TCR restimulation if CD8^+^ T cells were preactivated in increasing extracellular NaCl concentrations (Fig. 2j). Furthermore, we found that TCR engagement was necessary for NaCl-induced increase in Ca^2+^ flux, because no differences in intracellular Ca^2+^ concentrations were observed under high compared with low NaCl conditions at baseline recordings before TCR crosslinking (Fig. 2j).

We next performed high-dimensional spectral flow cytometry for multiple T cell activation- and differentiation-associated protein surface markers (33-marker panel, Supplementary Table 3) for CD8^+^CD45RA^−^ T cells stimulated under high and low NaCl conditions. UMAP analysis demonstrated distinct clustering of CD8^+^ T cells exposed to high and low NaCl conditions (Supplementary Fig. 9), similar to what we had observed at the transcriptomic level before. In particular, CD69, a classic marker of early leukocyte activation, was strongly upregulated under high NaCl conditions (Fig. 2k). This pattern also applied to human leukocyte antigen (HLA)-DR, another marker of human T cell activation and a proxy for recent cell division and cytotoxic effector functions^20^ (Fig. 2l). Other activation-associated molecules such as such as inducible stimulator ICOS (*ICOS*), CD103 (*ITGAE*) and programmed cell death protein 1 (PD-1; *PDCD1*), were also significantly upregulated at the protein and transcriptomic levels in response to TCR activation under high NaCl conditions (Fig. 2m and Supplementary Fig. 10a,b).

Several of the protein activation markers that we found to be significantly upregulated under high NaCl conditions, such as CD69, CD103 and CD49a, have previously been associated with T cell tissue residency^21^. In situ persistence of tumor-infiltrating lymphocytes as tissue-resident T cells (T_RM_ cells) has been shown before to contribute to superior protective potential of CD8^+^ T cells in solid cancers. It was interesting to find that the T_RM_ cell transcriptomic cell identity was indeed significantly enhanced on exposure to high NaCl concentrations (Supplementary Fig. 10c–g)^22,23^.

Given that several activation-associated markers, particularly the immune checkpoint inhibitors, play divergent context-dependent roles in CD8^+^ T cell activation and exhaustion, we specifically excluded differences in cellular exhaustion signatures under high and low NaCl culture conditions (Supplementary Fig. 10h)^24,25^. Furthermore, total T cell numbers did not differ on repetitive restimulations of CD8^+^CD45RA^−^ T cells with CD3 and CD28 mAbs in either high or low NaCl conditions (Supplementary Fig. 10i). Notably, repetitive cycles of restimulating CD8^+^CD45RA^−^ T cell clones with CD3 and CD28 mAbs over a long culture period of 30 d demonstrated sustained levels of increased granzyme B secretion under high NaCl conditions (Supplementary Fig. 10j). Prestimulation of CD8^+^ T cells under high compared with low NaCl conditions even resulted in a higher cloning efficiency, despite restimulation and expansion of single T cells with allogeneic peripheral blood mononuclear cells (PBMCs) in low NaCl conditions (Supplementary Fig. 10k). Cumulatively, these data support the viability, long-term maintenance and retention of CD8^+^ T cell effector functions on TCR stimulation under high NaCl culture conditions.

Although we focused our analyses on CD8^+^CD45RA^−^ effector T cells, which are known to constitute the main tumor-infiltrating T cell population, we could also prove an overall increased activation phenotype for naive CD8^+^CD45RA^+^ T cells when cultured under high NaCl conditions (Supplementary Fig. 11a–g).

In sum, these data suggest that high NaCl conditions potentiate CD8^+^ T cell activation at the transcriptomic and functional level.

### NaCl potentiates cytotoxic T cell effector functions

**Fig. 3:**
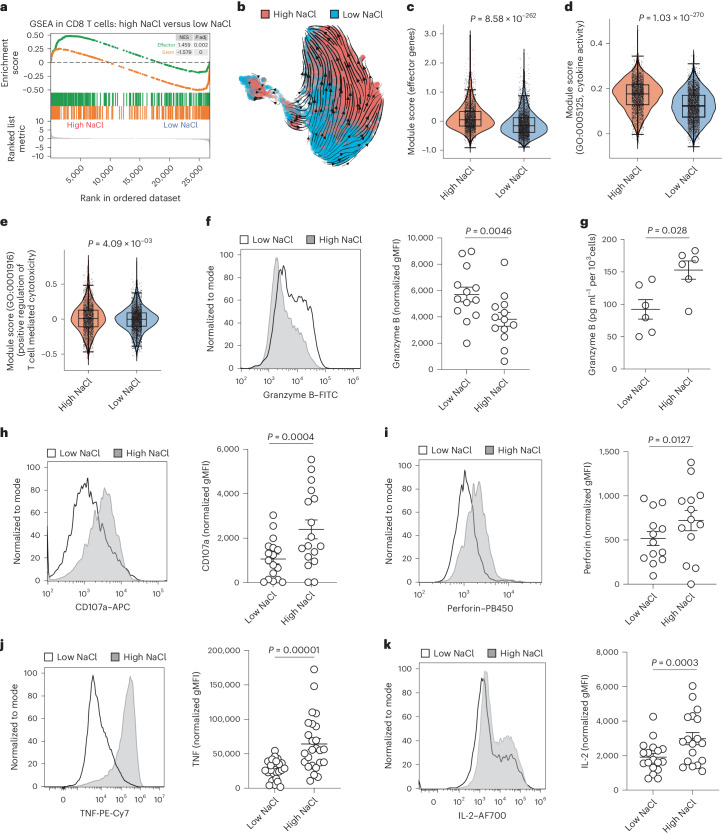
NaCl enhances CD8^+^ T cell effector function and cytotoxicity. **a**, GSEA for effector- and stemness-associated genes was performed after a transcriptomic comparison of bulk human CD8^+^CD45RA^−^ T cells stimulated with CD3 and CD28 mAbs for 5 d under high and low NaCl conditions. **b**, RNA velocity analysis after scRNA-seq of human CD8^+^CD45RA^−^ T cells stimulated with CD3 and CD28 mAbs for 3 d under high and low NaCl conditions. The velocities are shown in UMAP embedding. Cells are color coded according to the treatment condition as indicated. **c**–**e**, ScRNA-seq and analysis of the module scores of the indicated gene sets (**c**, effector genes; **d**, cytokine activity; **e**, positive regulation of T cell mediated cytotoxicity) for human CD8^+^CD45RA^−^ T cells stimulated with CD3 and CD28 mAbs for 3 d under high and low NaCl conditions (*n* = 1; Wilcoxon’s rank-sum test). **f**, Intracellular cytokine staining and flow cytometric analysis of human CD8^+^CD45RA^−^ memory T cells after stimulation for 5 d with CD3 and CD28 mAbs under high and low NaCl conditions. Left, representative experiment; right, cumulative data (*n* = 13; mean ± s.e.m., two-tailed, paired Student’s *t*-test). **g**, ELISA of cell culture supernatants from human CD8^+^ memory T cells stimulated for 5 d with CD3 and CD28 mAbs (*n* = 6; mean ± s.e.m., two-tailed, paired Student’s *t*-test). **h**, Flow cytometric analysis of human CD8^+^CD45RA^−^ T cells performed after stimulation for 5 d with CD3 and CD28 mAbs under high and low NaCl conditions. Left, representative experiment; right, cumulative data. Data present the mean ± s.e.m. (*n* = 17; two-tailed, paired Student’s *t*-test). **i**–**k**, Intracellular cytokine staining and flow cytometric analysis (**i**, perforin; **j**, TNF; **k**, IL-2) of human CD8^+^CD45RA^−^ T cells after stimulation for 5 d with CD3 and CD28 mAbs under high and low NaCl conditions and restimulation with PMA/ionomycin for 5 h. Left, representative experiment; right, cumulative data. Data present the mean ± s.e.m. (*n* = 25 (**j**), n = 18 (**k**); two-tailed, paired Student’s *t*-test).

We next investigated the effect of NaCl on specific CD8^+^ T cell effector functions. Transcriptome-wide analysis of bulk memory CD8^+^ T cells demonstrated a significant shift toward the effector cell identity, paired with reduced stemness on stimulation under high NaCl conditions (Fig. 3a). To explore the impact of NaCl on the differentiation state of CD8^+^ T cells in more detail at the single-cell level, we performed a trajectory inference analysis. RNA velocity demonstrated a clear differentiation trajectory for CD8^+^ T cells from low to high salt conditions (Fig. 3b). Diffusion pseudotime analysis corroborated that high NaCl conditions significantly advanced the differentiation state of human CD8^+^ T cells (Supplementary Fig. 12a). This finding was supported by higher expression of the ‘effector function’^26^ (Fig. 3c) and ‘cytokine activation’ modules (Fig. 3d) in CD8^+^ T cells from high compared with low NaCl conditions.

Notably, CD8^+^ T cells from high NaCl conditions also displayed an increased module score for the gene ontology (GO) term ‘positive regulation for T cell mediated cytotoxicity’ (Fig. 3e). Intracellular granzyme B protein expression was significantly reduced on CD8^+^ T cell stimulation under high NaCl conditions on day 5 (Fig. 3f). This finding reflected strong release of preformed granzyme B into the extracellular space, as demonstrated by both ELISA of cell culture supernatants (Fig. 3g) and significant upregulation of the degranulation marker CD107a and perforin (Fig. 3h,i). Therefore, these results indicate that NaCl potentiates the cytotoxic effector functions of human CD8^+^ T cells. These findings were corroborated in naive CD8^+^CD45RA^+^ T cells that were stimulated under high and low NaCl conditions (Supplementary Fig. 11g).

To further explore the functionality of human CD8^+^ T cells in high NaCl conditions, we next tested the effect of NaCl on CD8^+^ T cell-associated cytokines. Tumor necrosis factor (TNF) expression, which is known to induce cytotoxicity in select target cells^27^, was significantly increased in CD8^+^ T cells under high compared with low NaCl conditions (Fig. 3j). Likewise, we found increased production of interleukin (IL)-2 by CD8^+^ T cells under high NaCl conditions (Fig. 3k). Interferon (IFN)-γ expression in CD8^+^ T cells was, however, not influenced by the extracellular NaCl microenvironment (Supplementary Fig. 12b).

Together, these results demonstrate that NaCl potentiates the cytotoxic effector functions of human CD8^+^ T cells.

### NaCl boosts CD8^+^ T cell metabolic fitness

**Fig. 4:**
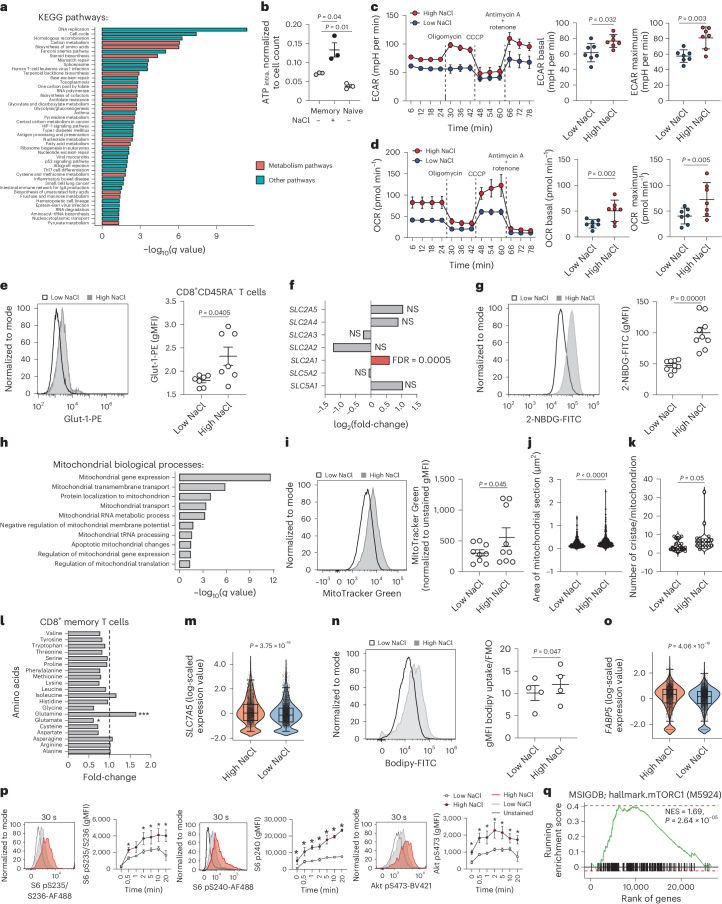
NaCl potentiates the metabolic fitness of CD8^+^ T cells. **a**, Enrichment by overrepresentation analysis for all 45 upregulated KEGG pathways (with Benjamini–Hochberg-adjusted *q* value ≤ 0.05) using significantly upregulated DEGs (*P* ≤ 0.05, log_2_(fold-change) ≥ 0.5) from the bulk transcriptomic comparison of human CD8^+^CD45RA^−^ T cells stimulated for 5 d with CD3 and CD28 mAbs under high and low NaCl conditions. **b**, Luminometric assessment of ATP production in human CD8^+^CD45RA^+^ and CD8^+^CD45RA^−^ cells, which were stimulated as described in **a**, normalized to ATP per cell (*n* = 3; mean ± s.e.m., two-tailed, paired Student’s *t*-test). **c**,**d**, Real-time analysis of the ECAR (**c**) and OCR (**d**) by human CD8^+^CD45RA^−^ T cells using a Seahorse Extracellular Flux Analyzer. The dotted lines show the time point of addition of the indicated substances. Left, representative experiment with technical replicates; right, cumulative quantification with individual healthy donors (*n* = 7; mean ± s.e.m. two-tailed, paired Student’s *t*-test). **e**,**g**,**i**,**n**, Flow cytometric analysis of human CD8^+^CD45RA^−^ T cells. Left, representative experiment; right, cumulative quantification (*n* = 7 (**e**), *n* = 9 (**g**), *n* = 9 (**i**), *n* = 4 (**n**); mean ± s.e.m., two-tailed, paired Student’s *t*-test). **f**, Expression of the indicated genes encoding glucose transporters in a transcriptomic comparison of bulk human CD8^+^CD45RA^−^ T cells stimulated for 5 d with CD3 and CD28 mAbs under high and low NaCl conditions (*n* = 3). **h**, Significantly enriched terms of GO-annotated, mitochondrial biological processes after overrepresentation analysis of upregulated genes from the bulk transcriptomic comparison of human CD8^+^CD45RA^−^ T cells stimulated as in **a**. The *q* values show *P*-value adjustment for multiple-test correction. Term redundancy was reduced using REVIGO (http://revigo.irb.hr, similarity parameter = 0.5). **j**,**k**, Transmission electron microscopy, number of mitochondria (**j**, *n* = 632 for low NaCl, 373 for high NaCl conditions) and cristae per mitochondrion (**k**, *n* = 18 for low NaCl, 21 for high NaCl) (two-tailed, unpaired Student’s *t*-test). The data represent two independent experiments. **l**, Nontargeted metabolic profiling (metabolome analysis) of CD8^+^CD45RA^−^ T cells stimulated as in **a** (*n* = 4 individual blood donors (matched samples); one-way ANOVA). **m**,**o**, ScRNA-seq analysis of cells cultured as in **a** (**m**, *SLC7A5*; **o**, *FABP5*) one biological replicate (Wilcoxon’s rank-sum test). **p**, Phospho-flow analysis of CD8^+^CD45RA^−^ T cells cells stimulated as in **a** after TCR crosslinking for the indicated time points (*n* = 3; mean ± s.e.m., two-way ANOVA with uncorrected Fisher’s LSD; ^*^P < 0.05). **q**, Preranked GSEA. One-tailed permutation test for positive enrichment is based on an adaptive multilevel split Monte Carlo scheme (R package FGSEA).

We considered the possibility of metabolic reprogramming of T cells by NaCl as a potential underlying mechanism of NaCl-enhanced effector functions. In line with this hypothesis, a multitude of cell metabolism-associated processes were overrepresented after a bulk transcriptomic comparison of memory CD8^+^ T cells stimulated under high and low NaCl conditions (Fig. 4a). We found that increased concentrations of extracellular NaCl resulted in a significant increase in intracellular ATP in CD8^+^CD45RA^−^ T cells on stimulation with CD3 and CD28 mAbs (Fig. 4b). This finding prompted an in-depth dissection of the metabolic perturbations induced by NaCl. We monitored the extracellular acidification rate (ECAR) and the cellular oxygen consumption rate (OCR) in CD8^+^ T cells in real time as measures of glycolysis and mitochondrial respiration, respectively, after 5 d of stimulation with CD3 and CD28 mAbs under high or low NaCl conditions. Interestingly, we found substantially increased ECAR (Fig. 4c) and OCR (Fig. 4d) values in CD8^+^CD45RA^−^ T cells that were preconditioned with high NaCl. We first focused on glycolysis. In line with this extracellular flux analysis, we found a significant overrepresentation of the glycolysis pathway by the transcriptomic comparison of bulk CD8^+^CD45RA^−^ T cells cultured under high versus low NaCl conditions (Fig. 4a). Furthermore, 18 out of 20 genes involved in the glycolysis pathway were significantly upregulated in the KEGG (Kyoto Encylopedia of Genes and Genomes) pathway analysis and on gene set enrichment analysis (GSEA) (Supplementary Fig. 13a,b). High NaCl conditions resulted in significantly increased expression of the glucose transporter Glut-1 on the cell surface (Fig. 4e). This finding was also consistent with the upregulation of *GLUT1* (*SLC2A1*) gene expression under high NaCl conditions (Fig. 4f). The expression of other glucose transporter genes, however, remained unaffected by NaCl (Fig. 4f). We then confirmed by flow cytometry that increased concentrations of extracellular NaCl could enhance cellular glucose uptake by promoting the entry of 2-NBDG, a fluorescent derivative of glucose (Fig. 4g)^28^. This effect was specific for NaCl ions because other osmolytes, such as mannitol, urea, MgCl_2_ and sodium gluconate, did not alter 2-NBDG uptake (Supplementary Fig. 14a). We then applied liquid chromatography (LC) coupled to mass spectrometry (MS) to perform a comprehensive metabolite analysis of human memory CD8^+^ T cells (metabolome with 489 compounds of known identity) after stimulation with CD3 and CD28 mAbs under high and low NaCl conditions. This analysis confirmed the significant increase of intracellular glucose levels in these cells. This effect was specific for CD8^+^CD45RA^−^ T cells because CD4^+^CD45RA^−^ did not demonstrate increased intracellular glucose on exposure to high NaCl concentrations (Supplementary Fig. 14b).

Given the increased oxygen consumption rates, we next focused on mitochondrial metabolism of CD8^+^ T cells in response to high NaCl conditions. Na^+^ has previously been shown to act as a second messenger with effects on OXPHOS function and redox signaling via the NCLX Na^+^/Ca^2+^ exchanger^29^. Our transcriptomic enrichment analysis showed upregulation of multiple functional terms associated with mitochondrial metabolism in response to high NaCl concentrations (Fig. 4h). Analysis of MitoTracker Green staining by flow cytometry validated that CD8^+^ T cell stimulation in high NaCl microenvironments resulted in increased mitochondrial mass consistent with the enhanced ATP production and glucose uptake (Fig. 4i). This finding was also supported by electron microscopy, which demonstrated a significantly increased mitochondrial size and a higher number of mitochondrial cristae in CD8^+^CD45RA^−^ T cells cultured under high compared with low NaCl conditions (Fig. 4j,k and Supplementary Fig. 15).

Metabolomic analysis using LC and MS demonstrated that, out of 20 protein building amino acids, exclusively glutamine was significantly enriched in human memory CD8^+^ T cells on stimulation under high NaCl conditions (Fig. 4l). Consistent with these findings, transcriptomic analyses revealed increased expression of the *SLC7A5* glutamine transporter under high NaCl conditions (Fig. 4m and Supplementary Table 1) as well as *MYC*, the transcription factor driving glutamine usage (Fig. 2b and Supplementary Table 1)^12,30^. Glutamine is known to be involved in the activation of mTOR^31^ and to increase in abundance on TCR activation. To study glutaminolysis, we performed stable-isotope tracing to determine tricarboxylic acid (TCA) cycle fluxes. The applied [U-^13^C-5] glutamine tracer enters the TCA cycle at α-ketoglutarate and can then be metabolized by either oxidative or reductive TCA metabolism (Supplementary Fig. 16a). We found that NaCl induces the contribution of glutamine carbon to TCA cycle intermediates (Supplementary Fig. 16b), confirming increased glutaminolysis. Increased M4 isotopologues of fumarate, malate, aspartate and citrate indicate an increased glutaminolysis through oxidative TCA cycle metabolism (Supplementary Fig. 16c). NaCl did not change reductive carboxylation through isocitrate dehydrogenase 1 as shown by unchanged fractions of M5 citrate and M3 aspartate isotopologues (Supplementary Fig. 16d). Consistent with these findings, glutamate, which has previously been shown to inhibit cytotoxic T cell differentiation^32^, was significantly decreased intracellularly under high NaCl conditions, as shown by metabolomic analysis (Fig. 4l).

To investigate lipid metabolism, we tested uptake of the fluorescently labeled lipid analog BODIPY FL C16 by flow cytometry. We found that high NaCl concentrations induced significantly enhanced uptake of BODIPY FL C16 by CD8^+^CD45RA^−^ T cells (Fig. 4n). This finding was consistent with augmented *FABP5* expression on transcriptomic comparison of single cells stimulated in high and low NaCl conditions (Fig. 4o).

Given the role of mTOR as a central hub in immunometabolism, we specifically investigated induction of mTOR signaling by phosphorylation of the ribosomal subunit S6 after TCR crosslinking of CD8^+^CD45RA^−^ T cells that were prestimulated under high and low NaCl conditions. We observed significantly increased phosphorylation of the S6 residues S235/S236 and S240 under high NaCl conditions at early time points (30 s) and also stably sustained phosphorylation at late time points (20 min) (Fig. 4p). Akt, the serine/threonine kinase acting upstream of mTOR, displayed the same phosphorylation pattern, indicating overall increased Akt-mTOR signaling (Fig. 4p). A GSEA analysis (hallmark mTORC1) supported increased NaCl-induced mTOR signaling (Fig. 4q). Furthermore, a protein–protein interaction (PPI) network analysis with curated PPI information and integration of our bulk gene expression fold-changes revealed a significant correlation of mTOR with the NaCl-sensing molecule SGK1 (Supplementary Fig. 17a,b).

Cumulatively, these analyses demonstrated an augmented uptake of nutrients under high NaCl conditions and profound metabolic rewiring of human CD8^+^CD45RA^−^ cells.

### NaCl hyperpolarizes the membrane potential through Na^+^/K^+^-ATPase

**Fig. 5:**
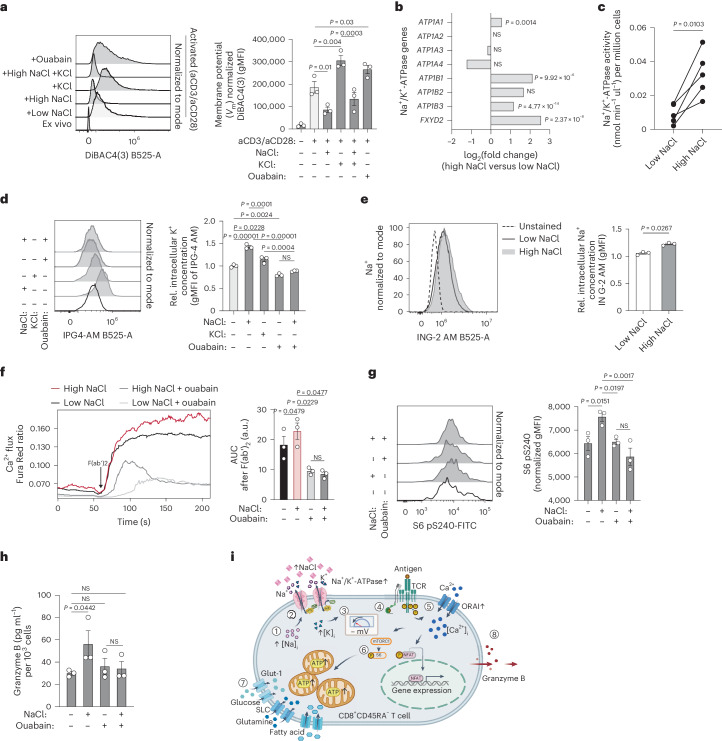
NaCl enhances membrane hyperpolarization through Na^+^/K^+^-ATPase. **a**, Flow cytometric analysis of the membrane potential of human CD8^+^CD45RA^−^ T cells after stimulation for 5 d with CD3 and CD28 mAbs under high and low NaCl conditions in the presence or absence of ouabain treatment for 1 h before analysis (*n* = 3; mean ± s.e.m., one-way ANOVA with Fisher’s LSD test). **b**, ATPase gene expression. Bulk mRNA-seq analysis of CD8^+^CD45RA^−^ T cells stimulated under high and low NaCl conditions with CD3 and CD28 mAbs for 5 d. Multiple test-adjusted *P* value and log_2_(fold-changes) according to DESeq2 test statistics are given over all transcriptome data (*n* = 3). **c**, Colorimetric analysis of the Na^+^/K^+^-ATPase activity of CD8^+^CD45RA^−^ T cells treated under high and low NaCl conditions as in **b** in the presence or absence of ouabain (*n* = 5; two-tailed, paired Student’s *t*-test). **d**,**e**, Flow cytometric analysis of intracellular K^+^ (**d**) and Na^+^ (**e**) for cells treated as in **a**. rel, relative. **f**, Calcium flux analysis with flow cytometry as in Fig. 2j before and after TCR crosslinking of CD8^+^CD45RA^−^ T cells stimulated as in **a** (one-way ANOVA). **g**, Phospho-flow cytometry of CD8^+^CD45RA^−^ T cells stimulated as in **a** (one-way ANOVA). **h**, ELISA of 1-h cell culture supernatants on day 5 of CD8^+^CD45RA^−^ T cells stimulated as in **a** (*n* = 3; mean ± s.e.m., one-way ANOVA with Fisher’s LSD test). **i**, Overview of molecular mechanism of NaCl-induced T cell hyperactivation.

To understand the molecular mechanism by which a recent history of NaCl exposure can translate TCR engagement in human CD8^+^ T cells into enhanced T cell activation, we explored the hypothesis of whether increased extracellular NaCl concentrations would lead to magnified electromotive forces for cytosolic Ca^2+^ ion entry after TCR-induced opening of calcium (ORAI) channels^33^. The electrochemical gradient induced by the membrane potential (*V*_m_) is known to directly drive Ca^2+^ permeation through activated ORAI channels^33,34^. We found that stimulation of CD8^+^CD45RA^−^ T cells with CD3 and CD28 mAbs led to depolarization of the *V*_m_ as assessed by flow cytometry with the potential-sensitive probe DiBAC_4_(3), in line with previous reports^35^. Strikingly, this depolarization was considerably decreased under high NaCl conditions, leading to a relative hyperpolarization of the *V*_m_ under high compared with low NaCl conditions (Fig. 5a). Stimulation of CD8^+^CD45RA^−^ T cells in hyperkalemic conditions (+40 mM KCl), which have previously been shown to paralyze T cell responses^2^, resulted in further depolarization of the membrane potential, instead (Fig. 5a). It is interesting that additional extracellular NaCl was able to counterregulate the KCl-induced membrane depolarization (Fig. 5a). As expected, disruption of the ionic gradients, which maintain the electrical charge across membranes, with the pharmacological inhibitor of the Na^+^/K^+^-ATPase ouabain, resulted in a membrane depolarization that resembled the hyperkalemic culture condition.

We next explored the molecular basis of the NaCl-induced relative hyperpolarization of the *V*_m_. The Na^+^/K^+^-ATPase is known to participate in establishing a negative intracellular membrane potential by exporting three Na^+^ ions out of the cell in exchange for two K^+^ ions entering it^36^. The bulk and single-cell transcriptomic comparison of CD8^+^CD45RA^−^ T cells from high and low NaCl conditions revealed a significant increase in transcripts encoding the α (*ATPA1*) and β subunits (*ATPB1*, *ATPB3*) as well as the regulatory subunit of the Na^+^/K^+^-ATPase (*FXYD1*) (Fig. 5b and Supplementary Fig. 18). We furthermore assessed the biological activity of the Na^+^/K^+^-ATPase in response to high and low NaCl conditions by its ATP consumption in the presence or absence of the Na^+^/K^+^-ATPase-specific inhibitor ouabain. This functional analysis demonstrated that high NaCl conditions strongly augmented the activity of the Na^+^/K^+^-ATPase (Fig. 5c). In accordance with the Na^+^/K^+^-ATPase’s specialization for K^+^ import, the NaCl-enhanced activity of the Na^+^/K^+^-ATPase was accompanied by a significant increase of intracellular K^+^ concentrations (Fig. 5d). K^+^ ions have previously been shown to translocate into the intracellular space under hyperkalemic conditions^2^. It is interesting that NaCl even outperformed KCl in intracellular K^+^ accumulation at equimolar concentrations (Fig. 5d). Loss of intracellular K^+^ ions on ouabain treatment could not be restored by additional extracellular NaCl, highlighting the critical role of the Na^+^/K^+^-ATPase for the NaCl-mediated intracellular K^+^ accumulation (Fig. 5d).

We next addressed the mechanism by which high NaCl concentrations induced Na^+^/K^+^-ATPase activity. As previous reports have established intracellular Na^+^ as a driving force for Na^+^/K^+^-ATPase activity^37^, we hypothesized that increased levels of extracellular Na^+^ translocated into the intracellular space augmented Na^+^/K^+^-ATPase activity. We found that stimulation of CD8^+^CD45RA^−^ T cells under high compared with low NaCl conditions indeed resulted in increased intracellular Na^+^ concentrations as quantified by flow cytometry (Fig. 5e).

Finally, we explored whether the enhanced T cell signaling, metabolism and effector functions under high NaCl conditions could be reversed by inhibition of Na^+^/K^+^-ATPase. We found that inhibition of Na^+^/K^+^-ATPase with ouabain for 1 h before TCR crosslinking abrogated the NaCl-enhanced Ca^2+^ flux (Fig. 5f), which was consistent with a reduced electrochemical Ca^2+^ permeation gradient on reduced membrane hyperpolarization. Na^+^/K^+^-ATPase inhibition also abrogated NaCl-induced mTOR signaling as seen by hypophosphorylation of S6 in the concomitant presence of NaCl (Fig. 5g). Furthermore, granzyme B secretion of NaCl-pretreated CD8^+^CD45RA^−^ T cells was reduced after inhibition of Na^+^/K^+^-ATPase (Fig. 5h).

Cumulatively, our data propose a mechanism for NaCl-induced T cell hyperactivation that involves intracellular translocation of Na^+^, followed by increased Na^+^/K^+^-ATPase activity, *V*_m_ hyperpolarization and thus increased electromotive forces for TCR-induced entry of Ca^2+^, the upstream second messenger for T cell activation, metabolic reprogramming and finally for enhanced cytotoxic effector functions (Fig. 5i).

### NaCl promotes tumor cell killing in humans and mice

**Fig. 6:**
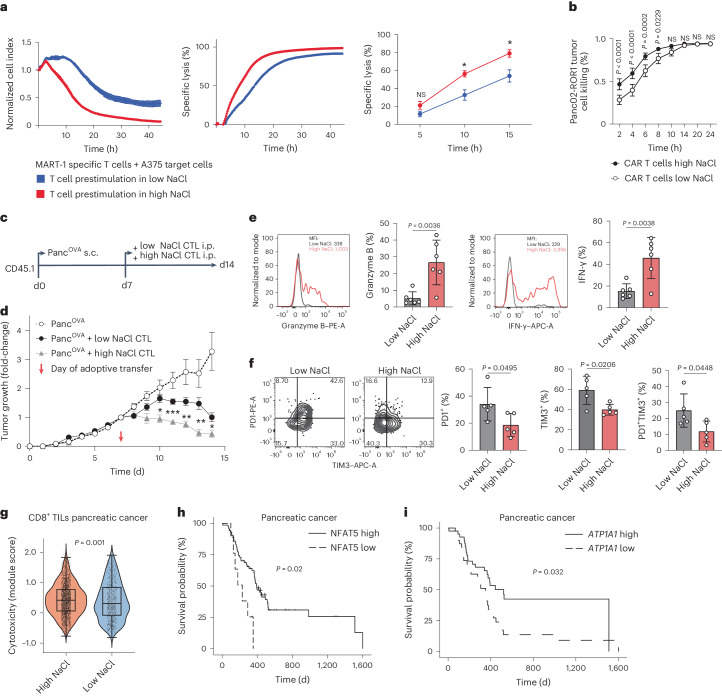
NaCl licenses CD8^+^ T cells for killing of tumor cells in vitro and in vivo*.* **a**, Real-time killing assay with nucleofected MART-1-specific T cells and A375 melanoma cell target cells at a 1:1 ratio under high and low NaCl conditions using the xCELLigence technology. Left, the normalized cell index; middle, the specific lysis; right, the cumulative quantification of 3T cell donors (*n* = 3 experiments; mean ± s.e.m.; two-way ANOVA, ^*^P < 0.05). **b**, Murine ROR1 CAR T cells generated and cultured for 48 h under high and low NaCl conditions and then cocultured with ROR1-expressing target cells at a 10:1 ratio. Antigen-specific lysis of Panc02-ROR1 cells by CD8^+^ CAR T cells was determined at different time points (*n* = 3 independent experiments; mean ± s.d., two-way ANOVA). **c**, Experimental design. **d**, The tumor growth curves of subcutaneous tumors. Tumor growth was normalized to the tumor size on the day of CD8^+^ T cell injection (*n* = 7 (PancOVA), *n* = 6 (PancOVA + low NaCl control (CTL)), *n* = 6 (PancOVA + high NaCl CTL); mean ± s.e.m. two-way ANOVA with Tukey’s honestly significant difference (HSD), multiple-comparison test). **e**,**f**, Flow cytometric analysis of intratumoral CD45.2^+^CD8^+^ T cells 72 h after T cell transfer (*n* = 6 (**e**), *n* = 5 (**f**); mean ± s.d., two-tailed, unpaired Student’s *t*-test). **g**, ScRNA-seq and module score calculation for T cell cytotoxicity genes obtained from published reports^55,56^, validated with genes from GO:0001916 (*P* = 0.01). Intratumoral CD8^+^ T cells are shown from 56 patients with pancreatic cancer (from accession nos. GSE155698, GSE111672, GSE154778, GSM4293555 and PRJCA001063)^40^, integration of all cells: 10.5281/zenodo.6024273. CD8^+^ T cells were categorized into cells with a high and low NaCl signature based on the NaCl signature obtained from scRNA-seq of CD8^+^CD45RA^–^ T cells treated under high versus low NaCl concentrations (top 60 upregulated DEGs; Supplementary Table 2; cutoff defined as module score ≥0 and <0 for high versus low NaCl signature, respectively; Wilcoxon’s rank-sum test). **h**,**i**, Kaplan–Meier tumor-free survival probability of patients from TCGA database diagnosed with pancreatic cancer. Patients were subgrouped by computing an optimal cutoff for *NFAT5* (**h**) and *ATP1A1* (**i**) expression. TPM values were normalized toward overall survival outcome. Number of patient samples: pancreatic cancer: *n* = 72 for *NFAT5* high, *n* = 9 for *NFAT5* low; *n* = 41 for *ATP1A1* high; *n* = 40 for *ATP2A2* low; significance of survival differences was determined using the Peto–Peto algorithm with the surv_pvalue function (method = ‘S1’) as implemented in the R package survminer.

The NaCl-induced increases in effector functions and their metabolic correlates prompted an investigation into the overall killing capacity of human CD8^+^ T cells in the context of elevated NaCl concentrations. We therefore generated antigen-specific CD8^+^ memory T cells via nucleofection of a MART-1-specific TCR. These TCR-engineered T cells displayed enhanced granzyme B secretion and activation marker upregulation on stimulation with CD3 and CD28 mAbs under high compared with low NaCl conditions (Supplementary Fig. 19a). Cells that stained positive or negative for the MART-1 TCR after nucleofection, as assessed by detection of the concomitantly introduced murine TCR β-chain, were prestimulated under high and low NaCl conditions before coculture with adherent MART-1-expressing A375 melanoma cells at a 1:1 ratio. Stable maintenance of the newly introduced TCR in cells subjected to both extracellular NaCl concentrations was confirmed by flow cytometry (Supplementary Fig. 19b,c). Killing of tumor cells was monitored in real time using xCELLigence technology. We observed a strong increase in specific melanoma target cell lysis by CD8^+^ T cells that were prestimulated for 5 d under high NaCl conditions (Fig. 6a). We then cocultured T cells and MART-1 peptide-pulsed melanoma cells in the concomitant presence of high extracellular NaCl concentrations and observed a strong increase in melanoma cell lysis under high NaCl conditions (Supplementary Fig. 19d). We could rule out a direct effect of NaCl on melanoma cell growth or lysis (Supplementary Fig. 19e,f) and therefore attributed this finding to the effect of NaCl on antigen-specific CD8^+^ T cells. This ability of NaCl to promote CD8^+^ T cell cytotoxicity was also corroborated with MART-1-specific T cell clones, which we selected from the natural repertoire of healthy human HLA-A2-seropositive donors by tetramer staining (Supplementary Fig. 19g). This enhanced killing ability could be stably maintained in CD8^+^ T cells prestimulated with high NaCl even after withdrawal from a high NaCl microenvironment (Supplementary Fig. 19h).

We then explored the possibility of improving CAR T cell cytotoxicity with NaCl. We engineered T cells to express a second-generation CAR containing a 4-1BB-derived costimulatory domain that specifically recognized the pancreatic cancer cell line Panc02-mROR1. Tumor target cell killing was improved if CAR T cells were prestimulated under high compared with low NaCl conditions (Fig. 6b). As the choice of the costimulatory domain within the CAR construct could potentially alter cellular metabolism and T cell effector functions^38,39^, we additionally assessed the impact of NaCl on CAR T cells with a CD28-derived costimulatory domain. This demonstrated superior tumor cell killing under high NaCl conditions as well (Supplementary Fig. 20). Hence, the application of NaCl might be suitable for both CD28- and 4-1BB-based CARs.

Taken together, these findings demonstrate that the tumor antigen-specific cytotoxicity of human CD8^+^ T cells is enhanced in microenvironments with increased NaCl concentrations. This is uniformly applied to T cells from the natural T cell repertoire, TCR-engineered transgenic T cells and CAR T cells.

Given the enhanced cytotoxicity that was exerted by NaCl on T cells in vitro, we asked whether NaCl could be exploited for the enhancement of CD8^+^ T cell cytotoxicity during adoptive T cell therapies in vivo. To this end, we chose a well-established pancreatic cancer mouse model and used the pancreatic cancer cell line Panc^Ova^, which expresses the model antigen ovalbumin. Naive CD8^+^ T cells were obtained from OT-I mice, in which CD8^+^ T cells are specific for OVA_257–264_. These cells were stimulated in vitro with CD3 and CD28 mAbs under high (+30 mM) or low NaCl conditions for 3 d, washed and then adoptively transferred into mice with tumors established 7 d earlier after subcutaneous injection of 1 × 10^6^ Panc^OVA^ cells (Fig. 6c). Compared with untreated mice, the mice treated with CD8^+^ T cells stimulated with low NaCl concentrations rejected the tumors, as evidenced by a reduced tumor volume (Fig. 6d). Remarkably, tumor rejection was significantly increased after transfer of CD8^+^ T cells from high NaCl conditions (Fig. 6d). NaCl had no effect on the CD8^+^ T cell proliferation or viability in vitro (Supplementary Fig. 21a,b). Similar to the results observed in human CD8^+^ T cells, murine CD8^+^ T cells released significantly more granzyme B into the supernatant on differentiation under high rather than low NaCl conditions (Supplementary Fig. 21c). On day 3 of differentiation, murine CD8^+^ T cells also demonstrated enhanced intracellular granzyme B levels under high NaCl conditions compared with low NaCl conditions, but there was no difference in the levels of other CD8^+^ T cell effector cytokines, such as IFN-γ or TNF (Supplementary Fig. 21d–f). Importantly, ex vivo analysis of tumor-infiltrating T cells that were identified by their congenic CD45.2 marker expression demonstrated strongly increased effector functions, such as granzyme B and IFN-γ expression, if the mice received T cells that were pretreated under high conditions (Fig. 6e). In addition, intratumoral T cells of mice receiving T cells from high NaCl pretreatment also displayed a significantly reduced expression of exhaustion markers, such as PD-1 and TIM3, compared with intratumoral T cells from the control mice receiving T cells pretreated under low NaCl conditions (Fig. 6f). In sum, these data demonstrate that NaCl equips CD8^+^ T cells with improved antitumor functions in vivo.

Finally, we tested whether T cell cytotoxicity was also enhanced in the human tumor microenvironment in response to NaCl exposure. To this end, we interrogated the expression of the transcriptomic NaCl signature, which we had generated earlier, in CD8^+^ TILs from 56 patients with pancreatic cancer (10.5281/zenodo.6024273) (Supplementary Fig. 22a,b)^40^. We found that only a subgroup of TILs within the tumor microenvironment displayed a transcriptomic response to NaCl (2,511 TILs versus 776 TILs). These NaCl-sensing TILs demonstrated a significantly increased expression of the cytotoxic gene signature (Fig. 6g).

Finally, to assess the role of NaCl in the tumor microenvironment for cancer patient survival, we queried TCGA for metadata of patients with pancreatic adenocarcinoma and their correlation with the intratumoral expression of NaCl-responsive genes. *NFAT5* has previously been demonstrated to be a transcriptional sensor of extracellular NaCl^4,9,41^. Accordingly, we could demonstrate increased *NFAT5* gene expression and enhanced intranuclear translocation of NFAT5 protein by CD8^+^ memory T cells under high NaCl conditions (Supplementary Fig. 23a–c). Strikingly, we found that patients who displayed high intratumoral *NFAT5* gene expression at the time of diagnosis displayed significantly prolonged survival compared with patients with low intratumoral *NFAT5* expression (Fig. 6h). This finding was corroborated with a breast cancer patient cohort of TCGA (Supplementary Fig. 23d). It is interesting that higher gene expression of *ATP1A1*, which encodes the Na^+^/K^+^-ATPase that we showed regulating Na^+^-induced T cell activation, was also found to positively correlate with survival of patients with pancreatic cancer (Fig. 6i). Cumulatively, these results highlight the beneficial effect of NaCl within the tumor microenvironment for antitumor T cell immunity in vivo and for harnessing cytotoxicity in adoptive T cell therapies.

## Discussion

We have discovered that high NaCl concentrations in the extracellular microenvironment have a significant impact on cellular metabolism and enhance human CD8^+^ T cell effector functions through a Na^+^/K^+^-ATPase-dependent molecular mechanism. This leads to improved antitumor cytotoxicity in vitro and in vivo.

We have discovered that sodium is a functionally active determinant of the tumor microenvironment with profound effects on the overall transcriptome of the tumor tissue, and on CD8^+^ TILs in particular. Remarkably, almost all tumor entities from TCGA demonstrated evidence of a transcriptomic sodium imprint compared with healthy control tissues, pointing to sodium as a relevant, previously overlooked, constituent of the tumor microenvironment.

Increased NaCl-related transcriptional changes correlated with enhanced cytotoxicity. T cells in spatial proximity to higher NaCl concentrations may therefore have an advantage for tumor control in an otherwise overall immunosuppressive microenvironment. This raises the question about the driving factors and mechanism of sodium deposition in tumors. We have previously shown in different healthy human tissues, such as skin and muscle, that tissue sodium concentrations in humans correlate with the level of glycosaminoglycan (GAG) deposition^10^. These negatively charged macromolecules can locally trap cations such as Na^+^. Interestingly, GAGs have been reported to have increased abundance in tumor tissues^42^. In addition, the osmosensitive transcription factor NFAT5 has previously been described as regulating GlcAT-1, a key enzyme for GAG biosynthesis^43^. Other mechanisms of Na^+^ accumulation in human tumors, including dietary influences, as previously suggested in grafted tumor mouse models, could potentially contribute to local sodium accumulation and remain to be investigated in the future^44,45^.

An intriguing observation of the present study was the metabolic burst in human CD8^+^ T cells exposed to high NaCl concentrations, which was associated with potentiated T cell cytotoxicity in vitro and in vivo. This finding was unexpected, considering that T cells in tumor microenvironments are often found to be paralyzed owing to chronic stimulation and exposure to immunosuppressive signals. Contrarily, perturbation of mitochondrial respiration in T_reg_ cells on exposure to high NaCl concentrations has recently been reported^7^. Although CD8^+^ T cells generate more ATP through engagement of multiple layers of cellular metabolism, high NaCl was reported to decrease the metabolic fitness^46^ of T_reg_ cells through inhibitory effects on mitochondrial respiration^7^. As a consequence, T_reg_ cells lose their suppressive functions. These opposing effects of NaCl on the cellular metabolism of both T cell subsets could be the result of previously well-characterized intrinsic differences in their baseline metabolic identity, with T_reg_ cells depending on lipid oxidation and conventional T cells depending on glycolytic activity to exert their assigned functions^47^. This distinct metabolic wiring of CD8^+^ T cells in response to NaCl also translates into a differential dependence on Glut-1, the glucose importer, which we found to be strongly upregulated by NaCl in CD8^+^ memory T cells at the transcript and protein levels^46^. Notably, Glut-1 contains a consensus site ((95)Ser) for phosphorylation by SGK1 (serine/threonine-protein kinase 1)^48^, the intracellular sensor for NaCl, which we also found to be upregulated in high NaCl conditions. Our protein network analysis also revealed direct interaction between SGK1 and mTOR, a central hub for immunometabolism. This finding argues for a direct mechanistic link across NaCl sensing, glucose metabolism and glutaminolysis^49^. In addition, experimental differences, such as short versus sustained NaCl exposure in T_reg_ and CD8^+^ T cells, respectively, could account for the differential impact of NaCl on these different cells^7,50^. Notably, the previously reported loss of T_reg_ cell-suppressive functions and the herein reported potentiation of CD8^+^ T cell cytotoxicity are expected to contribute jointly to improved tumor elimination despite alternative cellular engagement of metabolic pathways in these distinct immune cell types^51^.

We have deciphered the molecular mechanism, which translates increased levels of extracellular NaCl into enhanced proximal TCR signaling. The Na^+^/K^+^-ATPase emerged as the central molecular hub connecting extracellular NaCl with TCR-dependent T cell responses. Its expression and activity increased under high NaCl conditions after translocation of Na^+^ into the cytoplasm. This finding is in line with early studies from the 1970s and 1980s demonstrating activation of the Na^+^/K^+^-ATPase by intracellular accumulation of Na^+^^37^. We showed that increased Na^+^/K^+^-ATPase activity resulted in relative hyperpolarization of the membrane potential and thus enhanced electromotive forces for Ca^2+^ entry after TCR engagement and subsequent opening of ORAI channels. Enhanced Ca^2+^ flux is known to exert a positive feedback on CD3 phosphorylation and may thus amplify proximal TCR signaling^52^. The Na^+^-enhanced activity of the Na^+^/K^+^-ATPase was accompanied by an increase in intracellular K^+^, which has recently been reported to counteract T cell exhaustion^53^. NaCl exerts its immunostimulatory effects via the Na^+^/K^+^-ATPase only if coupled to TCR stimulation, because no baseline changes in intracellular Ca^2+^ were observed under high NaCl conditions. Specific inhibition of the Na^+^/K^+^-ATPase with ouabain abrogated the Ca^2+^ flux augmented by NaCl as well as mTOR and thus metabolic signaling.

The significant reduction in tumor size on adoptive transfer of cytotoxic T lymphocytes (CTLs) stimulated under high NaCl conditions in the tumor mouse model of pancreatic cancer was compelling. We chose an adoptive T cell transfer model to pinpoint NaCl-induced immunomodulation of T cells and to exclude potential systemic effects of NaCl on the microbiota, the malignant cells themselves or the immunosurveillance of other cell types. Our in vivo data suggest an easy and safe actionable strategy to dramatically increase T cell cytotoxicity via metabolic regulation, with beneficial effects for cancer and possibly also chronic infections. Our results showing increased antigen-specific tumor cell killing with CAR T cells that were precultured in high NaCl conditions support such a therapeutic strategy. Increased T cell effector functions, however, come at the expense of stemness and thus long-term maintenance of tumor antigen- or pathogen-specific memory T cells^3^. Although we have ruled out T cell exhaustion under high NaCl conditions by multiple criteria, it should also be noted that continued NaCl deposition in tumors might potentially favor T cell exhaustion, warranting clinical trials to assess short-term and long-term effects of NaCl for antitumor immunity^54^.

In summary, these findings advance our understanding of CD8^+^ T cell regulation, expose a new mechanism for modulating T cell activation and may lead to new therapeutic strategies for the treatment of cancer and other pathologies that may benefit from augmented T cell cytotoxicity.

## Methods

### Cell purification and sorting

PBMCs from fresh peripheral blood of healthy donors were isolated by density gradient centrifugation using Ficoll-Paque Plus (GE Healthcare). Cytotoxic T cells and T_H_ cells were isolated from the PBMCs by positive selection with human CD8-specific MicroBeads (Miltenyi Biotec), respectively, using an autoMACS Pro Separator (Miltenyi Biotec). Memory T cells were isolated as CD45RA^−^ lymphocytes, and naive T cells were isolated as either CD45RA^+^CD45RO^−^CCR7^+^ lymphocytes or CD45RA^+^ T cells for scRNA-seq with a purity of >98%. The antibodies used for flow cytometry cell sorting have been described previously^57–59^. The cells were sorted with a BD FACSAria III (BD Biosciences), a BD FACSAria Fusion (BD Biosciences) or a Cytek Aurora CS.

### Cell culture

Human T cells were cultured in Roswell Park Memorial Institute (RPMI)-1640 medium supplemented with 1% (v:v) GlutaMax, 1% (v:v) nonessential amino acids, 1% (v:v) sodium pyruvate, penicillin (50 U ml^−1^), streptomycin (50 μg ml^−1^) (all from Invitrogen) and 10% (v:v) fetal calf serum (FCS; Sigma-Aldrich). For antigen-specific assays, FCS was replaced with 5% human serum (Sigma-Aldrich). Hypersalinity (high NaCl) was induced by increasing the NaCl concentration to 185 mM as described previously, unless indicated otherwise^4,25^. The final sodium concentrations in the cell culture medium were confirmed by potentiometry with a Cobas 8000 analyzer (Roche). T cells were stimulated with plate-bound CD3 (1 μg ml^−1^, clone TR66, Enzo Life Sciences) and CD28 mAbs (1 μg ml^−1^, clone CD28.2, BD Biosciences). T cell clones were generated under nonpolarizing conditions, as described previously, after single-cell deposition via flow cytometry-assisted cell sorting or by limiting dilution plating^59,60^.

### Generation and isolation of antigen-specific T cells

To obtain tumor antigen-specific T cells (MART-1 specificity), 1 × 10^7^ freshly isolated PBMCs were washed twice in phosphate-buffered saline (PBS) and resuspended in pre-equilibrated nucleofection buffer 1SM^61^. Then 20 μg of transposon-coding MART-1-specific TCR/0.5 µg of transposase SB100X solution was added to the cell suspension, which was subsequently transferred into the electrophoresis chamber. Nucleofection was performed on a Lonza Nucleofector IIb. After nucleofection, the cell suspension was transferred into a 24-well microplate containing complete culture medium with human serum and 50 U ml^−1^ of IL-2. After 24 h, MART-1-specific T cells were isolated from nucleofected PBMCs with a BD FACSAria III cell sorter by selecting CD3-FITC and anti-mouse-TCR β-chain APC (antigen-presenting cell)-positive cells after exclusion of dead cells with propidium iodide (PI).

MART-1-specific CD8^+^ T cells from the natural T cell repertoire were isolated from PBMCs from an HLA-A2-seropositive healthy donor after tetramer staining in MACS buffer (PBS supplemented with 1% (v:v) FCS, 2 mM EDTA) at 4 °C for 30 min. Tetramers were assembled the day before by incubating the peptide major histocompatibility complex (pMHC) with streptavidin–APC or streptavidin–BV421 overnight at 4 °C. The tetramers used for staining were pMHC–streptavidin–BV421 and pMHC–streptavidin–APC. The antibodies used for staining were CD3-FITC, CD8-PE (CD8-phycoerythrin) and CD19-ECD (CD19-extracellular domain). To exclude dead cells, PI was used. Single-cell sorting was carried out on a MoFlo Astrios Cell sorter (Becton Dickinson) after gating on single CD3^+^CD8^+^CD19^−^PI^−^ pMHC–streptavidin–APC^+^/pMHC–streptavidin–BV421^+^ lymphocytes. The pMHC complexes loaded with MART-1 peptide were refolded as described previously^9,62^. Antigen-specific T cell clones were generated as described previously^4,57^.

### Cytotoxicity assay

The viability of A375 melanoma cells (target cells) was determined in real time using an xCELLigence SP Real-Time Cell Analyzer (ACEA Biosciences), which allowed the quantitative and continuous monitoring of adherent A375 melanoma cells (target cells) through the measurement of electrical impedance every 15–30 min. For baseline measurements, 100 µl of A375 growth medium was added to the 96-well E-plate. A375 melanoma cells were seeded on to the E-plate at a density of 5 × 10^3^ cells per 100 µl of growth medium. After 24 h, 100 µl of culture medium was replaced by 10^−7^ M MART-1 peptide in 100 µl of fresh medium. After 1 h of incubation with the peptide, MART-1-specific CD8^+^ T cells, which had been preactivated in high or low NaCl conditions for 5 d (or no T cells as a control), were added at a 1:1 ratio in 100 µl after removal of an equal amount of the growth medium. All conditions were set up in technical replicates. Cell indices were monitored every 15–30 min for another 24–48 h on the xCELLigence System at 37 °C with 5% CO_2_. Values are represented as cell indices (CIs), CIs normalized to the start of the coculture (nCIs) or as cell lysis calculated according to the following formula: ${\rm{Cell}}\,{\rm{lysis}}=\frac{{\rm{n}}{\rm{CI}}({\rm{A}}375\,{\rm{only}})-{\rm{n}}{\rm{CI}}({\rm{sample}})}{{\rm{n}}{\rm{CI}}({\rm{A}}375\,{\rm{only}})}$.

### Flow cytometric analysis

For intracellular cytokine staining, human T cells were restimulated for 5 h with phorbol 12-myristate 13-acetate (PMA) and ionomycin with brefeldin A being added for the final 2.5 h of culture (all Sigma-Aldrich). The cells were fixed and permeabilized with Cytofix/Cytoperm (BD Biosciences for cytokines, eBioscience for transcription factors) according to the manufacturer’s instructions. The cells were stained with anti-cytokine antibodies (for antibody list, see Supplementary Table 2) and were analyzed with a BD LSRFortessa (BD Biosciences), a CytoFLEX (Beckman Coulter), a MACSQuant Analyzer (Miltenyi Biotec) or a Cytek Aurora Analyzer. Flow cytometry data were analyzed with FlowJo software (Tree Star) or Cytobank (Cytobank Inc.). Cytokines in culture supernatants were quantified by ELISA (R&D Systems) or Luminex (Thermo Fisher Scientific) according to standard protocols. High-dimensional cell surface phenotyping of sorted human memory CD8^+^ T cells from four individual healthy blood donors was performed with a newly established 33-color panel for surface markers (Supplementary Table 2) on a Cytek Aurora Analyzer. The cells were analyzed on day 5 after stimulation with CD3 and CD28 mAbs under high and low NaCl conditions.

For phospho-flow analysis, CD8^+^CD45RA^−^ T cells were collected on day 5 after stimulation with CD3 and CD28 mAbs and rested in FCS-free medium at 37 °C for 30 min. After incubation with anti-CD3 (clone TR66, EnzoLifeScience) for 10 min on ice, cells were stained with live/dead zombie dye (BioLegend). The cells were then washed and incubated with anti-mouse immunoglobulin (Ig)G F(ab′)_2_ fragment for the indicated time points. Immediately after F(ab′)_2_ incubation, cells were fixed in CytoFix buffer or Lyse/Fix buffer at 37 °C for 10 min, then permeabilized by pre-cooled BD Perm buffer (both from BD Biosciences) on ice for 30 min. The cells were then incubated with an Fc blocker (BioLegend) and stained for 30 min at room temperature. Samples were recorded on a CytoFLEX and analyzed with FlowJo (BD Biosciences).

For 2-NBDG uptake assay, cells were harvested and rested in glucose-free medium for 10 min at 37 °C before incubation in glucose-free medium containing 100 μM 2-NBDG (Invitrogen) for 2 h at 37 °C. After a live/dead staining, samples were recorded on a CytoFLEX and analyzed with FlowJo.

For the BODIPY FL C16 uptake assay, cells were harvested and washed with 20 μM fatty acid-free bovine serum albumin (BSA)–PBS and then incubated in fatty acid-free BSA–PBS containing 1 μM BODIPY FL C16 (Invitrogen) for 30 min at 37 °C. After a live/dead staining, samples were recorded on a CytoFLEX and analyzed using FlowJo.

### Ca^2+^ flux measurement

T cells were collected at day 5 after prestimulation with CD3 and CD28 mAbs under low and high NaCl conditions. A total of 5 × 10^6^ cells were resuspended in 500 μl of cell culture medium with 5 μg ml^−1^ of Indo-1 AM or 3 μM Fura Red AM (both from Life Technologies) and incubated in the dark at 37 °C for 30 min. The cells were washed, then incubated in 10 μg ml^−1^ of anti-CD3 (clone TR66, EnzoLifeScience) in imaging buffer (PBS with 0.5 mM Ca^2+^, 0.5 mM Mg^2+^ and 1 g l^−1^ of glucose) for 10 min on ice. Cells were then washed and kept on ice with 7-AAD (BD Biosciences) or Höchest (Sigma-Aldrich) until measurement. For each Ca^2+^ flux measurement, cells were diluted with 37 °C pre-warmed imaging buffer and analyzed with a BD FACSAria Fusion Flow cytometer (BD Biosciences) or CytoFlex Flow cytometer (Beckmann Coulter). After acquisition of the baseline fluorescence level, TCR crosslinking was induced by addition of 1.3 μg ml^−1^ of AffiniPure F(ab′)_2_ Fragment Goat anti-Mouse IgG (Jackson ImmunoResearch). The calcium flux ratio was determined by the Indo-1 ratio between 400-nm (Ca^2+^ bound) and 475-nm (Ca^2+^ free) readings, or the Fura Red ratio between violet laser (Ca^2+^ bound) and green laser (Ca^2+^ free) readings. The calcium flux ratio was calculated using FlowJo.

### Metabolic assays

The mitochondrial function (oxidative phosphorylation) and glycolytic rate of CD8^+^CD45RA^−^ T cells, which were prestimulated for 5 d with CD3 and CD28 mAbs under high and low NaCl conditions, were assessed after washing using a Seahorse XFp Analyzer (Agilent Technologies). T cells were resuspended in Seahorse assay medium (pH adjusted to 7.40–7.45) on XF96 cell culture microplates at a density of 2.5 × 10^5^ cells per well. Cells were centrifuged for 5 min at 400*g* to adhere and form a monolayer at the bottom of the plate. All experiments were performed with four technical replicates. The XF^e^96 extracellular flux assay kit (Agilent Technologies) was used according to the manufacturer’s protocol with the addition of oligomycin, CCCP (carbonyl cyanide *m*-chlorophenyl hydrazone), antimycin A and rotenone at concentrations of 2 × 10^−6^ M, 1.5 × 10^−6^ M, 2 × 10^−6^ M and 2 × 10^−6^ M, respectively. Evaluation and calculation of mitochondrial and glycolytic indices were done with the Wave software 2.2.0 (Agilent Technologies). Glut-1 expression by T cells was analyzed by flow cytometry using anti-hGlut1-PE (R&D, cat. no. FAB1418P) or anti-hGlut-1-FITC (R&D, cat. no. FAB1418F). Fatty acid uptake was analyzed by flow cytometry using BODIPY FL C16 (Invitrogen, 1 μM, excitation/emission 505/512 nm on FITC channel). For ATP quantification, the CellTiter-Glo Luminescent Cell Viability Assay (Promega, cat. no. G7570) was used. The luminescent signal was measured using CLARIOstar (BMG Labtech) and normalized to the absolute cell number. For quantification of the Na^+^/K^+^-ATPase activity, a colorimetric ATPase Assay Kit (Abcam) was used. After extraction of ATPase by a standard protocol, it was incubated with phosphate substrate in the presence or absence of 10 μM ouabain (Sigma-Aldrich) at 25 °C for 30 min. Results were recorded at 620 nm using a SPARK reader (TECAN).

Nontargeted metabolic profiling of CD8^+^ T cells after stimulation with CD3 and CD28 mAbs for 5 d in high and low NaCl conditions was performed by LC–tandem MS (LC–MS/MS) at Metabolon according to standard procedures. Raw data were extracted, peak identified and quality control (QC) processed using Metabolon’s hardware and software. After normalization to cell count, log(transformation) and imputation of missing values, with the minimum observed value for each compound, analysis of variance (ANOVA) contrasts were used to identify biochemicals that differed significantly between experimental groups. Data were imported to Graphpad Prism (v.7-9) for visualization.

### Gene expression analysis

For messenger RNA-seq analyses (bulk transcriptome), CD8^+^ memory T cells were isolated ex vivo as described above and stimulated for 48 h with CD3 and CD28 mAbs for a total culture time of 5 d in the presence of low and high NaCl concentrations (Gene Expression Omnibus (GEO) accession no. GSE232365). Total RNA was extracted from cells lysed in TRI reagent (Sigma-Aldrich) according to the manufacturer’s protocol. RNA was quantified using a NanoDrop 2000 spectrophotometer (Thermo Fisher Scientific) and its quality was verified by an Agilent 2100 Bioanalyzer according to the manufacturer’s guidelines.

Library preparation for RNA-seq was performed using the TruSeq Stranded Total RNA Sample Preparation Guide (Illumina). The barcoded libraries were sequenced on a NovaSeq 6000 platform (Illumina) by Novogene with paired-end, 150-bp reads (PE150). Approximately 2 Gb of sequencing reads were produced on average per sample. The reads were mapped to the reference transcriptome built from the human genome assembly hg38 (GRCh38) using STAR v.2.6.1a (ref. ^63^). Transcripts were quantified with salmon v.0.11.3 in the alignment-based mode^64^.

Significant DEGs were identified using the R package DESeq2 (ref. ^65^), using a false discovery rate (FDR)-corrected significance threshold of 0.05. Of 60,623 tested genes, we identified 26,837 with a nonzero mean expression. Of these, 1,956 were significantly up- and 1,926 significantly downregulated. Plots were produced with the R package ggplot2 (ref. ^66^). The R package clusterProfiler v.4.6.0 (ref. ^23^) was employed for overrepresentation analysis, as well as GSEA and visualization of GO terms within the ontology ‘Biological Process’ and KEGG pathways. The enrichment plots show the top 20 entries for the significantly upregulated, downregulated and dysregulated genes, respectively, which were ranked according to the shrunken fold-change values calculated by DESeq2, as previously described^65^. Barcode plots of enriched gene signatures were generated with clusterProfiler and the package fgsea (v.1.25.2) for computing a normalized enrichment score (NES) and statistics based on an adaptive multilevel split Monte Carlo scheme. Heatmaps were generated using the R package ComplexHeatmap v.2.14.0 (ref. ^67^). Overlay of expression data over KEGG pathways was done with the R package pathview v.1.38.0 (ref. ^68^). Real-time quantitative PCR (RT–qPCR) analysis was performed using Taqman probes targeting *NFAT5* and 18S with a CFX Real-Time PCR instrument (BioRad).

### Transcriptomic profiling of human cancers from TCGA

Gene expression data of patients with cancer were obtained from TCGA. Nondiseased, tissue-specific expression data were collected from the GTEx project. Gene expression data from both projects were re-preprocessed by the same RNA-seq pipeline from the University of California, Santa Cruz (UCSC) RNA-seq Compendium^69^. The resulting gene expression data were made publicly available as a TCGA–TARGET–GTEx cohort (https://xenabrowser.net/datapages/?cohort=TCGA%20TARGET%20GTEx), which was used for subsequent transcriptomic profiling of human cancers. Data from the TARGET cohort (pediatric data: https://www.cancer.gov/ccg/research/genome-sequencing/target) was discarded. We downloaded transcripts per kilobase million (TPM)-normalized RNA-seq-based expression data for 60,498 genes from the Xena platform as well as associated metadata (https://toil.xenahubs.net/download/TcgaTargetGtex_rsem_gene_tpm.gz, https://toil.xenahubs.net/download/TcgaTargetGTEX_phenotype.txt.gz, downloaded 6 March 2023)^1^. By using data from the toil hub, it was ensured that data were recomputed with one pipeline. After filtering for tumor entities that included healthy control tissues, 8,972 ‘primary tumor’ samples and 727 ‘solid tissue normal’ samples of 9,019 patients (TCGA dataset) and 4,472 ‘normal tissue’ samples of 535 donors (GTEx dataset) across 25 tumor types were available. Expression data for healthy samples (‘solid tissue normal’, ‘normal tissue’) were pooled per tumor site. To enable GSEA, log_2_(fold-changes) of gene expression data were calculated per gene and tumor sites between mean TPM-expression data of tumor and healthy samples. Subsequent GSEA using all 1,956 upregulated genes of the salt signature and the log_2_(fold-changes)-ranked gene list per tumor site as input was performed using the R fgsea package to compute NES and one-tailed test-based *P* values for positive enrichment of the gene signature. Default parameters were used for calculating GSEA with the function fgsea::fgsea except for minSize (=10) and maxSize (=4000) and scoreType (=‘pos’).

For Kaplan–Meier test statistics, TCGA-based expression data of *NFAT5* (normalized to TPM) was filtered from the Xena platform^69^. The R packages survival (v.3.5-5) and survminer (v.0.4.9) were used to compute and visualize Kaplan–Meier survival statistics and significance with the Peto–Peto algorithm, respectively. TCGA data for breast and pancreas cancer were filtered for the attribute sample_type = ‘primary tumor’; 81 samples (from 81 patients) were obtained for pancreatic adenocarcinoma and 113 samples (from 113 patients) for breast cancer with information on time (attribute ‘new_tumor_event_dx_days_to’) and expression of the gene of interest. Each TCGA cancer patient dataset was subsequently subdivided into high and low gene expression groups by using the function surv_cutpoint of the survminer R package with default values.

### Single-cell mRNA-seq analysis

For scRNA-seq analysis of matched tumoral and peritumoral CD8^+^ T cells from patients with breast cancer, tissue samples from three patients with breast cancer (BC01, BC02 and BC03) were reanalyzed (GEO accession no. GSE114727)^11^. QC with removal of cells expressing <200 genes and of genes present in <3 cells was performed using the python package Scanpy v.1.9.1. Doublets were removed with Scrublet v.0.2.3. Data were normalized to 10,000 reads per cell using normalize_per_cell() and log(transformed) using log1p() functions. Sample integration and batch correction were performed using the combat() function provided by Scanpy. CD8^+^ T cells were annotated with CellTypist v.1.3.0 using the pretrained model Immune_All_High.pkl. Cells assigned to a probability of being a T cell <0.6 were filtered out. Then, T cells with expression values of CD8A > 2.5 and CD8B > 2 were identified as CD8^+^ T cells. Transcriptomic NaCl signatures were derived from either a bulk transcriptomic comparison of FACS-sorted human CD8^+^CD45RA^−^ T cells stimulated with CD3 and CD28 mAbs for 5 d under high and low NaCl conditions (FDR < 0.05) or scRNA-seq of human CD8^+^CD45RA^−^ T cells stimulated with CD3 and CD28 mAbs for 3 d under high and low NaCl conditions (*P*_adj_ < 0.05). Significantly up- and downregulated gene sets from the two transcriptomic analyses were tested separately on the tumoral and peritumoral CD8^+^ T cells from the scRNA-seq analysis of the three patients with breast cancer. For GSEA, genes were ranked according to their expression values using the rank_genes_groups() function provided by Scanpy and analyzed with gseapy v.1.0.2. Module scores were calculated with the Scanpy score_genes() function. For comparisons of tumoral versus peritumoral CD8^+^ T cells, statistical significance was determined using Wilcoxon’s rank-sum test with the alternative hypothesis = ‘greater’ for upregulated gene sets and ‘less’ for downregulated gene sets.

For scRNA-seq (GEO accession no. GSE232149), a library of human CD8^+^CD45RA^−^CD45RO^+^ cells and human CD8^+^CD45RA^+^CD45RO^−^ cells from one donor that were stimulated with CD3 and CD28 mAbs for 3 d under high and low NaCl conditions was constructed with Chromium Next GEM Single Cell 5′ Reagents v.2 (Dual Index; 10x Genomics, Inc.). The library was sequenced on an Illumina NovaSeq 6000 Sequencing System (Flow Cell Type S4) according to the manufacturer’s instructions, with 150-bp, paired-end, dual-indexing sequencing (sequencing depth: 20,000 read pairs per cell). Read alignment and gene counting of the single-cell datasets were performed with CellRanger v.7.0.1 (10x Genomics, Inc.). For downstream analysis, the filtered barcode matrix (CellRanger multipipeline) was processed with the R package Seurat v.4.0.4. For QC, cells with unique feature counts >9,000 and a count value >80,000 were filtered out. The total counts were normalized to 10,000 reads per cell. Each gene was centered and scaled to unit variance. Doublets were removed using the R package DoubletFinder v.2.0.3. UMAPs were generated using the RunUMAP() function and Leiden clustering was performed using the FindNeighbors() and FindClusters() functions from Seurat. The top ten marker genes per Leiden cluster were determined with the wilcoxauc() function in the R package presto (v.1.0.0) and depicted in a heatmap using the R package ComplexHeatmap (v.2.12.1). Bray–Curtis dissimilarity was calculated using the vegan R package (v.2.6.4). Differential gene expression was evaluated using FindMarkers() and genes were declared as significant based on an adjusted *P*-value threshold of 0.05. GSEA for GO was conducted using clusterProfiler (v.4.4.4). Multiple testing correction was performed using the Benjamini–Hochberg method and significantly enriched GO terms were visualized in a dot plot with clusterProfiler (v.4.4.4).

In analyses represented by violin plots, the python package Scanpy v.1.9.1 was used as an alternative to the R package Seurat v.4.0.4. Doublets were predicted and removed using Scrublet v.0.2.3 as before. Cells expressing <200 genes were filtered out. Cells were normalized to 10,000 reads per cell using normalize_per_cell() and log(transformed) using log1p() functions. Gene expression values were scaled to a maximum value of 10. For UMAP, highly variable genes were computed using the function scanpy.pp.highly___variable___genes(). PCA was performed using the function pca() *n* = 30. Finally, the neighbors of each cell and UMAP were computed using the functions scanpy.pp.neighbors() and scanpy.pp.umap(), respectively. Module scores were computed using the function score_genes() provided in the python package Scanpy v.1.9.1.

For trajectory analyses, diffusion pseudotime was used^70^. The cell with the highest expression of the stemness marker gene *TCF7* was chosen as the starting point for pseudotime inference. For validation, the analysis was repeated using the cell with the highest expression of the stemness gene set from a public dataset^71^. The function diffmap() and dpt() from Scanpy were used to compute the diffusion map representation and to assign pseudotime values to each cell in the dataset. For RNA velocity, the velocyto pipeline v.0.17.17 was used to obtain the pre-mature (unspliced) and mature (spliced) transcript information based on CellRanger output. The functions scv.pp.moments(), scv.tl.velocity() and scv.tl.velocity_graph() from scVelo v.0.2.5 were applied to recover RNA velocity and scv.pl.velocity_embedding_stream() was used for visualization. QC and preprocessing were performed using the python package Scanpy v.1.9.1. Genes with a minimum count of 1 were retained and doublets were removed using Scrublet v.0.2.3. Total count normalization was applied using normalize_total(). Data were log(transformed) using log1p() and highly variable genes were identified using highly_variable_genes().

### ICP–OES

For sample preparation, frozen specimens from patients with breast cancer (age range 38–86 years) were subject to a freeze dryer (Heraeus Christ) and lyophilized at a −35 °C condenser temperature until the weight became constant. Subsequently, the dried samples were transferred into closed quartz vessels and digested with HNO_3_ (suprapure, sub-boiling distilled) in a Discover SP-D 80 microwave digestion system (CEM Corp.). The resulting solution was brought to exactly 10 ml with Milli-Q H_2_O and was then ready for element determination.

Element determination of Na and K was performed with ICP–OES ARCOS (Ametek-Spectro). The measured spectral element lines in nanometers were K 766.491 nm and Na 589.592 nm. Sample introduction was carried out by a peristaltic pump, connected to a MicroMist nebulizer with a cyclone spray chamber. The radiofrequency power was set to 1,400 W, the plasma gas was 15 l Ar per min and the nebulizer gas was 0.6 l Ar per min.

For QC, three blank determinations and a control determination of a certified standard (CPI) for all mentioned elements were performed regularly after ten measurements. Analysis of the results was carried out on a computerized laboratory data management system, which related the sample measurements to calibration curves, blank determinations and control standards.

### Transmission electron microscopy

Cell pellets were fixed with Karnovsky fixative. After secondary fixation with 2% osmium tetroxide (Chemex) and 1% potassium hexacyanidoferrate (II) (Merck), serial dehydration steps were performed with acetone (Roth). Staining was performed with 1% uranyl acetate (Merck) in 50% acetone. Sample infiltration with Epon (Serva Electrophoresis) was performed with a mixture of acetone:Epon (3:1, 1:1, 1:3), followed by pure Epon and Epon with accelerator (BDMA, Agar Scientific). Samples were polymerized at 60 °C for 48 h. Trimming was performed with a Leica EM Rapid. Sections were made with an ultramicrotome UC7 (Leica). Semi-thin sections of 0.5 µm were dyed with Azure (Azure II, Sigma-Aldrich) and borax solution 5% (Sigma-Aldrich). Ultrathin sections of 55 nm were placed on copper slot grids coated with a Formvar/Carbon layer. The images were taken using a transmission electron microscope JEM 1400 (JEOL) with an acceleration voltage of 80 kV and the CCD camera ‘Orius SC 1000 A’ (GATAN) using the software GATAN Microscopy Suite (v.2.31.734.0). Images were analyzed using ImageJ2 (v.6).

### Mouse experiments

Congenic C57BL/6 CD45.2^−^CD45.1^+^ mice and OT-1 (B6.Cg-Tg(TcraTcrb)1100Mjb) mice were bred in-house (Animal Facility Philipps-University Marburg, BMFZ). OT-1 (B6.Cg-Tg(TcraTcrb)1100Mjb) mice were obtained from Jackson Laboratories. CD8^+^ T cells were obtained from the lymph nodes and spleens of OT-1 mice using a negative selection kit (Miltenyi Biotec, cat. no. 130-104-075). For CTL differentiation, naive CD8^+^ T cells were cultured in RPMI (10% FCS) and stimulated with plate-bound mCD3 mAbs (3 µg ml^−1^, clone 145-2C11, BioLegend), soluble mCD28 (0.5 µg ml^-1^, clone 37.51, BioLegend), recombinant human IL-2 (50 U ml^−1^, Novartis) and anti-mIFN-γ (5 µg ml^-1^, clone XMG1.2, BioLegend) in the presence or absence of 30 mM NaCl. For intracellular cytokine staining, cells were restimulated after 72 h of culture with PMA (50 ng ml^−1^) and ionomycin (1 µg ml^−1^, both from Sigma-Aldrich) in the presence of brefeldin A (5 µg ml^−1^; BioLegend) for 4 h. Cells were fixed with 2% formaldehyde. Intracellular staining for TNF–FITC (eBioscience), granzyme B–PE (eBioscience) and IFN-γ–APC (BioLegend) was performed in saponin buffer (0.1% saponin and 1% BSA in PBS). For proliferation, CD8^+^ T cells were labeled with the cell proliferation dye eFluor 670 (2 µM). The cells were then washed, resuspended in culture medium and seeded into a 96-well-plate. Proliferation was measured by flow cytometry on day 3. Apoptosis was quantified using annexin V–PI staining. Cells were stained for 20 min in balanced salt solution with annexin V (APC, cat. no. 640920, BioLegend) at room temperature. PI (Invitrogen) was added immediately before flow cytometric analysis on an Attune NxT Cytometer (Thermo Fisher Scientific).

For in vivo animal experiments, 8- to 12-week-old CD45.1 mice were injected subcutaneously (s.c.) with 1 × 10^6^ Panc^OVA^ cells. Then 7 d post-injection, tumor-bearing animals were treated with 0.5 × 10^6^ CTLs, which had been differentiated from naive OT-1 CD8^+^ T cells for 3 d in vitro in the absence or presence of additional 30 mM NaCl. Tumor growth was measured and tumor volume was calculated (*V* = length × width^2^ × 0.5 mm^3^). For the ex vivo characterization of tumor-infiltrating T cells, tumors were isolated 3 d after adoptive T cell transfer and were digested with 200 U ml^−1^ of collagenase IV (Worthington, cat. no. LS004188), 10 μg ml^-1^ of DNase 1 (Roche, cat.no. 4536282001) in Hank’s balanced salt solution, 37 °C and 300 r.p.m. agitation and filtered through 100-μm cell strainers (Sysmex). Tumor lysates were stained with live/dead Zombie NIR dye (BioLegend, cat. no. 423106). Surface staining was performed using CD45.2 (BV510, BioLegend, cat. no. 109837), CD8 (PerCP/Cy5.5, BioLegend, cat. no. 100734), PD-1 (PE, eBioscience, cat. no. 12-9985-82) and TIM3 (APC, BioLegend, cat. no. 119706). For antigen-specific intracellular cytokine stimulation, tumor lysates were incubated for 4 h with OVA_257–264_ (JPT, cat. no. 43194) at 37 °C in RPMI with 5 µg ml^−1^ of brefeldin A. Intracellular staining was performed as described above.

Adherent Panc^OVA^ cells were grown in T75 flasks (Sarstedt) with Dulbecco’s modified Eagle’s medium (10% FCS) and split every 2–4 d on reaching 70% confluence, harvested by trypsinization (1× trypsin/EDTA, Sigma-Adrich, cat. no. T-4174) and reseeded (5 × 10^5^ cells per 10 ml of medium in a T75 flask). For selection, 500 mg l^-1^ of G418 (Sigma-Aldrich, cat. no. G8168) was added.

### CAR T cell generation

For CAR T cell manufacturing, CD8^+^ T cells were isolated with >90% purity from the spleens and lymph nodes of 8-week-old C57/BL6 mice by positive selection using magnetic microbeads (Miltenyi Biotec) and stimulated using plate-bound anti-CD3 (5 µg ml^−1^, clone 145-2C11) and soluble anti-CD28 mAbs (1 µg ml^−1^, clone 37.51) for 72 h. Then, 24 h after activation, CD8^+^ T cells were transduced with a second-generation ROR1 (tyrosine kinase-like orphan receptor 1) CAR using retroviral supernatant supplemented with polybrene, as described previously^72^. CAR T cells were expanded under high or low NaCl conditions for 48 h.

To assess the cytotoxicity of the anti-ROR1 CAR T cells, a coculture with firefly luciferase-expressing ROR1^+^ Panc02 target cells was performed. After washing with RPMI, CAR T cells and Panc02-ROR1 cells were plated at an E:T ratio of 10:1 and 2.5:1 (effector CAR T cell:target cell) in white, 96-well, flat-bottomed plates with medium containing 150 µg ml^−1^ of d-luciferin substrate. The chemiluminescence signal was measured at different time points at 37 °C using the Tecan plate reader Infinite 200. Lysis mediated by CAR T cells was calculated as the reduction of signal by effector cells compared with mock-transfected T cells: Specific lysis (%) = Mean((lysis by mock cells) − Single value (lysis by CAR T cells)/Mean(lysis by mock cells)) × 100. EpCAM-specific CAR T cells were generated using a second-generation CAR construct and functionally assessed according to a similar procedure.

### Statistics

Statistical tests are indicated in the corresponding figure legends. Statistical tests for transcriptomic or metabolomic analyses are described in the corresponding sections. Error bars indicate the s.e.m. unless otherwise stated; *P* values ≤ 0.05 were considered significant; *n* indicates the number of biological replicates and individual blood donors. Analyses were performed using GraphPad Prism v.7-10. Data collection and analysis were not performed blind to the conditions of the experiments. For animal experiments, no statistical methods were used to predetermine sample sizes. Tumor-bearing mice were distributed equally between the groups according to their tumor size.

### Study approval

Ethical approval was obtained from the institutional review boards of the Technical University of Munich (195/15s, 146/17s, 491/16s), Charité-Universitätsmedizin Berlin (EA1/221/11) and Friedrich Schiller University Jena (2020-1984_1). All blood donors provided their informed consent. All work was carried out in accordance with the Declaration of Helsinki for experiments involving humans and with the Regierungspräsidium Giessen for studies involving mice. The maximal tumor size of 1.5 cm, as approved by the Regierungspräsidium Giessen, was not exceeded.

### Immunoblot analysis

Nuclear and cytosolic extracts from CD8^+^CD45RA^−^ T cells after stimulation with CD3 and CD28 mAbs were separated using the Nuclear Extraction kit (Abcam). Protein concentrations were quantified using the Pierce BCA Protein Assay Kit (Thermo Fisher Scientific). Protein, 25 mg per sample, was boiled with 4× Laemmli sample buffer (BioRad Laboratories) containing 355 mM 2-mercaptoethanol (BioRad Laboratories) at 96 °C for 10 min and loaded on to a 8% sodium dodecylsulfate–polyacrylamide gel electrophoresis gel. The following antibodies were used: mouse anti-human NFAT5 antibody (Santa Cruz, cat. no. sc-398171), rabbit anti-human Lamin-B1 antibody (Cell Signaling Technology, cat. no. D4Q4Z), mouse anti-human β-actin (Cell Signaling Technology, cat. no. 8H10D10) antibody. Horseradish peroxidase-conjugated anti-mouse and anti-rabbit IgG antibodies (Cell Signaling Technology) were used as secondary antibodies. The immunoreactive bands were detected using SuperSignal West Femto Maximum Sensitivity Substrate (Thermo Fisher Scientific). The chemiluminescence signals were recorded and analyzed using the iBright analysis system (Thermo Fisher Scientific).

### Stable isotope tracing with metabolite extraction and GC–MS measurement

CD8^+^CD45RA^−^ T cells were stimulated with plate-bound CD3 and CD28 mAbs for 48 h and cultured in complete medium until day 4 under high and low NaCl conditions as described above. The medium was replaced on day 4 with tracer medium containing 2 mM [U-^13^C-5]glutamine (Cambridge Isotope Laboratories) and all supplements were present in complete medium except for normal l-glutamine. Cells were then incubated for a further 24 h to reach isotopic steady state before starting metabolite extraction.

For metabolite extraction and the gas chromatography (GC)–MS measurement, the cells were washed with 0.9% NaCl. They were then quenched with ice-cold methanol and ddH_2_O (with 1 μg ml^−1^ of d-6-glutaric acid as an internal standard). Scraped cell extracts were combined with ice-cold chloroform, vortexed at 1,400 r.p.m. for 20 min at 4 °C, and then centrifuged at 17,000*g* for 5 min at 4 °C to achieve phase separation. Subsequently, 250 μl of the upper polar phase was transferred to GC glass vials with microinserts and subjected to cold vacuum drying. GC–MS measurement of isotope enrichment was performed. Dried extracts were derivatized at 55 °C with equal amounts of methoxylamine (20 mg ml^−1^ in pyridine) and MTBSTFA and injected into the GC–MS system. The separation of metabolites was accomplished using an Agilent 7890B gas chromatograph equipped with a 30 m × 25 mm × 0.25 µm ZB-35 ms (Phenomenex) and 5-m Guard column (Agilent). A sample volume of 1 µl was injected into the GC inlet at 270 °C using helium as the carrier gas at a flow rate of 1 ml min^−1^. The temperature of the GC oven was initially maintained at 100 °C for 2 min and then gradually increased by 10 °C per min until it reached 300 °C, where it was maintained for a further 4 min. The electron ionization energy was set to 70 eV. The temperatures of the MS source and the quadrupole were set to 230 °C and 150 °C, respectively. The detection of metabolites in selected ion mode was performed with an Agilent 5977 MSD system. The chromatogram analysis and the calculation of mass isotopomer distributions were performed with the Metabolite Detector Software^73^.

### PPI analyses

To construct a PPI network for the mTOR pathway, curated PPI information was retrieved from BioGRID (v.4.4.230: https://thebiogrid.org) with the keyword ‘MTOR’ or ‘SGK1’. The resulting lists were put together and cleaned for duplicates. The R package igraph (v.1.4.2) was used to construct the interaction network. Node centrality was assessed by computing the node degree and visualized by adjusting node size. The log_2_(fold-change) of the bulk RNA-seq data (high salt versus low salt) was used to color the nodes. Edge colors were computed by Pearson’s correlation of CD8 bulk RNA-seq samples. Toward this aim, we computed first a log_2_(fold-change) per gene and RNA-seq replicate for high versus low NaCl, yielding three high NaCl-sensitive values per gene. Subsequently, the high versus low NaCl log_2_(fold-changes) were used to compute Pearson’s correlation for every gene–gene pair for which we found curated PPI information. In addition, STRING was used to construct functional PPI networks.

### Reporting summary

Further information on research design is available in the Nature Portfolio Reporting Summary linked to this article.

## Online content

Any methods, additional references, Nature Portfolio reporting summaries, source data, extended data, supplementary information, acknowledgements, peer review information; details of author contributions and competing interests; and statements of data and code availability are available at 10.1038/s41590-024-01918-6.

## Supplementary information

Supplementary Information Supplementary Figs. 1–24 with legends and Methods.

Reporting Summary

Supplementary Data 1 Statistical information on all figures containing box plots.

Supplementary Table 1.

Supplementary Table 2.

Supplementary Table 3.

## Data Availability

ScRNA-seq and bulk mRNA-seq data of CD8^+^ T cells stimulated in vitro and high and low NaCl conditions have been deposited in the National Center for Biotechnology Information GEO under accession nos. GSE232149 and GSE232365. Accession no. GSE114727 was accessed for analysis of T cells in patients with breast cancer. Accession nos. GSE155698, GSE111672, GSE154778, GSM4293555 and PRJCA001063 were accessed for analysis of T cells in patients with pancreatic cancer (10.5281/zenodo.6024273).

## Code availability

The code used for the analysis in this study is available on GitHub at https://github.com/mBiocoder/Nature_immunology_code_repository/tree/main and has been archived in *Zenodo* at https://zenodo.org/record/12201092 (ref. ^74^).
