# Supplementary material for: Sodium chloride in the tumor microenvironment enhances T cell metabolic fitness and cytotoxicity
Source: Nat Immunol. 2024 Aug 28;25(10):1830–44. doi: 10.1038/s41590-024-01918-6 (PMC11436365; doi:10.1038/s41590-024-01918-6)
Supplement: Supplementary file 1 — Supplementary Figs. 1–24 with legends and Methods. [file 41590_2024_1918_MOESM1_ESM.pdf]

nature immunology

Supplementary information https://doi.org/10.1038/s41590-024-01918-6

Sodium chloride in the tumor microenvironment enhances T cell metabolic fitness and cytotoxicity

In the format provided by the authors and unedited

Supplementary Figure 1

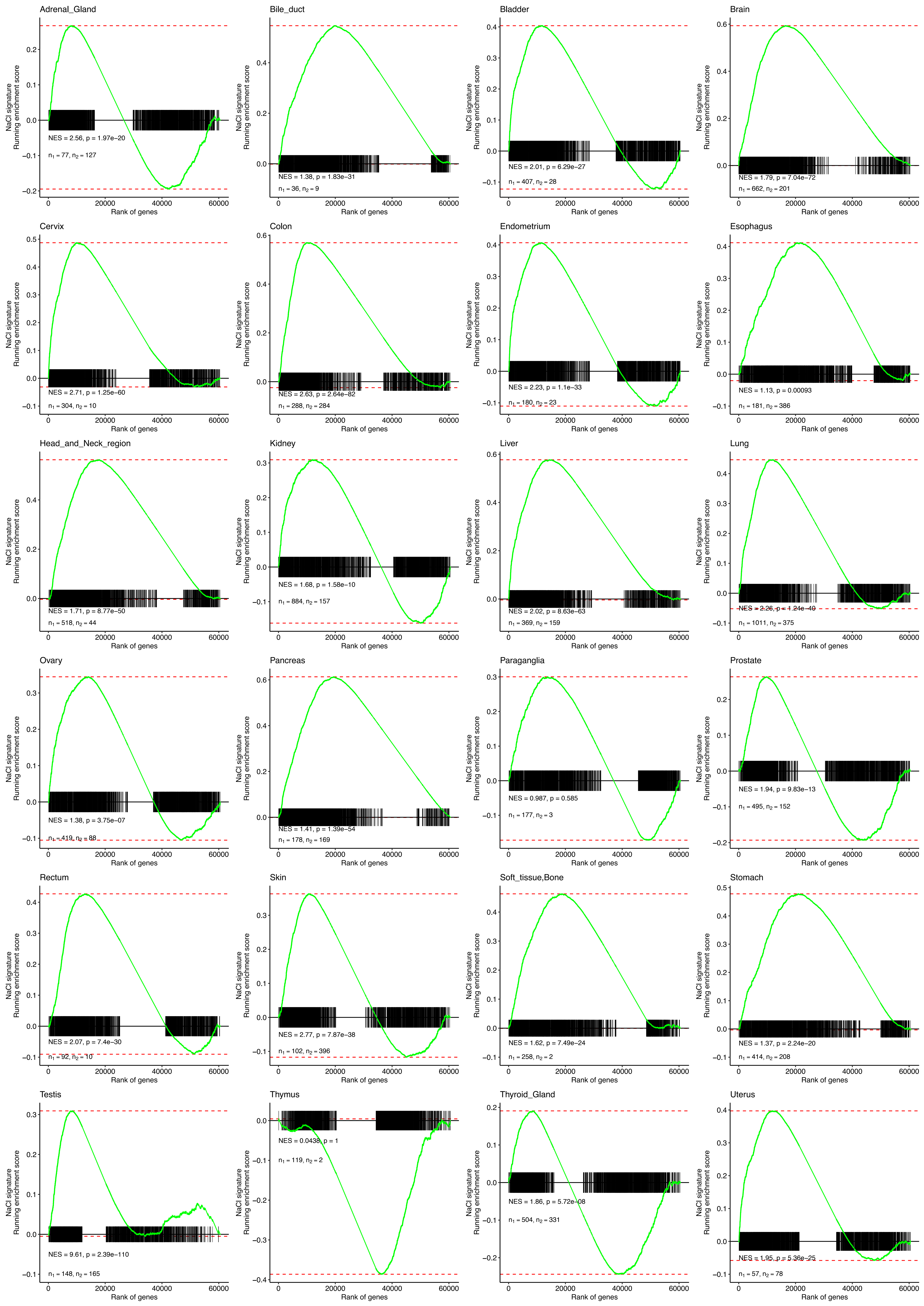

Supplementary Figure 2

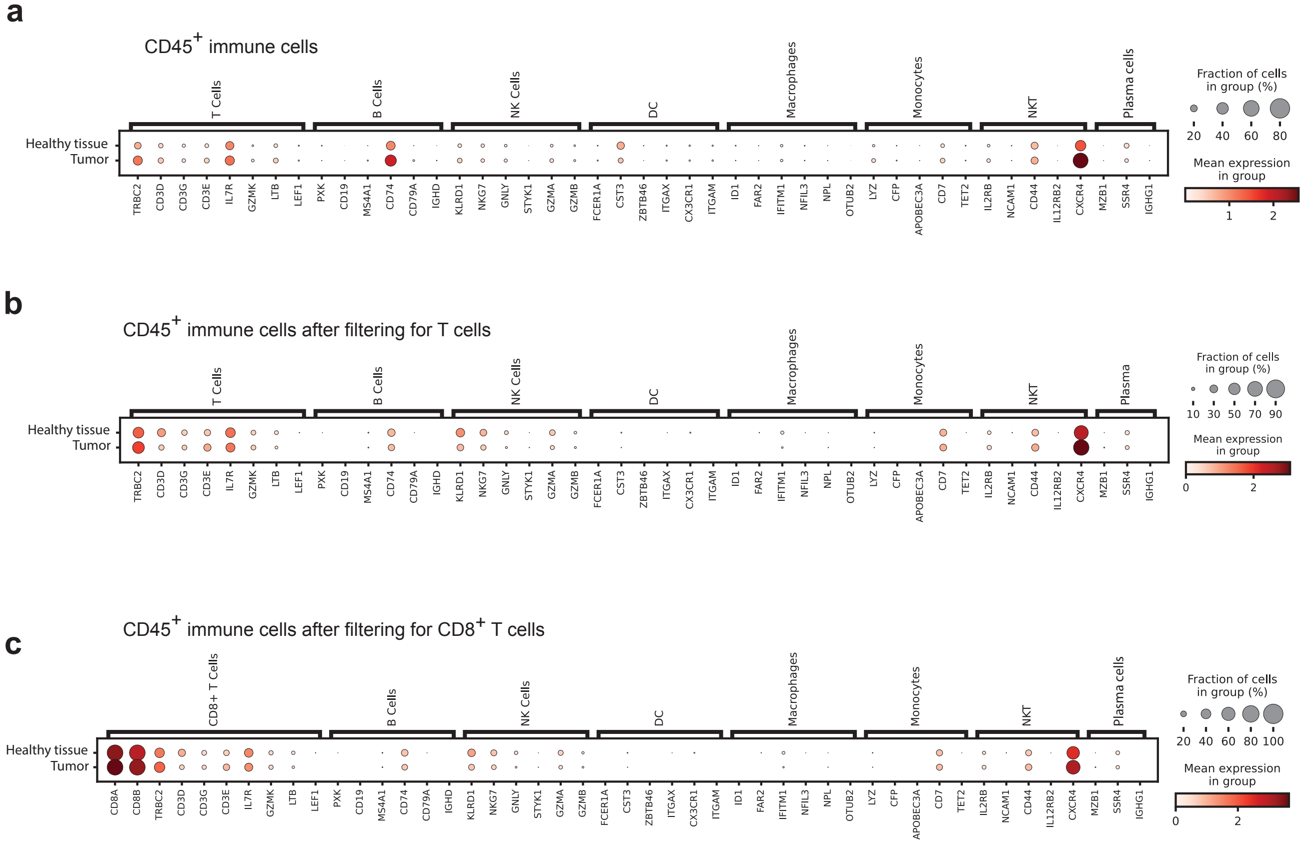

Supplementary Figure 3

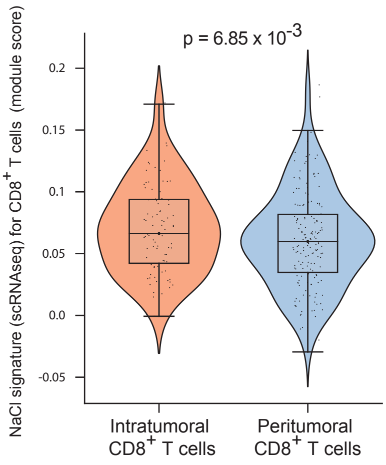

Supplementary Figure 4

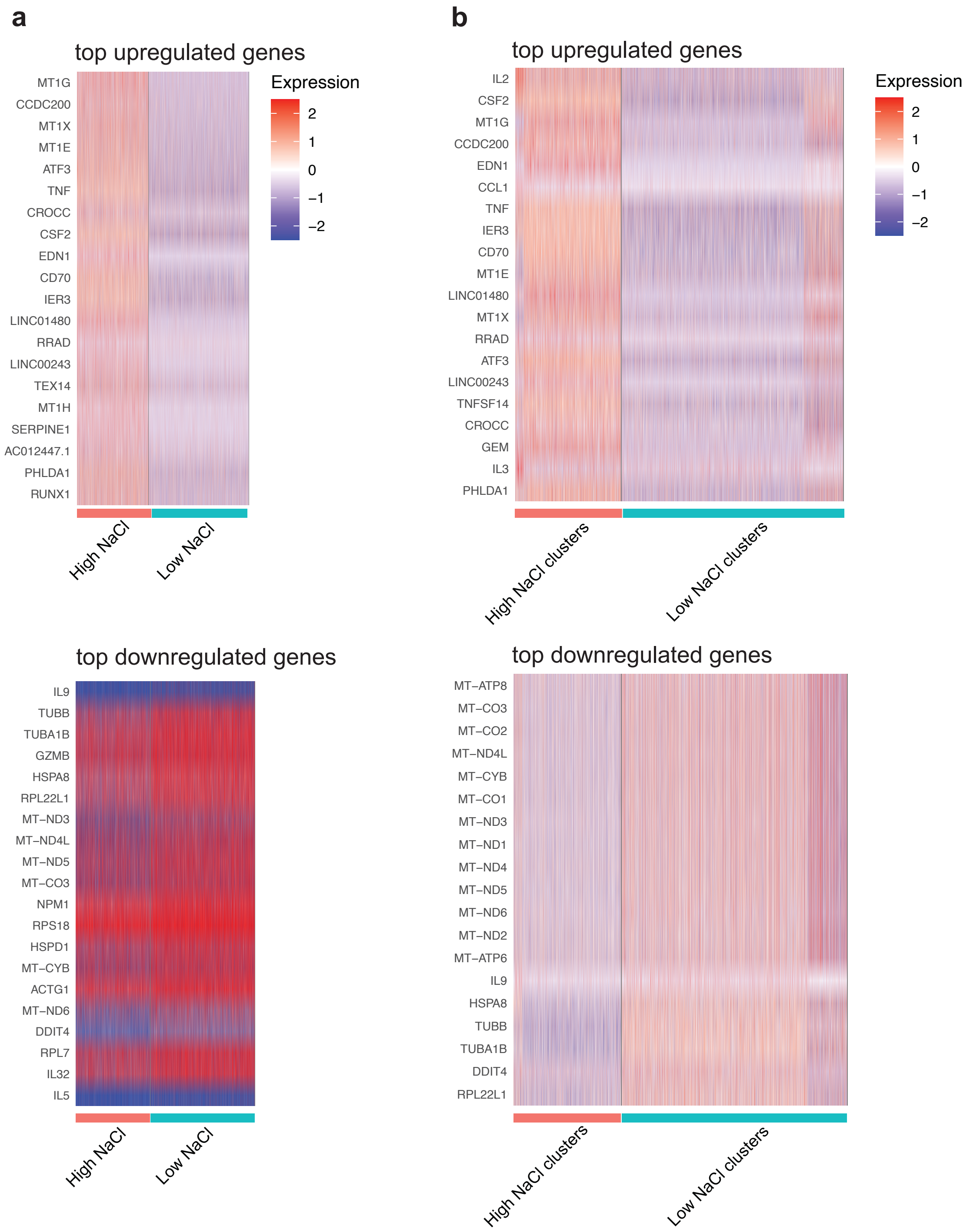

Supplementary Figure 5

a Gating strategy

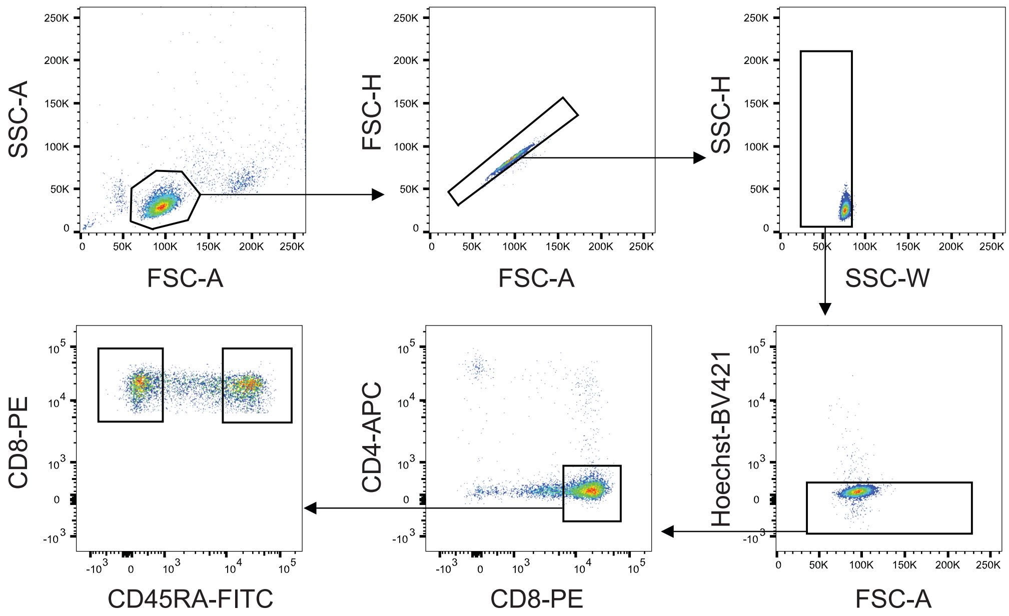

b

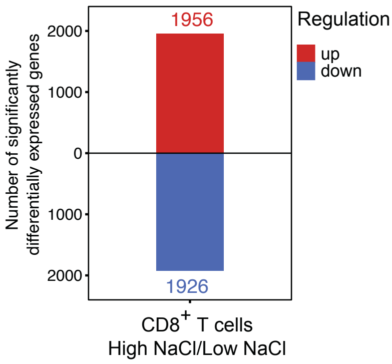

c

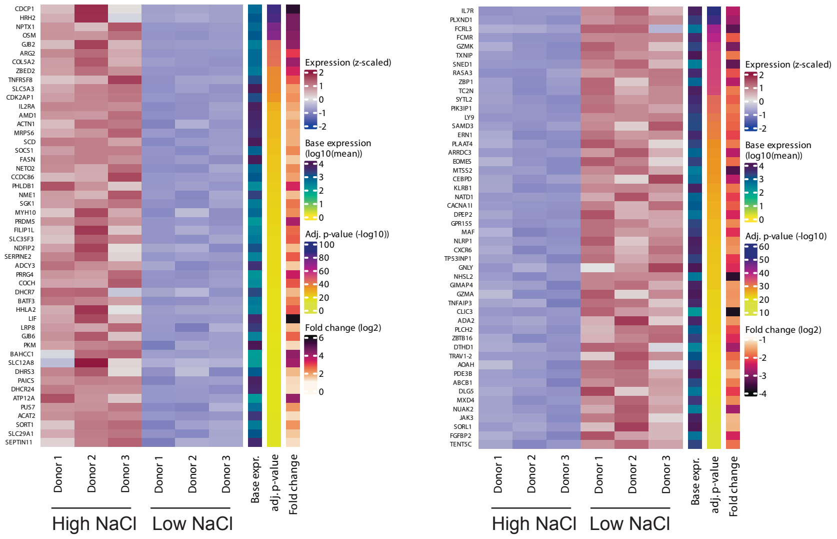

Supplementary Figure 6

a

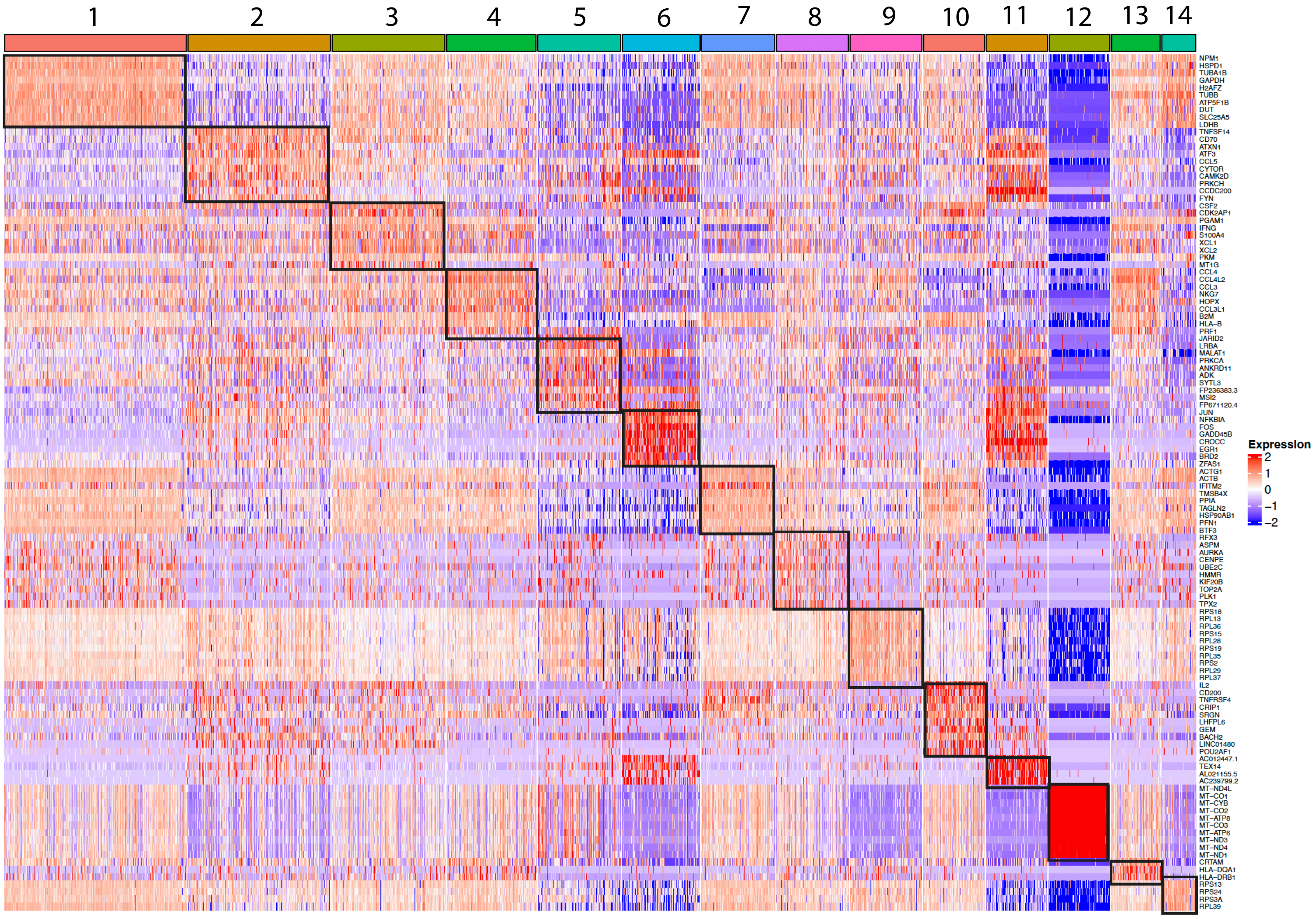

b

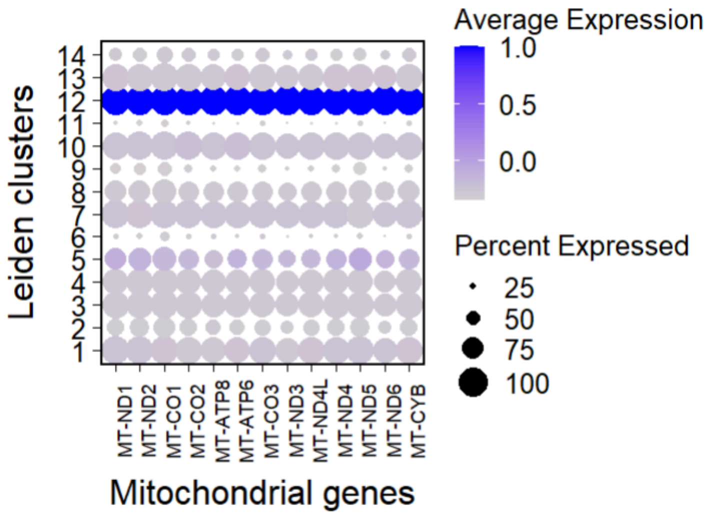

Supplementary Figure 7

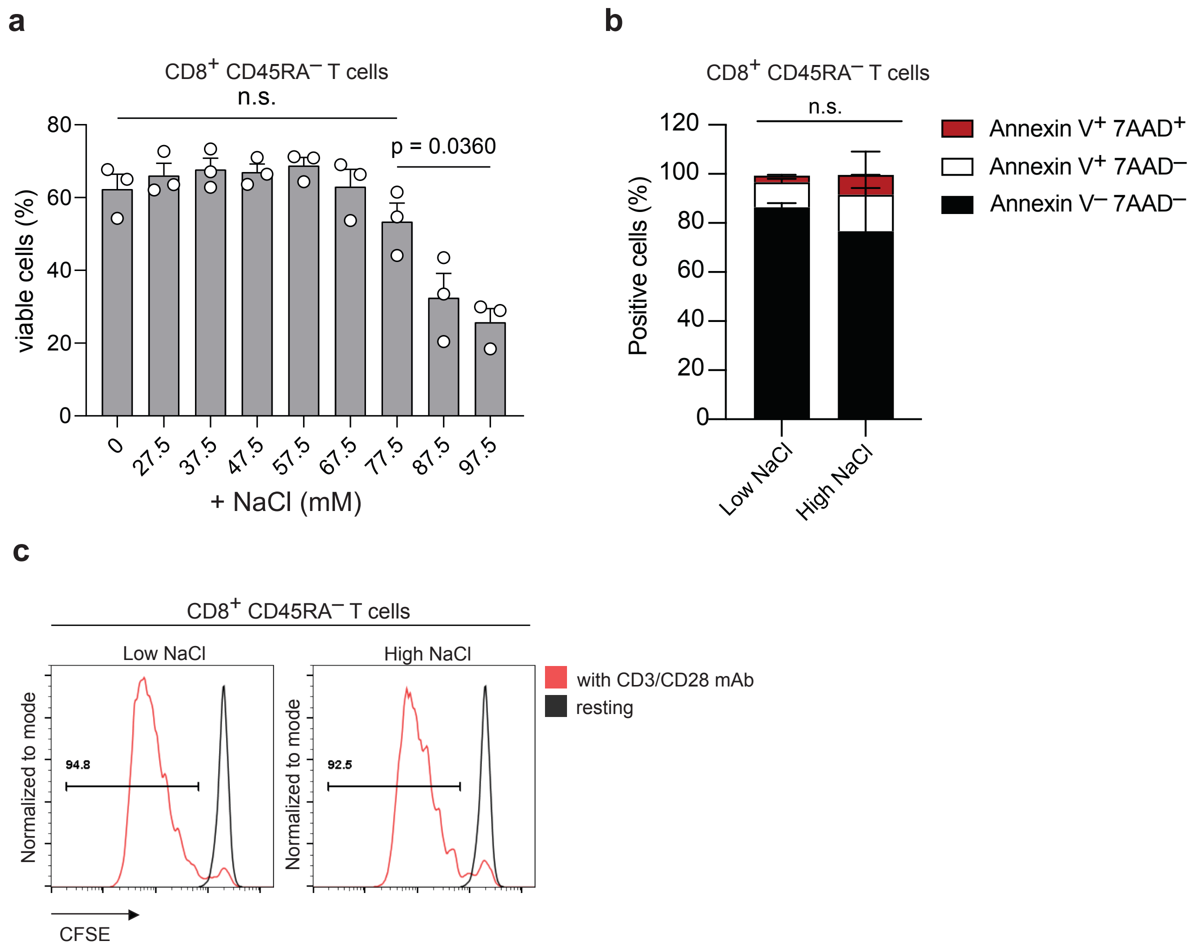

Supplementary Figure 8

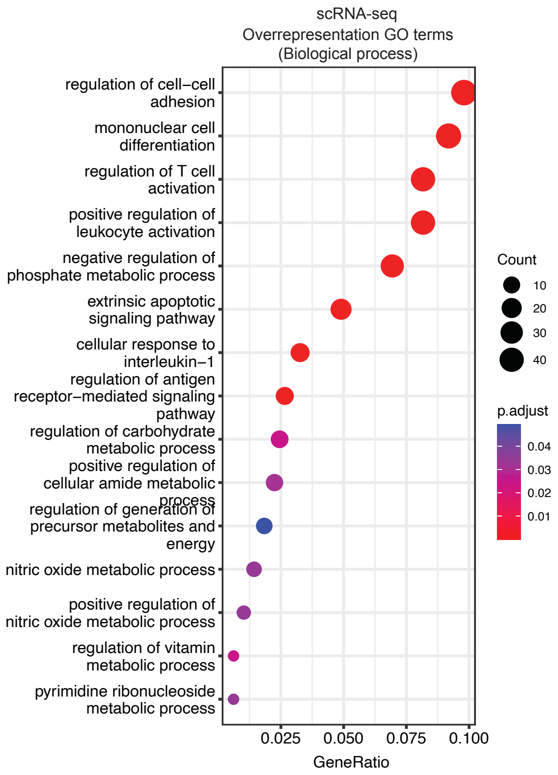

Supplementary Figure 9

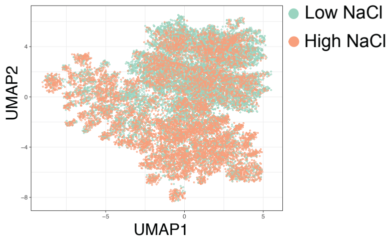

Supplementary Figure 10

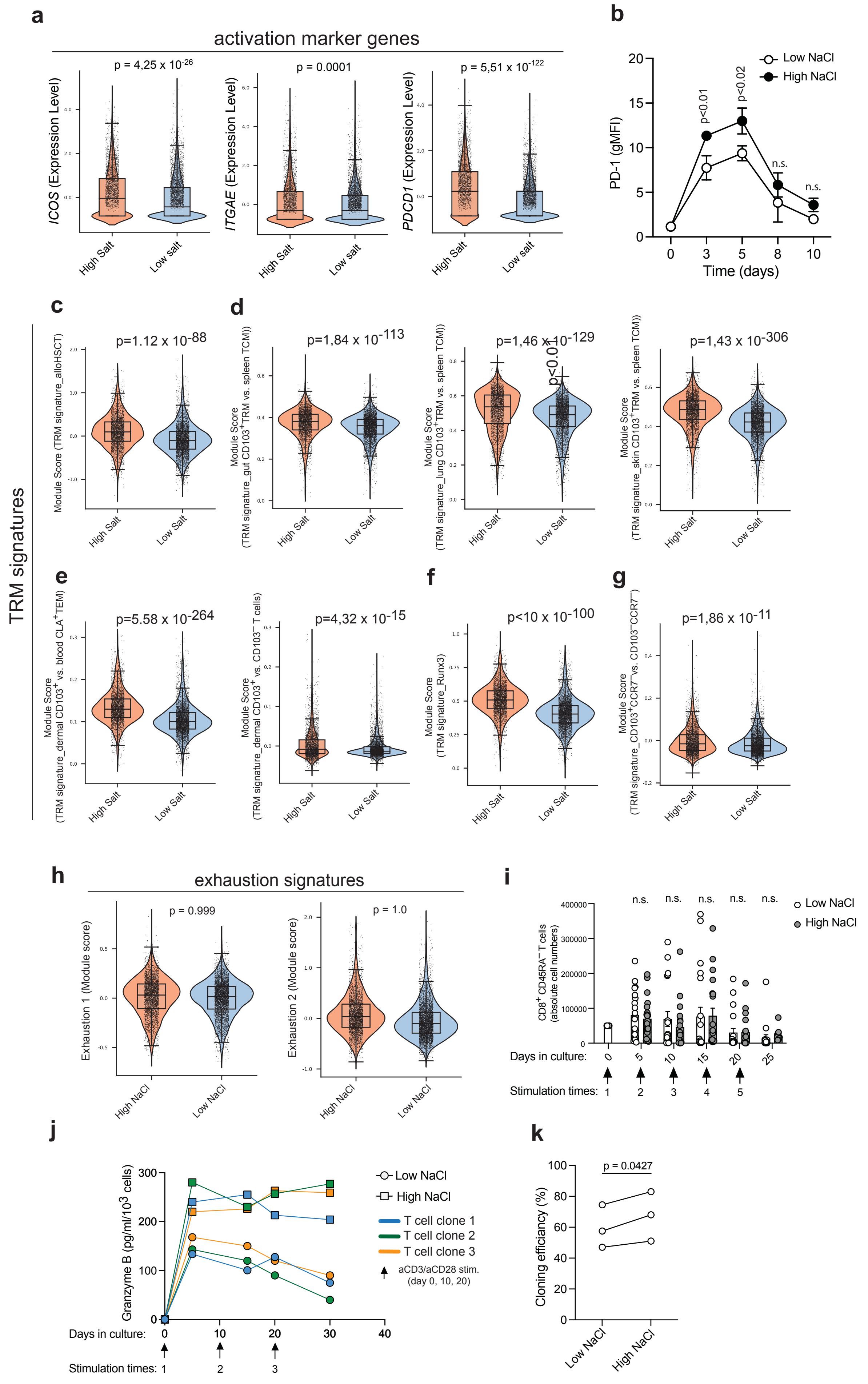

Supplementary Figure 11

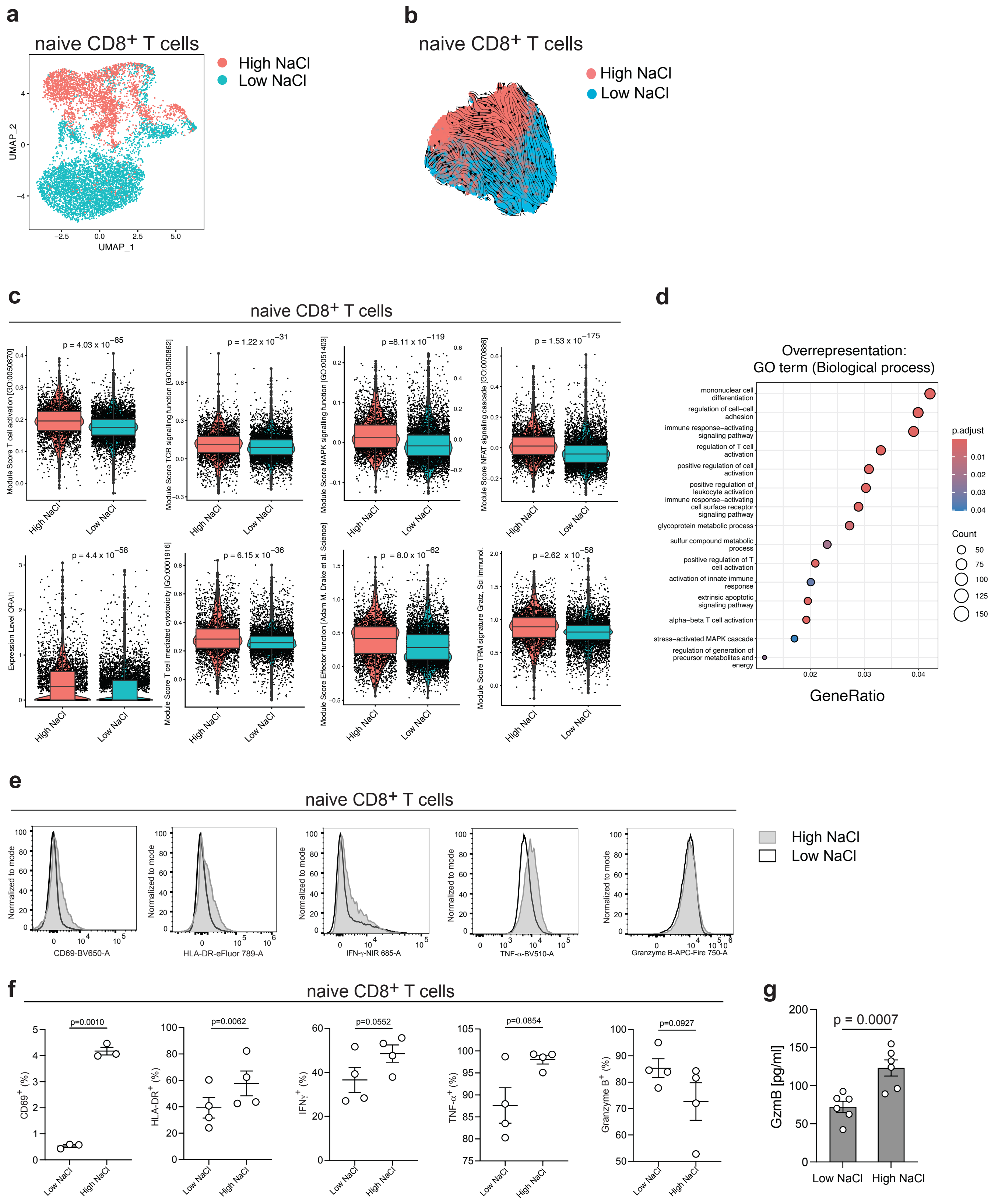

Supplementary Figure 12

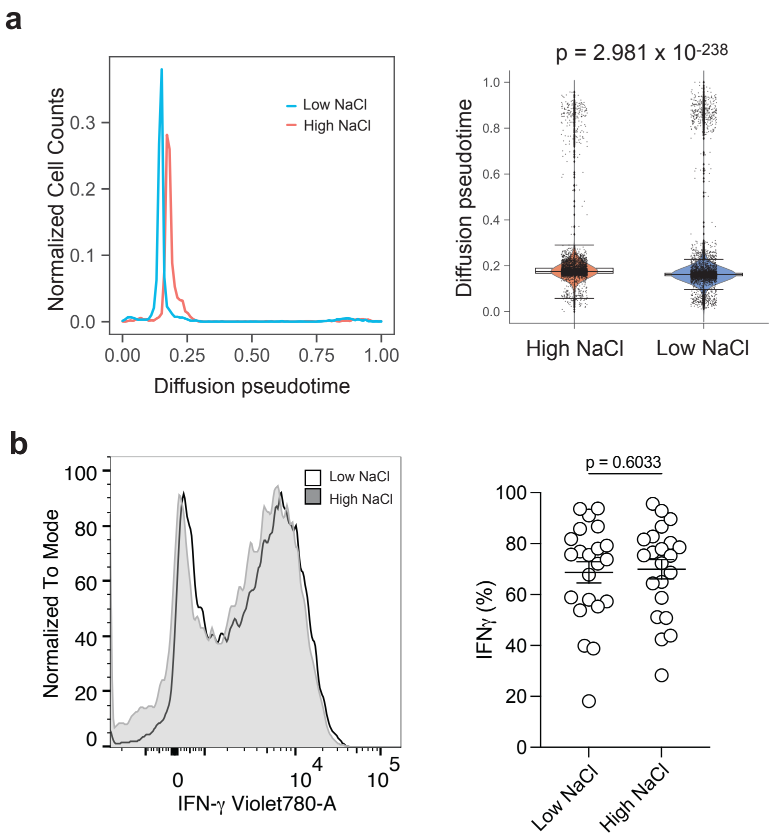

Supplementary Figure 13

a

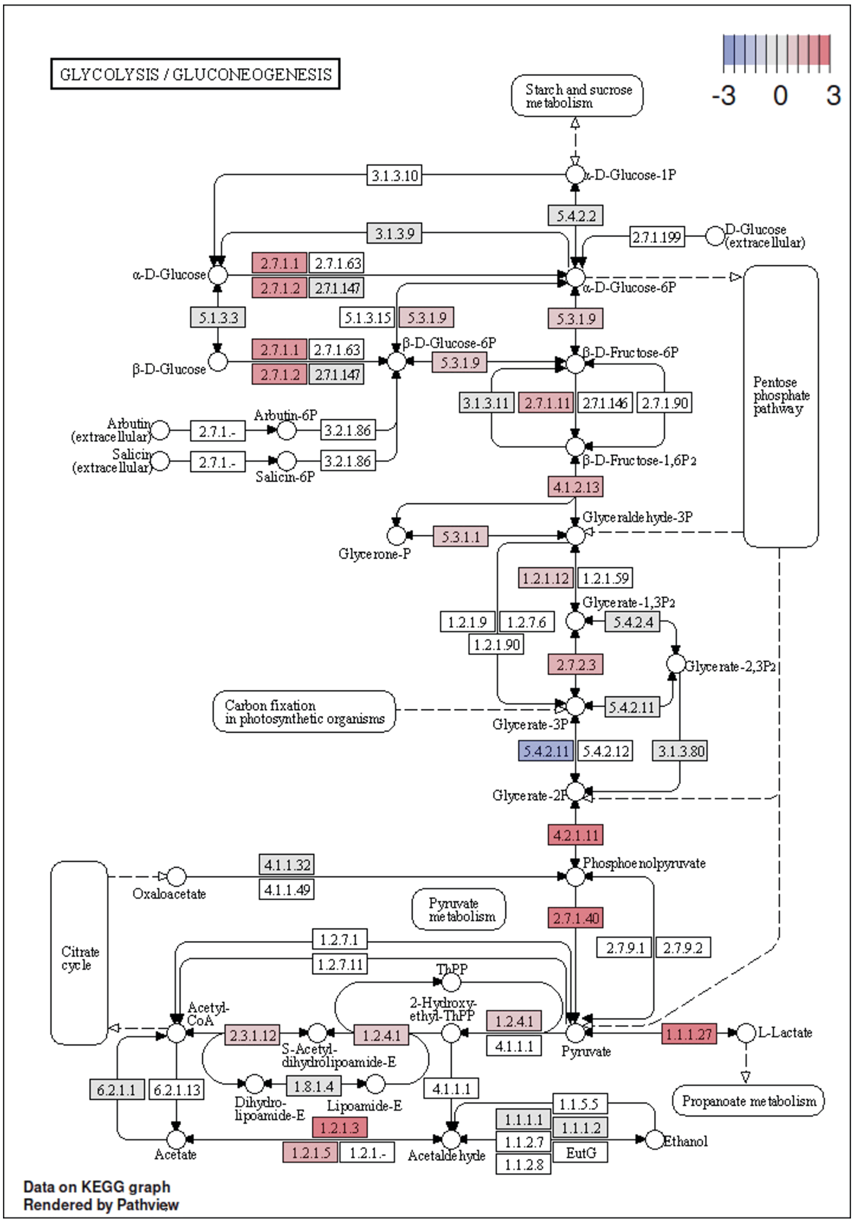

b

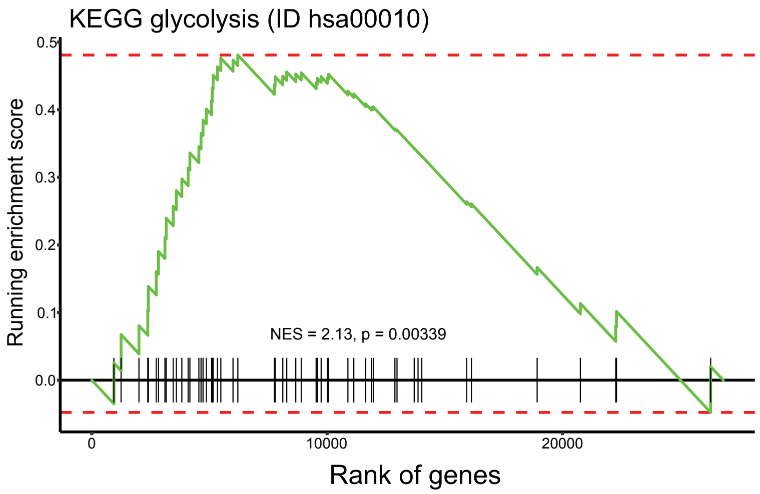

Supplementary Figure 14

a

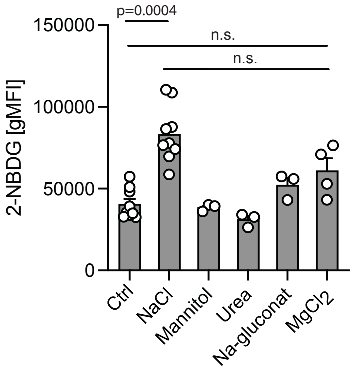

b

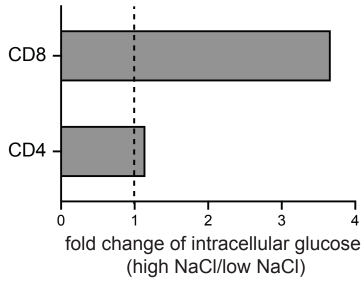

Supplementary Figure 15

CD8+ CD45RA– T cells

Low NaCl High NaCl

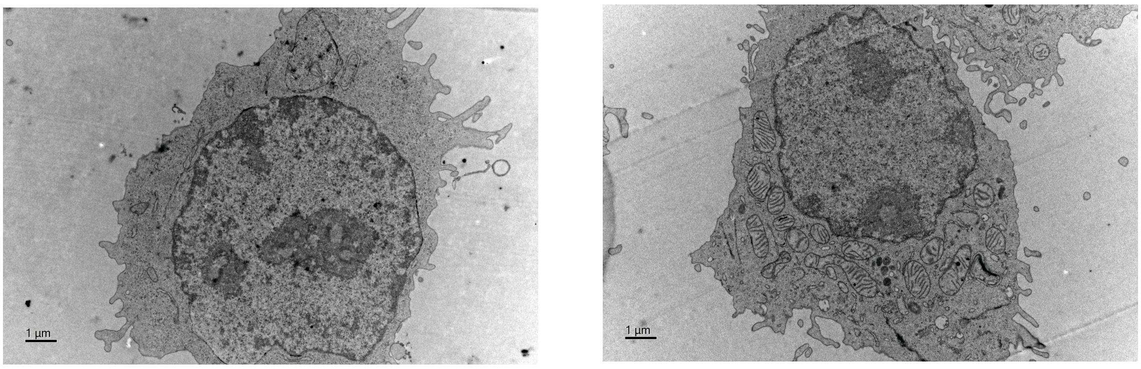

Supplementary Figure 16

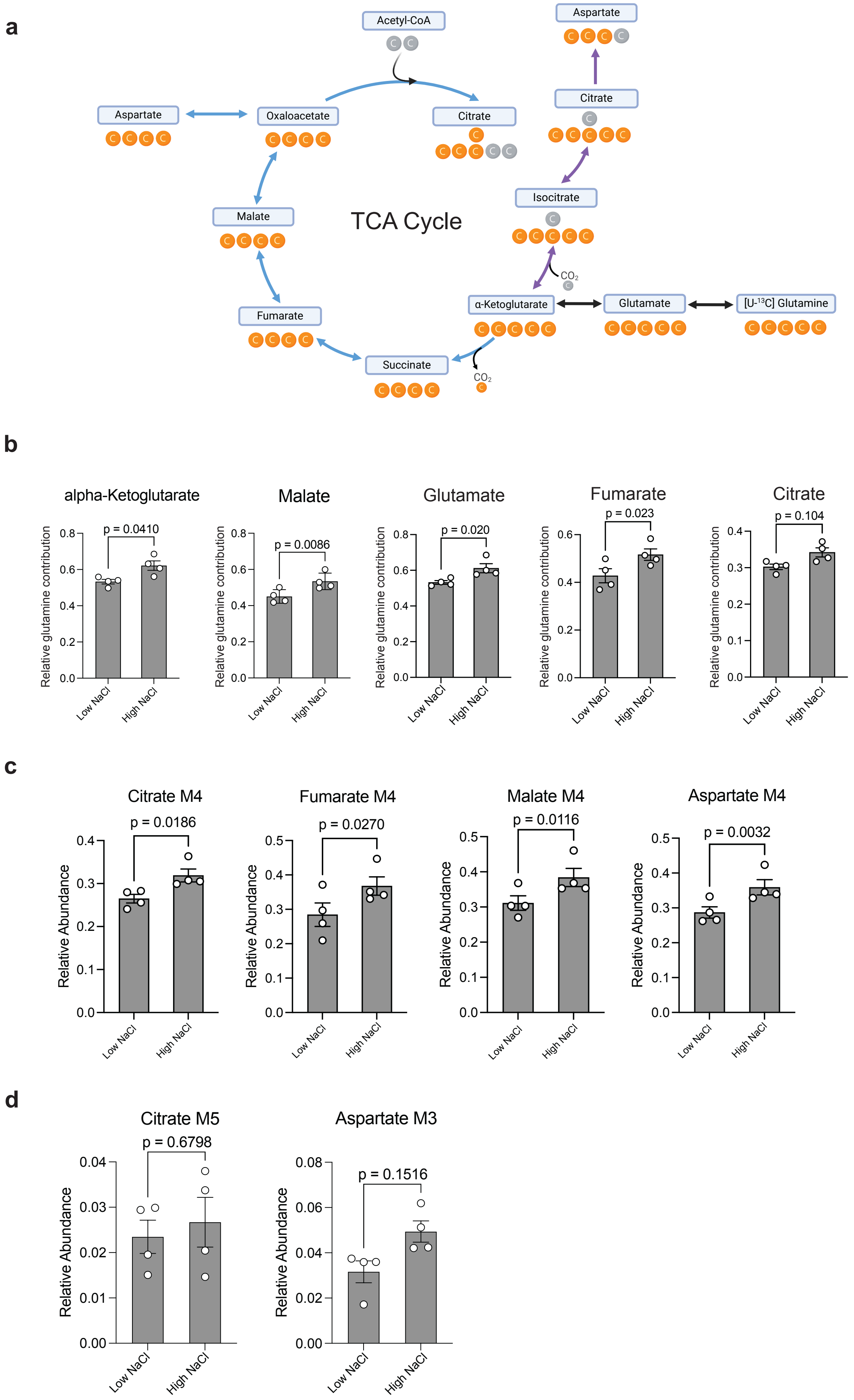

Supplementary Figure 17

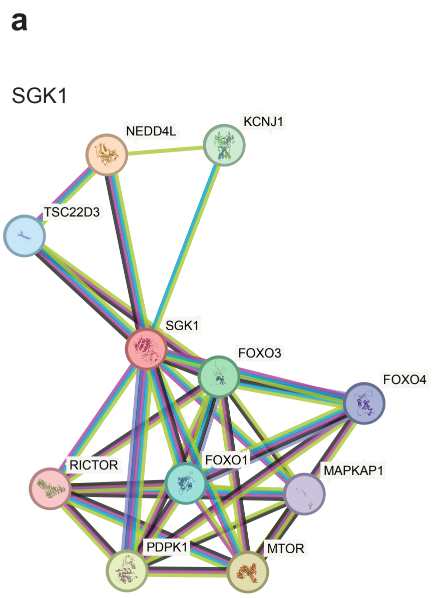

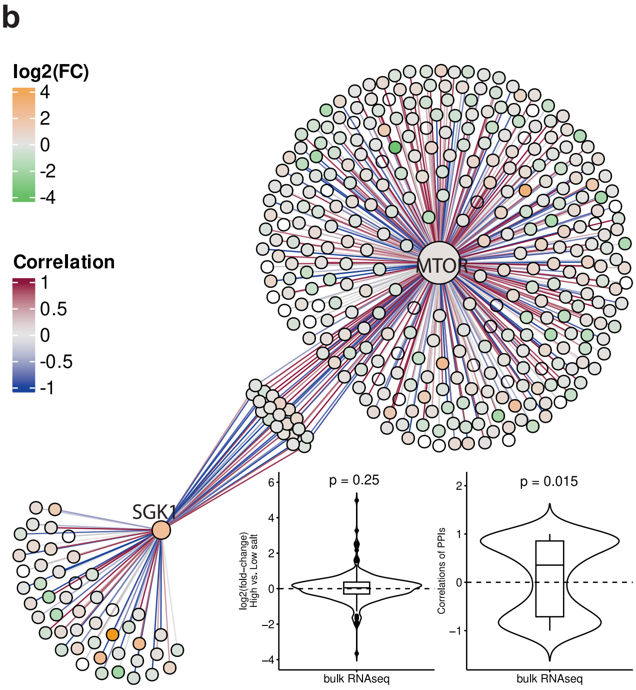

Supplementary Figure 18

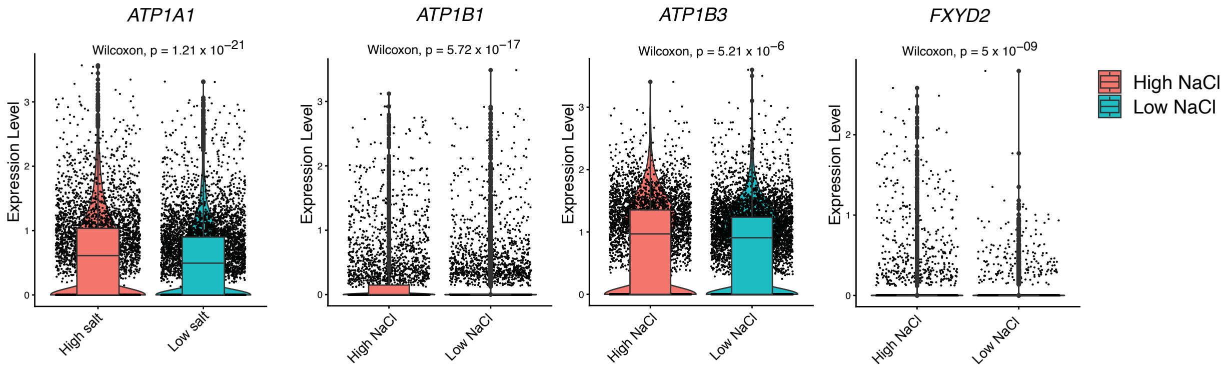

Supplementary Figure 19

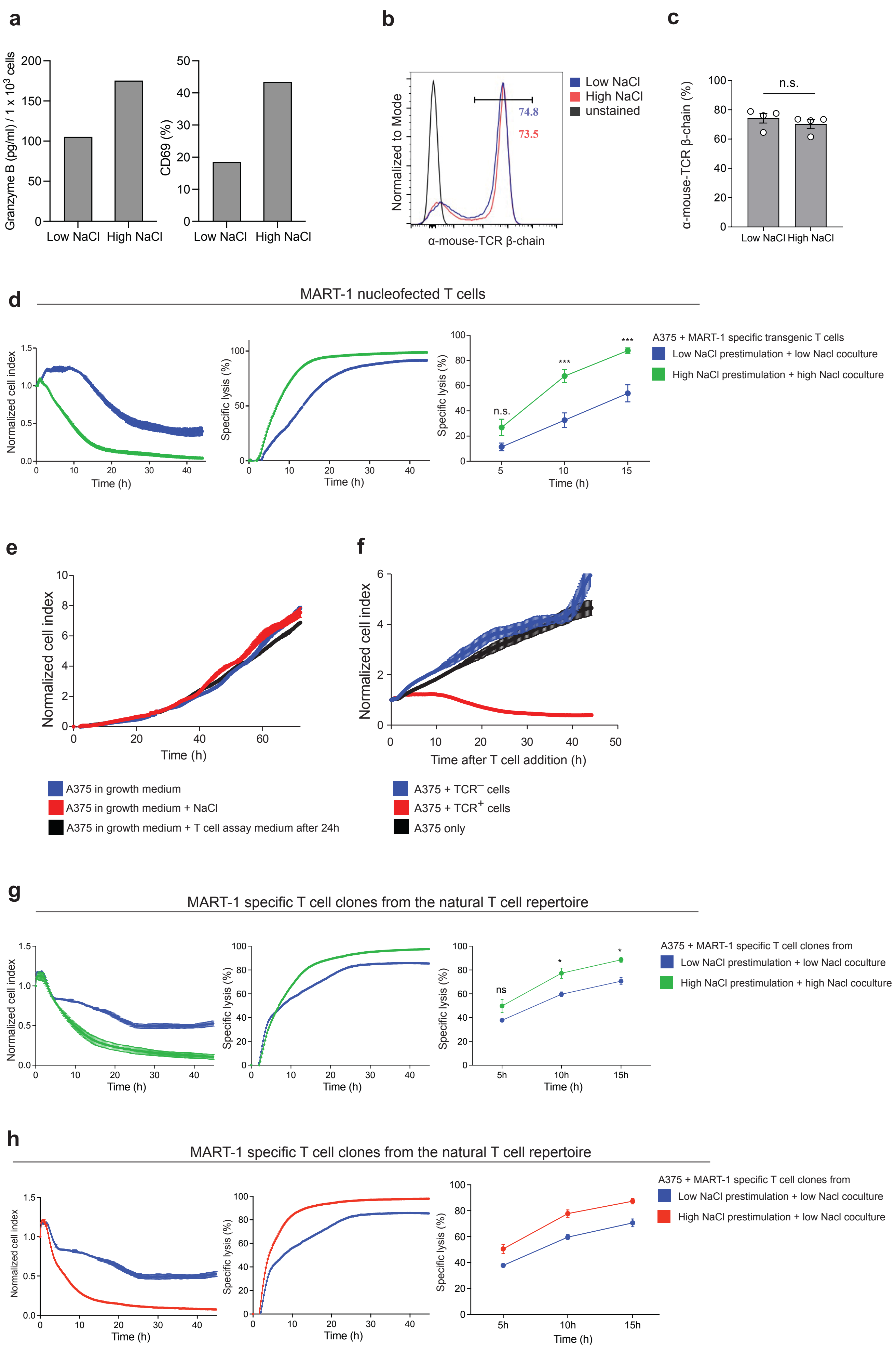

Supplementary Figure 20

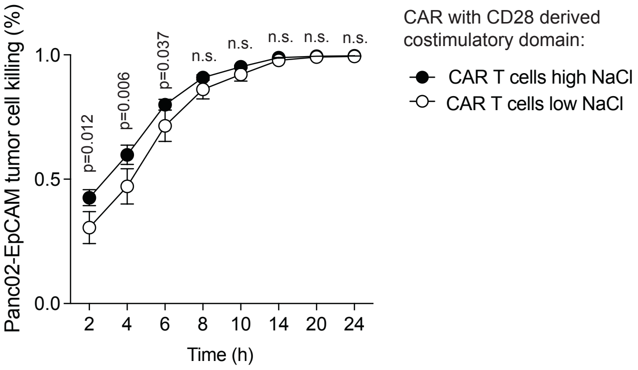

Supplementary Figure 21

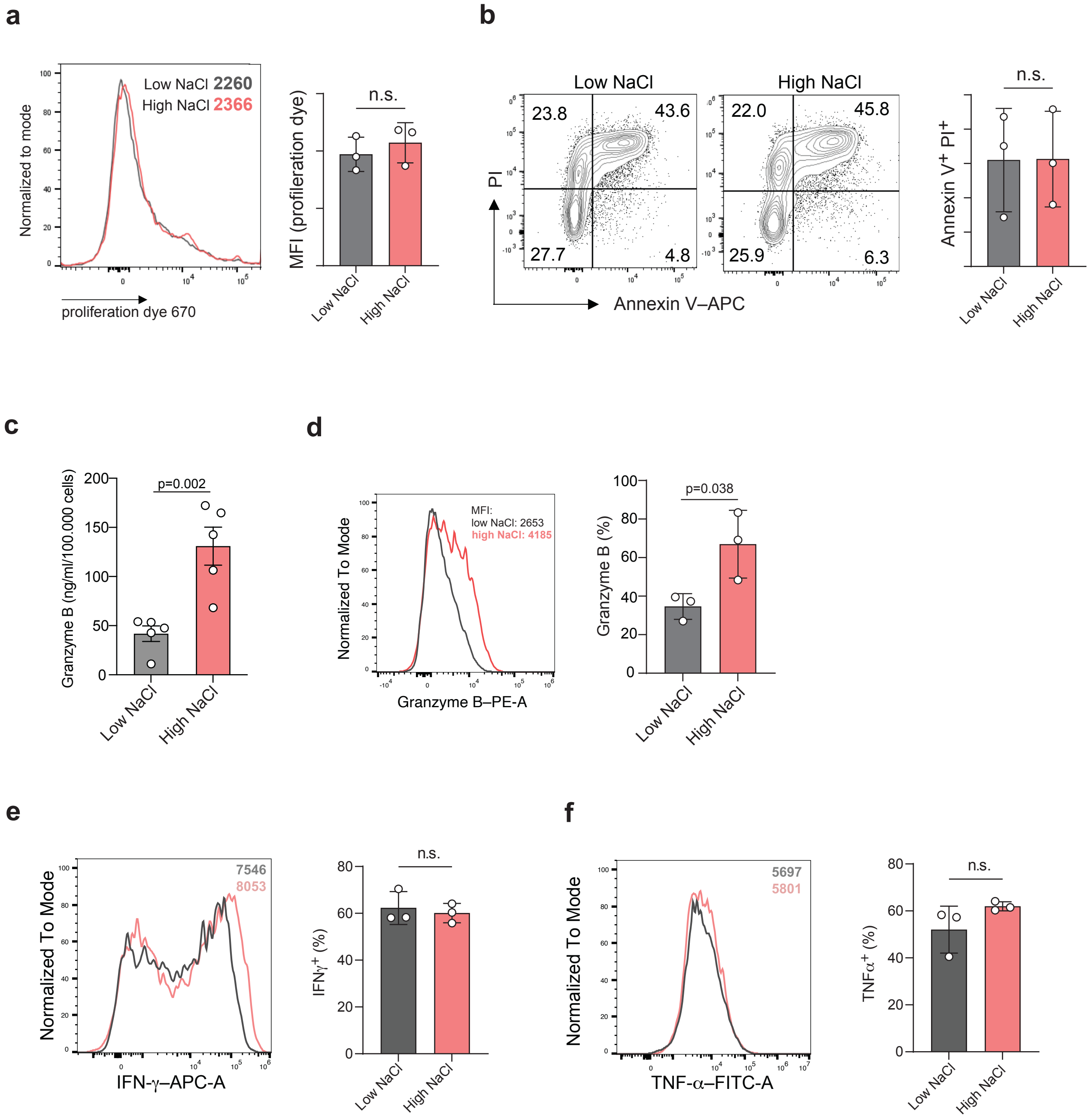

Supplementary Figure 22

a

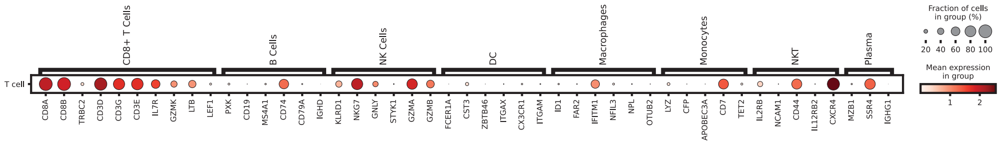

b

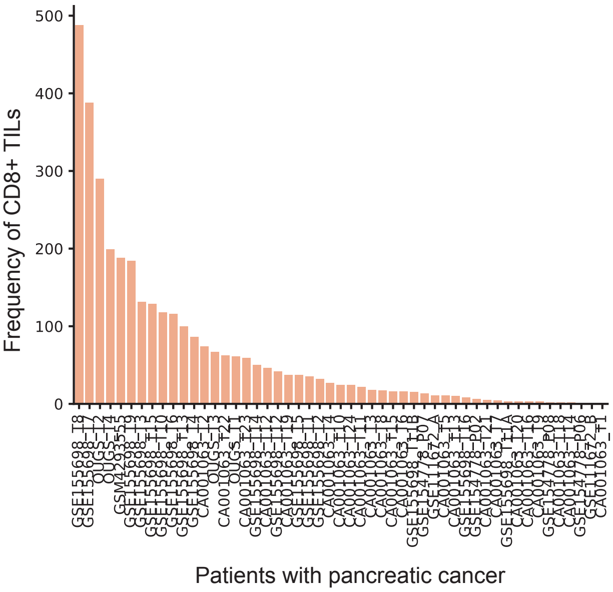

Supplementary Figure 23

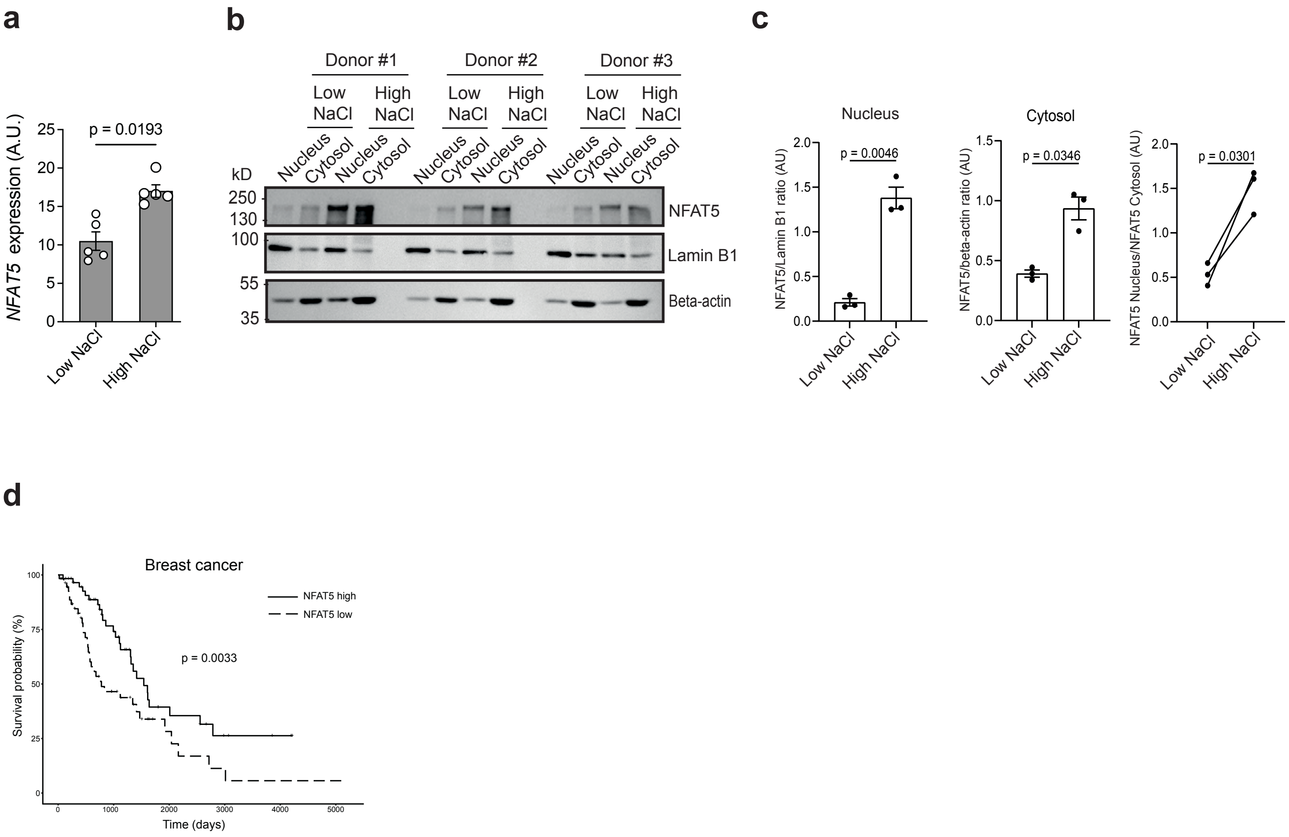

Supplementary Figure 24

uncropped blots

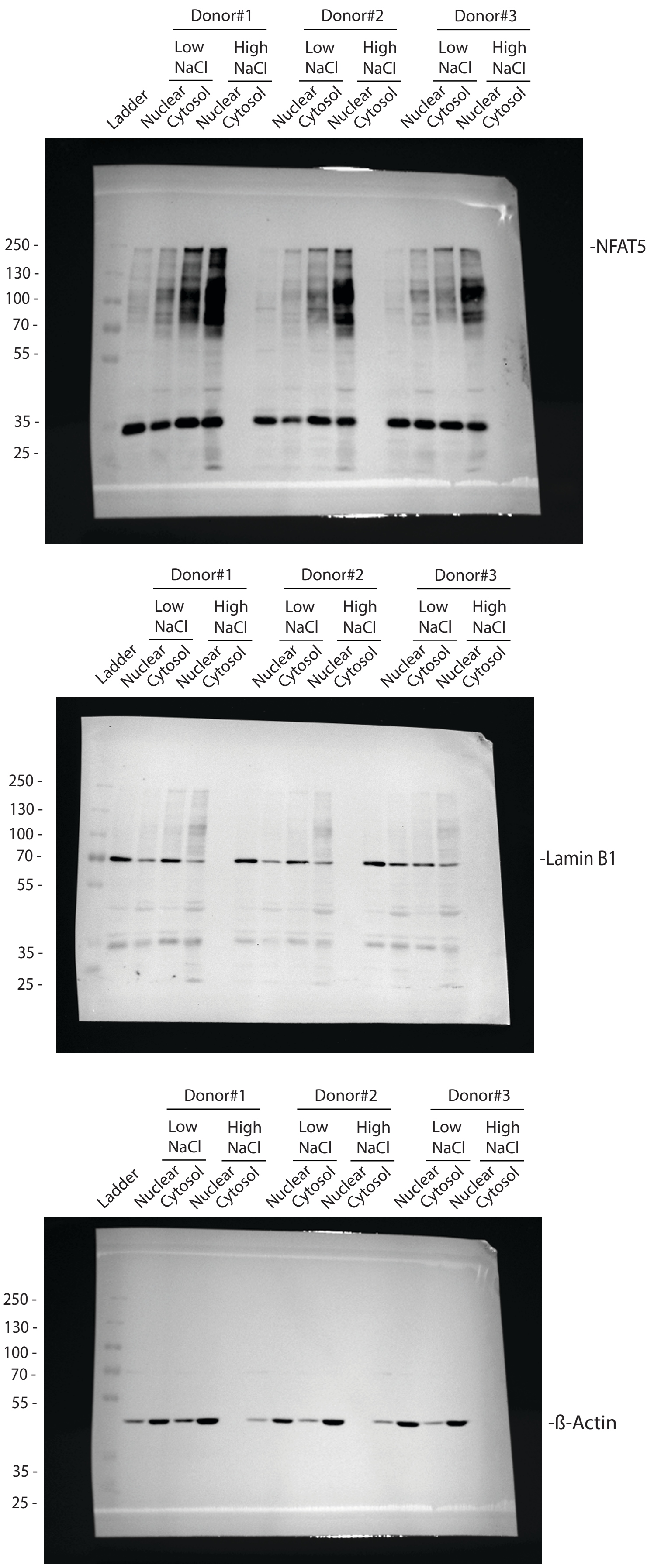

Supplementary Figure legends

Supplementary Figure 1. A wide range of solid tumors displays an enrichment of a transcriptomic NaCl signature. Barcode plots resulting from preranked gene set enrichment analysis (GSEA) using an adaptive multilevel split Monte Carlo scheme as implemented in the R-package FGSEA based on tumor tissue data from The Cancer Genome Atlas (TCGA). Enrichment score and sorted position of the 1956 significantly upregulated genes in the salt signature are shown. NES: normalized enrichment score, p: significance of the enrichment (one-tailed test for positive enrichment), n_1: patients providing tumor samples, n_2: patients providing healthy samples.

Supplementary Figure 2. Single-cell transcriptomic annotation of CD8$^+$ T cells from intratumoral breast cancer and healthy peritumoral tissues. a, Marker gene expression for all CD45$^+$ immune cells from the matched tumoral and peritumoral tissues from three patients with breast cancer (GSE114727). **b**, **c**, Marker gene expression for T cells (b) and CD8$^+$ T cells (c) from the matched tumoral and peritumoral tissues from three patients with breast cancer after filtering for these immune cell subsets using Celltypist.

Supplementary Figure 3. Intratumoral CD8$^+$ T cells from patients with breast cancer have an increased scRNA-seq derived NaCl signature. The module score for the scRNA-seq NaCl signature was tested in intra- and peritumoral CD8$^+$ T cells (filtered from CD45$^+$ immune cells by Celltypist, GSE114727) from 3 individual patients with breast cancer. The "scRNA-seq NaCl signature" was established from the significant DEGs after performing scRNA-seq of CD8$^+$ CD45RA$^-$ T cells stimulated for 3 days with CD3 and CD28 mAbs under high and low conditions. Wilcoxon rank sum test.

Supplementary Figure 4. Top differentially expressed genes from a comparison of memory CD8$^+$ T cells cultured under high and low NaCl conditions. a, The top 20 significantly up- (upper panel) and down-regulated (lower panel) genes from a comparison of all CD8$^+$ CD45RA$^-$ T cells from high and low NaCl conditions following scRNA-seq are shown. **b**, The top 20 significantly up- (upper panel) and down-regulated (lower panel) genes from a comparison of the high NaCl-Leiden clusters (2, 3, 10, 11) with all other clusters after scRNA-seq of human CD8$^+$CD45RA$^-$ T cells from high and low NaCl conditions on day 3 after stimulation with CD3 and CD28 mAbs are shown.

Supplementary Figure 5. Transcriptomic comparison of CD8$^+$ memory T cells from high and low NaCl conditions. a, Gating strategy for sorting CD8$^+$ CD45RA$^-$ T cells. **b,** CD8$^+$ CD45RA$^-$ T cells from 3 independent healthy donors were stimulated with CD3 and CD28 mAbs under high and low NaCl conditions for 5 days before mRNA-seq analysis. The number of significantly up- and down-regulated genes across the whole transcriptome is shown. **c,** Shown are heatmaps of CD8$^+$ CD45RA$^-$ T cells stimulated as in (a) displaying the top 50 up- (left side) and top 50 down-regulated (right side) DEG ordered according to significance (Benjamini-Hochberg adjusted p≤0.05 of DESeq2 based significance test). Log_2(fold-change) for high vs. low NaCl.

Supplementary Figure 6. Marker gene expression of Leiden clusters formed by the differential clustering of CD8$^+$ memory T cells from high and low NaCl conditions. a, scRNA-seq analysis and Leiden clustering of CD8$^+$ CD45RA$^-$ T cells stimulated with CD3 and CD28 mAbs for 3 days under high and low NaCl conditions. The top 10 marker genes per cluster were determined by the wilcoxauc() function from the R package presto (version 1.0.0).

The heatmap was created using the R package ComplexHeatmap (v.2.12.1). **b,** Marker genes for cluster 12 are highlighted.

Supplementary Figure 7. The viability of human CD8$^+$ CD45RA$^-$ T cells is maintained upon stimulation under high NaCl conditions. a, Human CD8$^+$ CD45RA$^-$ T cells were stimulated with CD3 and CD28 mAbs for 5 days in the presence of increasing (titrated) extracellular NaCl concentrations. Flow cytometric analysis of viability according to live/dead marker dye exclusion. n=3. Mean ± SEM. One-way ANOVA. **b**, Flow cytometric analysis of the indicated markers on human CD8$^+$ CD45RA$^-$ T cells stimulated as in (a). n=3. mean ± S.D. One-way ANOVA. **c**, Flow cytometric analysis of proliferation by CFSE dilution in cells stimulated as in (a, + 67,5 mM NaCl). Shown is one representative experiment (n=3 independent experiments).

Supplementary Figure 8. CD8$^+$ CD45RA$^-$ T cells from high compared to low NaCl culture conditions show transcriptomic enrichment for processes indicating invigorated immune responses. scRNA-seq analysis of human CD8$^+$ CD45RA$^-$ T cells stimulated for 3 days under high and low NaCl conditions was performed. Unbiased enrichment based on overrepresentation analysis using ClusterProfiler was computed ($p \leq 0.05$ as significance threshold after hypergeometric test). The top 15 processes within the GO terms for "Biological Process" are shown.

Supplementary Figure 9. High-dimensional spectral flow cytometry. Spectral flow cytometry with 33 protein surface markers of CD8$^+$ CD45RA$^-$ T cells on day 5 after activation with CD3 and CD28 mAb in the indicated conditions. Dimensionality reduction visualization

with UMAP highlighting the high and low NaCl conditions according to the colour code. n = 4 biological replicates.

Supplementary Figure 10. Human $CD8^+$ $CD45RA^-$ T cells from high NaCl conditions are characterized by increased activation states, enhanced T cell residency and absence of exhaustion. a, scRNA-seq and gene expression analysis of human $CD8^+$ $CD45RA^-$ T cells stimulated with CD3 and CD28 mAbs for 3 days under high and low NaCl conditions. Wilcoxon rank sum test. **b,** Flow cytometry after stimulation of $CD8^+CD45RA^-$T cells under high and low NaCl conditions with CD3 and CD28 mAbs for the indicated time. n=3, mean ± S.E.M., two-way ANOVA ($p<0.0001$) with multiple comparisons Student's t test (Benjamini) for high vs. low NaCl conditions. **c-g,** Module score analysis for T cells as in (a). Wilcoxon rank sum test. **c**, The T_{RM} signature was established from significantly DEGs following scRNAseq of human skin $CD8^+$ T cells of host versus donor origin in the skin more than 2 years after allo-HSCT [27]; **d,** from significantly DEGs following a bulk transcriptomic comparison of murine gut, lung and skin $CD103^+$ T_{RM} cells versus spleen T_{CM} cells [31]; **e,** from significantly DEGs following a bulk transcriptomic comparison of (left) human dermal $CD103^+$ versus blood CLA^+ T_{EM} cells or (right) dermal $CD103^+$ versus dermal $CD103^-$ T cells [97]; **f**, from a T_{RM} core signature from murine nonlympoid tissues and tumors [29]; **g**, from from significantly DEGs following bulk transcriptomic comparison of human $CD103^+CCR7^-$ versus $CD103^-CCR7^-$ T cells [30]. **h,** scRNA-seq and analysis of exhaustion module scores (left, Exhaustion1 [32]; right, Exhaustion 2 [98]) for human $CD8^+$ $CD45RA^-$ T cells stimulated as in (a). One-tailed (less) Wilcoxon rank sum test. **i**, Flow cytometry to determine absolute cell numbers (counting beads) for individual $CD8^+$ T cell clones after each restimulation with CD3 and CD28 mAbs. n=20, mean ± S.E.M., paired two-way ANOVA with uncorrected Fischer's LSD. **j**, ELISA of cell culture supernatants from $CD8^+$ T cell clones. n=3 (T cell clones). **k**, The cloning

efficiency was determined as the percentage of growing T cell clones on day 10 after single cell deposition of 200 CD8$^+$ CD45RA$^-$ T cells that had been prestimulated with CD3 and CD28 mAbs under either high or low NaCl conditions for 5 days and then expanded in T cell cloning conditions. Each circle indicates an independent experiment and blood donor. Two-tailed paired Student's t test.

Supplementary Figure 11. Phenotypic, functional and transcriptomic changes of naïve CD8$^+$ CD45RA$^+$ T cells upon TCR stimulation under high compared to low NaCl conditions. a, scRNA-seq and UMAP analysis of CD8$^+$ CD45RA$^+$ T cells following 3 days of stimulation with CD3 and CD28 mAbs in high and low NaCl conditions. **b,** Velocity analysis of cells stimulated and isolated as in (a). **c,** Analysis of modules scores of the indicated gene sets or genes. Wilcoxon rank sum test. **d,** Overrepresentation analysis using significantly upregulated DEGs from the transcriptomic comparison of CD8$^+$ CD45RA$^+$ T cells stimulated under high versus low NaCl conditions. Shown are the GO terms from Biological Processes. **e, f,** Flow cytometry of CD8$^+$CD45RA$^+$ stimulated as indicated in (a). **e,** representative experiments. **f,** Cumulative data with each circle representing a different blood donor. n=4, mean ± S.E.M., two-tailed paired Student's t test. **g,** ELISA with cell culture supernatants from CD8$^+$CD45RA$^+$ T cells that were stimulated as in (a, b) for 5 days. Paired student's t test.

Supplementary Figure 12. NaCl does not affect the expression of IFN-g in human CD8$^+$ memory T cells. **a,** Trajectory analysis of CD8$^+$ T cells stimulated under high and low NaCl conditions followed by scRNA-seq. Left, Diffusion Pseudotime (DPT) visualization and quantification by distribution of normalized cell counts along the diffusion pseudotime for both treatment conditions. Right, quantification. Two-sided Wilcoxon rank sum test and Benjamini Hochberg adjusted p-value with 0.05 significance threshold. **b**, Intracellular staining and flow

cytometry for IFN-γ on day 5 after stimulation with CD3 and CD28 mAbs and PMA/ionomycin restimulation on day 5 in the presence of brefeldin A. Left, representative experiment. Right, cumulative data. n = 22, mean ± S.E.M., two-tailed paired Student's t test.

Supplementary Figure 13. High NaCl conditions promote glycolysis in human $CD8^+$ $CD45RA^-$ T cells on the transcriptomic level. a, KEGG pathway analysis. Expression levels of the individual glycolysis-associated genes following bulk mRNA-seq of human $CD8^+$ $CD45RA^-$ T cells from high and low NaCl conditions are shown. Color range indicates $\log_2$(fold-change) for comparison high vs. low NaCl for enzyme-associated genes according to the R-package pathview. **b,** Preranked gene set enrichment analysis (GSEA) by fold change for high vs low salt of glycolysis associated genes (KEGG pathway: glycolysis). Significance by permutation test (p = 0.00339) of running enrichment score for positive enrichment based on an adaptive multilevel split Monte Carlo scheme as implemented in the R-package FGSEA according to FGSEA R package.

Supplementary Figure 14. NaCl but not other osmolytes increases glucose uptake by human $CD8^+$ $CD45RA^-$ T cells. a, Flow cytometric assessment of glucose uptake into human $CD8^+$ $CD45RA^-$ T cells that were stimulated for 5 days with CD3 and CD28 mAbs under high and low NaCl conditions. One-way ANOVA. **b,** Nontargeted metabolic profiling of $CD8^+$ and $CD4^+$ $CD45RA^-$ T cells stimulated as in (a) by liquid chromatography-tandem mass spectrometry (LC–MS). n=4, mean ± S.E.M.

Supplementary Figure 15. Mitochondriae from $CD8^+$ T cells that are cultured under high compared to low NaCl conditions are bigger and display more cristae. Transmission

electron microscopy of $CD8^+CD45RA^-$ T cells that were stimulated with CD3 and CD28 mAbs for 5 days under high and low NaCl conditions.

Supplementary Figure 16. High NaCl conditions increase glutaminolysis in $CD8^+CD45RA^-$ T cells. a, Atom transitions for TCA cycle metabolites with [U-^{13}C] glutamine tracing. ^{13}C atoms are depicted in orange and ^{12}C atoms in grey. Blue arrows, oxidative TCA cycle flux. Purple arrows, reductive TCA cycle flux. **b**, Relative contribution of glutamine to the generation of the indicated metabolites after 24 h culture with the [U-^{13}C] glutamine tracer from day 4 after stimulation of $CD8^+CD45RA^-$ T cells with CD3 and CD28 mAbs under low and high NaCl conditions. Values are corrected for natural isotope abundance. **c**, Oxidative decarboxylation represented by the relative abundance of the indicated M4 isotopologues in $CD8^+CD45RA^-$ T cells stimulated as in (b). **d**, Reductive carboxylation represented by the relative abundance of M5 citrate and M3 aspartate in $CD8^+CD45RA^-$ T cells stimulated as in (b,c). b-d, n=4, mean ± S.E.M., two-tailed paired Student's t test.

Supplementary Figure 17. Protein-protein interactions networks demonstrate a correlation of NaCl-sensing with metabolic signaling and TCR activation. a, STRING analysis for SGK1 using default parameters. **b**, Protein-protein interaction network after combining curated BioGRID protein-protein interaction information for SGK1 and MTOR. Node color reflects $\log_2$(fold-change) of expression in the bulk RNA sequencing data (high vs. low NaCl), while edge color shows Pearson correlation of expression. Two-tailed Wilcoxon test on the $\log_2$(fold-changes) for expression or correlation of expression for comparison high vs. low NaCl. Boxplot statistics: $\log_2$(fold-change) of expression changes (high vs. low NaCl): min=-3.656, max=4.976, Q1=-0.303, median=0.047, Q3=0.369, lower whisker=-0.956, upper whisker=0.952; $\log_2$(fold-change) of correlation changes for comparison high vs. low NaCl:

min=-1.000, max=1.000, Q1=-0.713, median=0.356, Q3=0.856, lower whisker=-0.984, upper whisker=0.992.

Supplementary Figure 18. NaCl induces the upregulation of NKA genes in CD8$^+$ CD45RA$^-$ T cells. Single-cell mRNA-seq of CD8$^+$CD45RA$^-$ T cells stimulated with CD3 and CD28 mAb for 72h under high and low NaCl conditions. Wilcoxon rank sum test.

Supplementary Figure 19. NaCl-induced enhanced killing of target cells by CD8$^+$ T cells is antigen specific and not confounded by direct effects of NaCl on target cells. a, ELISA of cell culture supernatants after 5 days of stimulation of MART-1 nucleofected CD8$^+$CD45RA$^-$ T cells with CD3 and CD28 mAbs under high or low NaCl conditions (left). Flow cytometry of the same cells (a) on day 5 (right). **b, c,** Flow cytometry of sorted CD8$^+$ T cells, which were expanded after nucleofection with a MART-1-specific TCR for 5 days with CD3 and CD28 mAbs under high versus low NaCl conditions. (b), Representative experiment. (c), Cumulative quantification (mean ± S.E.M., n=3, two-tailed paired student's t test). **d, g**, **h**, Coculture of MART-1-specific nucleofected CD8$^+$ T cells **(d)** and CD8$^+$ T cell clones from the natural T cell repertoire **(g)** with A375 melanoma cells (1:1 ratio) pulsed with MART-1 peptide in the presence or absence of additional NaCl or only absence of NaCl (h) after prestimulation for 5 days with CD3 and CD28 mAbs under high versus low NaCl conditions. One representative experiment is shown. **d**, n=3 experiments; **g**, n=4 T cell clones, **h**, n=2 T cell clones, mean ± S.E.M., two-way ANOVA, **, p<0.001. **e,** A375 melanoma cell line cells were seeded on E-plates, and growth was monitored for 72 h under the indicated conditions. n=4, one representative experiment is shown. **f**, Coculture of MART-1-specific (TCR$^+$) or MART-1-nonspecific CD8$^+$ T cells (TCR$^-$, α-mouse TCRβ chain negative after nucleofection) with

A375 melanoma cells (1:1 ratio) pulsed with MART-1 peptide under high versus low NaCl conditions. One representative experiment is shown, n=4.

Supplementary Figure 20. High NaCl conditions license CAR T cells for superior killing of tumor cells. Murine EpCAM-CAR T cells were generated and cultured for 48 h under high and low NaCl conditions and then cocultured with Panc02-EpCAM target cells at a 2.5:1 ratio. Antigen-specific lysis of Panc02-EpCAM cells by $CD8^+$ CAR T cells was determined at different time points. n=3 independent experiments. n = 3 independent experiments, mean ± S.D., two-way ANOVA.

Supplementary Figure 21. High NaCl conditions do not compromise the viability of mouse $CD8^+$ T cells. a, b, Flow cytometric analysis of $CD8^+$ T cells after stimulation with CD3 and CD28 mAbs for 3 days under high and low NaCl conditions (n = 3). Left, representative experiment with mean fluorescence intensities is shown; right, cumulative quantification. n =3, mean ± S.D., two-tailed paired Student's t test. **c**, ELISA with cell culture supernatants from $CD8^+$ T cells stimulated for 3 days with CD3 and CD28 mAbs under high and low NaCl conditions. **d, e, f,** Intracellular staining and flow cytometry for granzyme B (d), IFN-γ (c) or TNF (d) on day 3 after *in vitro* stimulation with CD3 and CD28 mAbs for 3 days. Left, representative experiment; right, cumulative quantification. All data points represent biological replicates. n=3, mean ± S.D., two-tailed paired Student's t test. n.s., not significant.

Supplementary Figure 22. Identification of intratumoral $CD8^+$ T cells in whole tumors from patients with pancreatic cancer with scRNA-seq. $CD8^+$ T cells from 51 patients with pancreatic cancer identified in scRNA-seq data sets (integration of all cells: 10.5281/zenodo.6024273 [54]). **a,** Shown is the mean expression of different immune cell lineage

marker genes after filtering for $CD8^+$ T cells from the whole tumor single-cell transcriptomes. **b,** Shown is the frequency of $CD8^+$ T cells per patient.

Supplementary Figure 23. High NaCl conditions induce increased *NFAT5* gene expression and increased intranuclear NFAT5 translocation in $CD8^+CD45RA^-$ T cells, which correlates with improved breast cancer patient survival. a, qRT-PCR analysis of $CD8^+CD45RA^-$ T cells stimulated with CD3 and CD28 mAb for 5 days. n = 5, mean ± S.E.M., two-tailed paired Student's t-test. **b,** Western blot of nuclear and cytosolic cell fractions of T cells stimulated as in a. **c,** cumulative quantification of results shown in b. n=3 (Donor 1-3), mean ± S.E.M., two-tailed paired Student's t-test. **d**, Kaplan-Meier tumor-free survival probability of patients from the TCGA database diagnosed with breast cancer who differ by *NFAT5* expression levels in the tumor tissue. Patients were subgrouped by computing an optimal cutoff for *NFAT5* expression (TPM, Transcripts Per Kilobase Million normalized) towards overall survival outcome. Number of patient samples: n = 59 for *NFAT5* = high, n = 54 for *NFAT5* = low, significance of survival differences was determined with the Peto-Peto algorithm using the surv_pvalue function (method = "S1") as implemented in the R-package survminer.

Supplementary Figure 24. Uncropped western blots for Supplementary Figure 23b.

Supplementary tables

Supplementary table 1. Identification of differentially expressed genes in human $CD8^+$ memory T cells under high and low NaCl conditions using bulk mRNA-seq.

Supplementary data table 2. Differential gene expression analysis of human CD8$^+$ memory T cells stimulated under high and low NaCl conditions using scRNA-seq.

Supplementary table 3. Antibody panel for high-dimensional spectral flow cytometry of human CD8$^+$ T cells under high and low NaCl conditions.
