# Supplementary material for: Sodium chloride in the tumor microenvironment enhances T cell metabolic fitness and cytotoxicity
Source: Nat Immunol. 2024 Aug 28;25(10):1830–44. doi: 10.1038/s41590-024-01918-6 (PMC11436365; doi:10.1038/s41590-024-01918-6)
Supplement: Supplementary file 2 — Reporting Summary [file 41590_2024_1918_MOESM2_ESM.pdf]

nature portfolio

Corresponding author(s): Christina Zielinski

Last updated by author(s): 2020/06/19

Reporting Summary

Nature Portfolio wishes to improve the reproducibility of the work that we publish. This form provides structure for consistency and transparency in reporting. For further information on Nature Portfolio policies, see our Editorial Policies and the Editorial Policy Checklist.

Statistics

For all statistical analyses, confirm that the following items are present in the figure legend, table legend, main text, or Methods section.

n/a	Confirmed	
☐	☒	The exact sample size (*n*) for each experimental group/condition, given as a discrete number and unit of measurement
☐	☒	A statement on whether measurements were taken from distinct samples or whether the same sample was measured repeatedly
☐	☒	The statistical test(s) used AND whether they are one- or two-sided<br>*Only common tests should be described solely by name; describe more complex techniques in the Methods section.*
☒	☐	A description of all covariates tested
☒	☐	A description of any assumptions or corrections, such as tests of normality and adjustment for multiple comparisons
☐	☒	A full description of the statistical parameters including central tendency (e.g. means) or other basic estimates (e.g. regression coefficient) AND variation (e.g. standard deviation) or associated estimates of uncertainty (e.g. confidence intervals)
☐	☒	For null hypothesis testing, the test statistic (e.g. *F*, *t*, *r*) with confidence intervals, effect sizes, degrees of freedom and *P* value noted<br>*Give P values as exact values whenever suitable.*
☒	☐	For Bayesian analysis, information on the choice of priors and Markov chain Monte Carlo settings
☒	☐	For hierarchical and complex designs, identification of the appropriate level for tests and full reporting of outcomes
☒	☐	Estimates of effect sizes (e.g. Cohen's *d*, Pearson's *r*), indicating how they were calculated

Our web collection on statistics for biologists contains articles on many of the points above.

Software and code

Policy information about availability of computer code

Data collection	BD FACSAria Fusion (BD Biosciences), Cytek Aurora (CYTEK), CytoFLEX (Beckman Coulter), MACSQuant Analyzer (Miltenyi Biotec), BD LSRFortessa (BD Biosciences), BD FACSArialll (BD Biosciences), Spark Multimode plate reader (TECAN), CLARIOstar® (BMG Labtech), iBright analysis system (Thermo Fisher Scientific), JEM transmission electron microscope 1400 (JEOL), ICP-OES ARCOS (Ametek-Spectra), Seahorse XFp Analyzer (Agilent Technologies), xCELLigence SP Real-Time Cell Analyzer (ACEA Biosciences), 5977 MSD GC-MS system (Agilent Technologies), CFX Real-Time PCR instruments (Bio-Rad)
Data analysis	FlowJo (10.9.0) for FACS analysis. GraphPad Prism (version 7 to 10.1.2) for data analysis and plotting. All software packages in R or python have been referenced in the manuscript.

For manuscripts utilizing custom algorithms or software that are central to the research but not yet described in published literature, software must be made available to editors and reviewers. We strongly encourage code deposition in a community repository (e.g. GitHub). See the Nature Portfolio guidelines for submitting code & software for further information.

Data

Policy information about availability of data

All manuscripts must include a data availability statement. This statement should provide the following information, where applicable:

- Accession codes, unique identifiers, or web links for publicly available datasets
- A description of any restrictions on data availability
- For clinical datasets or third party data, please ensure that the statement adheres to our policy

All data points for the experiment are shown in the paper. All data points represent individual biological samples as indicated in the legends. snRNAseq data for naive and memory CD8+ T cells is available under GSE232149. In analyses, where publicly deposited raw data was used, the references specify data availability (GSE114727, GSE155698, GSE111672, GSE154778, GSM4293555, PRJCA001063 56). TCGA data base was used as indicated in the manuscript.

Research involving human participants, their data, or biological material

Policy information about studies with human participants or human data. See also policy information about sex, gender (identity/presentation), and sexual orientation and race, ethnicity and racism.

Reporting on sex and gender	Sex or gender is not considered in the experiment design, sex and/or gender was determined based on self-reporting.
Reporting on race, ethnicity, or other socially relevant groupings	Socially constructed or socially relevant categorization variables are not used in this manuscript.
Population characteristics	Participants have mixed gender, and mixed blood type, at the age from 19 to 68 years-old.
Recruitment	Healthy donors are recruited by the Blood bank of the University Hostpital Jena for blood donation. The University Hospital Jena Blood bank prepared buffy coats and performed serological tests to exclude Hepatitis B or C and HIV. Fresh blood from healthy volunteers tested negative for Hepatitis B, C and HIV were obtained from students of the Technical University of Munich.
Ethics oversight	The study was approved by the Ethical Committees (Institutional Review Board) of the Friedrich Schiller University Jena, by the Ethics Committee (Institutional Review Board) of the Charite Universitatsmedizin Berlin, by the Ethics Committee (Institutional review board) of the Technical Universiy Munich. All blood donors have provided their informed consent.

Note that full information on the approval of the study protocol must also be provided in the manuscript.

Field-specific reporting

Please select the one below that is the best fit for your research. If you are not sure, read the appropriate sections before making your selection.

☒ Life sciences ☐ Behavioural & social sciences ☐ Ecological, evolutionary & environmental sciences

For a reference copy of the document with all sections, see nature.com/documents/nr-reporting-summary-flat.pdf

Life sciences study design

All studies must disclose on these points even when the disclosure is negative.

Sample size	The sample sizes are indicated in the respective figures with circles representing each individual donors and experiments. Sample sizes were based on our experience and common practice in the field of human immunology (i.e. Nat Immunol. 2023 Jan; 295-308)
Data exclusions	No experimental data excluded from the analysis.<br>From the 56 pancreatic cancer patients (10.5281/zenodo.6024273) from which scRNAseq data was available, 5 have been excluded for further analysis because ob absence of CD8+ T cells.
Replication	Findings in experiments have been replicated from at least three independent experiments and confirmed technical reproducibility.
Randomization	Blood from healthy donors was used independent of gender or age. Allocation was performed randomly.
Blinding	Blinding was not relevant for this study as blood was derived from anonymous healthy donors from the blood bank. Experimental groups (high vs. low salt treatment) were not blinded.

Reporting for specific materials, systems and methods

We require information from authors about some types of materials, experimental systems and methods used in many studies. Here, indicate whether each material, system or method listed is relevant to your study. If you are not sure if a list item applies to your research, read the appropriate section before selecting a response.

Materials & experimental systems

n/a	Involved in the study
☐	☒ Antibodies
☐	☒ Eukaryotic cell lines
☐	☐ Palaeontology and archaeology
☐	☒ Animals and other organisms
☒	☐ Clinical data
☒	☐ Dual use research of concern
☒	☐ Plants

Methods

n/a	Involved in the study
☒	☐ ChIP-seq
☐	☒ Flow cytometry
☒	☐ MRI-based neuroimaging

Antibodies

Antibodies used

From BioLegend
1. PE/Dazzle594 mouse anti-human CCR4 (CD194) (BioLegend, 359420, L291H4, 1:200 dilution)
2. Alexa Fluor 647 mouse anti-human CD107a (LAMP-1) (BioLegend, 328612, H4A3, 1:500 dilution)
3. PerCP/Cyanine5.5 mouse anti-human CD11b (BioLegend, 301328, ICRF44, 1:200 dilution)
4. BV510 mouse anti-human CD27 (BioLegend, 302835, O323, 1:200 dilution)
5. BV711 mouse anti-human CD28 (BioLegend, 302948, CD28.2, 1:100 dilution)
6. PerCP/Cyanine5.5 mouse anti-human CD3 (BioLegend, 344808, SK7, 1:200 dilution
7. APC/Fire 810 mouse anti-human CD38 (BioLegend, 303550, HIT2, 1:100 dilution)
8. BV570 mouse anti-human CD56 (NCAM) (BioLegend, 318330, HCD56, 1:200 dilution)
9. PE mouse anti-human CD62L (BioLegend, 304806, DREG-56, 1:200 dilution)
10. PE/Cyanine7 mouse anti-human CD69 (BioLegend, 310912, FN50, 1:200 dilution)
11. BV650 mouse anti-human CD69 (BioLegend, 310934, FN50, 1:200 dilution)
12. PE/Cyanine5 mouse anti-human CD95 (BioLegend, 305610, DX2, 1:400 dilution)
13. BV605 mouse anti-human CTLA-4 (BioLegend, 369610, BN13, 1:100 dilution)
14. PE/Cyanine7 mouse anti-human CTLA-4 (BioLegend, 349914, L3D10, 1:50 dilution)
15. BV421 mouse anti-human CXCR3 (CD183) (BioLegend, 353716, G025H7, 1:100 dilution)
16. APC mouse anti-human CXCR6 (CD186) (BioLegend, 356006, K041E5, 1:100 dilution)
17. FITC mouse anti-human HLA-DR (BioLegend, 307604, L243, 1:200 dilution)
18. Pacific Blue armenian hamster anti-human ICOS (CD278) (BioLegend, 313522, C398.4A, 1:100 dilution)
19. Alexa Fluor 647 mouse anti-human LAG-3-AF647 (BioLegend, 369304, 11C3C65, 1:200 dilution)
20. PerCP mouse anti-human PD-1 (CD279) (BioLegend, 329938, EH12.2H7, 1:200 dilution)
21. BV421 mouse anti-human PD-1 (CD279) (BioLegend, 329920, EH12.2H7, 1:30 dilution)
22. BV785 mouse anti-human IFNγ (BioLegend, 502542, 4S.B3, 1:80 dilution)
23. Pacific Blue mouse anti-human IFNγ (BioLegend, 502521, 4S.B3, 1:100 dilution)
24. FITC mouse anti-human Granzyme B (BioLegend, 515403, GB11, 1:300 dilution)
25. Alexa Fluor 700 rat anti-human IL-2 (BioLegend, 500320, MQ1-17H12, 1:100 dilution)
26. PE/Cyanine7 mouse anti-human TNF-α (BioLegend, 502929, MAb11, 1:200 dilution)
27. Pacific Blue mouse anti-human Perforin (BioLegend, 353305, B-D48, 1:40 dilution)
28. APC rat anti-mouse IFNγ (BioLegend, 505810, XMG1.2, 1:300 dilution)
29. APC Annexin V (BioLegend, 640920, 1:100 dilution)
30. PerCP/Cyanine5.5 rat anti-mouse CD8a (BioLegend, 100734, 53-6.7, 1:500 dilution)
31. BV510 mouse anti-mouse CD45.2 (BioLegend, 109838, 104, 1:500 dilution)
32. APC rat anti-mouse CD366 (Tim-3) (BioLegend, 119706, RNNT3-23, 1:500 dilution)
33. Anti-mouse CD19-PE (1D3/CD19), (BioLegend, 152408, 1:200)
34. Anti-mouse CD3 purified (145-2C11), (Biolegend, 100302, 1:100)
35. Anti-mouse CD28 purified (37.51) (Biolegend 102102, 1:500)
36. Anti-mouse granzyme B PE (QA16A02) (Biolegend 372204, 1:300)
37.Anti-mouse CD69 PercP (H1.2F3) (Biolegend 104520, 1:300)

From BD Biosciences
1. BUV496 mouse anti-human CCR6 (CD196) (BD Biosciences, 612948, 11A9, 1:100 dilution)
2. BUV563 rat anti-human CCR7 (CD197) (BD Biosciences, 741317, 3D12, 1:50 dilution)
3. BV786 mouse anti-human CD103 (BD Biosciences, 743654, Ber-ACT8, 1:400 dilution)
4. BUV661 mouse anti-human CD11c (BD Biosciences, 612967, B-ly6, 1:200 dilution)
5. APC-R700 mouse anti-human CD127 (BD Biosciences, 565185, HIL-7R-M27, 1:50 dilution)
6. BUV615 mouse anti-human CD3 (BD Biosciences, 751252, SK7, 1:300 dilution)
7. PE-Cy7 mouse anti-human CD31 (BD Biosciences, 563651, WM59, 1:200 dilution)
8. BUV737 mouse anti-human CD45RA (BD Biosciences, 612846, HI100, 1:100 dilution)
9. BB700 mouse anti-human CD45RO (BD Biosciences, 745807, UCHL1, 1:80 dilution)
10. BUV395 mouse anti-human CD49a (BD Biosciences, 742363, SR84, 1:100 dilution)
11. BB515 mouse anti-human CD57 (BD Biosciences, 565285, NK-1, 1:400 dilution)
12. BUV805 mouse anti-human CD8 (BD Biosciences, 612889, SK1, 1:200 dilution)
13. BV750 rat anti-human CXCR5 (CD185) (BD Biosciences, 747111, RF8B2, 1:100 dilution)
14. Alexa Fluor 647 mouse anti-human Lck (pY505) (BD Biosciences, 558577, 4/LCK-Y505, 1:15 dilution)

15. Alexa Fluor 488 mouse anti-human S6 (pS235/pS236) (BD Biosciences, 560434, N7-548, 1:15 dilution)
16. Alexa Fluor 488 mouse anti-human S6 (pS240) (BD Biosciences, 560431, N4-41, 1:15 dilution)
17. PE mouse anti-human Src (pY418) (BD Biosciences, 560094, K98-37, 1:15 dilution)
18. BV421 mouse anti-human Akt (pS473) (BD Biosciences, 562599, M89-61, 1:60 dilution)
19. Alexa Fluor 488 mouse anti-human p38 (pT180/pY182) (BD Biosciences, 612594, 36/p38 (pT180/pY182), 1:15 dilution)
20. PE mouse anti-human Zap70 (pY292) (BD Biosciences, 558510, J34-602, 1:15 dilution)
21. Alexa Fluor 647 mouse anti-human PLCγ1 (pY783) (BD Biosciences, 557883, 27/PLC, 1:15 dilution)
22. Pacific Blue mouse anti-human ERK1/2 (pT202/pY204) (BD Biosciences, 562981, 20A, 1:60 dilution)
23. Mouse anti-human CD28 (NA/LE) (BD Biosciences, 555725, CD28.2, 1:1000 dilution)

From Thermo Fisher Scientific
1. PE-Alexa Fluor 700 mouse anti-human CD25-PE (ThermoFisher, MHCD2524, CD25-3G10, 1:50 dilution)
2. Super Bright 436 mouse anti-human CD5 (ThermoFisher, 62-0059-42, UCHT2, 1:400 dilution)
3. APC-eFluor 780 mouse anti-human HLA-DR (ThermoFisher, 47-9952-42, L243, 1:300 dilution)
4. PerCP-eFluor 710 mouse anti-human TCR γ/δ (ThermoFisher, 46-9959-42, B1.1, 1:80 dilution)
5. PE rat anti-mouse Granzyme B (ThermoFisher, 12-8898-82, NGZB, 1:300 dilution)
6. PE Armenian Hamster anti-mouse CD279 (PD-1) (ThermoFisher, 12-9985-82, J43, 1:500 dilution)
7. FITC rat anti-mouse TNF alpha (ThermoFisher, 11-7321-82, MP6-XT22, 1:300 dilution)

From Cytek Biosciences
1. cFluor YG584 mouse anti-human CD4 (Cytek Biosciences, R7-20041, SK3, 1:400 dilution)

From R&D Systems
1. PE mouse anti-human Glut1 (R&D Systems, FAB1418P, 202915, 1:25 dilution)

From Cell Signalling Technology
1. Rabbit anti-human Lamin B1 (D4Q4Z) (Cell Signaling Technology, 12586S, D4Q4Z1, 1:1000 dilution)
2. Mouse anti-human β-Actin (8H10D10) (Cell Signaling Technology, 3700, 8H10D10, 1:1000 dilution)

From Santa Cruz
1. Mouse anti-human Nuclear Factor of Activated T Cells 5 (NFAT5) (Santa Cruz, sc-398171, 1:500 dilution)

From Enzo Life Sciences
1. Mouse Anti-human CD3 (TR-66) (Enzo life sciences, ALX-804-822-C100, TR-66, 1:1000 dilution)

Validation

Validation statements for all antibodies listed above can be accessed via the respective manufacturer's website links provided below.

From BioLegend
1. https://www.biolegend.com/en-us/products/pe-dazzle-594-anti-human-cd194-ccr4-antibody-10242
2. https://www.biolegend.com/en-us/products/alexa-fluor-647-anti-human-cd107a-lamp-1-antibody-4969
3. https://www.biolegend.com/en-us/products/percp-cyanine5-5-anti-human-cd11b-antibody-7299
4. https://www.biolegend.com/en-us/products/brilliant-violet-510-anti-human-cd27-antibody-8005
5. https://www.biolegend.com/en-us/products/brilliant-violet-711-anti-human-cd28-antibody-13272
6. https://www.biolegend.com/en-us/products/percp-cyanine5-5-anti-human-cd3-antibody-6932
7. https://www.biolegend.com/en-us/products/apcfire-810-anti-human-cd38-antibody-19464
8. https://www.biolegend.com/en-us/products/brilliant-violet-570-anti-human-cd56-ncam-antibody-7370
9. https://www.biolegend.com/en-us/products/pe-anti-human-cd62l-antibody-653
10. https://www.biolegend.com/en-us/products/pe-cyanine7-anti-human-cd69-antibody-1918
11. https://www.biolegend.com/en-us/products/brilliant-violet-650-anti-human-cd69-antibody-8345
12. https://www.biolegend.com/en-us/products/pe-cyanine5-anti-human-cd95-fas-antibody-644
13. https://www.biolegend.com/en-us/products/brilliant-violet-605-anti-human-cd152-ctla-4-antibody-13910
14. https://www.biolegend.com/en-us/products/pe-cyanine7-anti-human-cd152-ctla-4-antibody-9294
15. https://www.biolegend.com/en-us/products/brilliant-violet-421-anti-human-cd183-cxcr3-antibody-7712
16. https://www.biolegend.com/en-us/products/apc-anti-human-cd186-cxcr6-antibody-8622
17. https://www.biolegend.com/en-us/products/fitc-anti-human-hla-dr-antibody-788
18. https://www.biolegend.com/en-us/products/pacific-blue-anti-human-mouse-rat-cd278-icos-antibody-7373
19. https://www.biolegend.com/en-us/products/alexa-fluor-647-anti-human-cd223-lag-3-antibody-12465
20. https://www.biolegend.com/en-us/products/percp-anti-human-cd279-pd-1-antibody-9865
21. https://www.biolegend.com/en-us/products/brilliant-violet-421-anti-human-cd279-pd-1-antibody-7191
22. https://www.biolegend.com/en-us/products/brilliant-violet-785-anti-human-ifn-gamma-antibody-7986
23. https://www.biolegend.com/en-us/products/pacific-blue-anti-human-ifn-gamma-antibody-4147
24. https://www.biolegend.com/en-us/products/fitc-anti-human-mouse-granzyme-b-antibody-6066
25. https://www.biolegend.com/en-us/products/alexa-fluor-700-anti-human-il-2-antibody-3439
26. https://www.biolegend.com/en-us/products/pe-cyanine7-anti-human-tnf-alpha-antibody-6515
27. https://www.biolegend.com/en-us/products/pacific-blue-anti-human-perforin-antibody-7695

From BD Biosciences
1. https://www.bdbiosciences.com/en-de/products/reagents/flow-cytometry-reagents/research-reagents/single-color-antibodies-ruo/buv496-mouse-anti-human-cd196-ccr6.612948

2. https://www.bdbiosciences.com/en-de/products/reagents/flow-cytometry-reagents/research-reagents/single-color-antibodies-ruo/buv563-rat-anti-human-ccr7-cd197.741317
3. https://www.bdbiosciences.com/en-de/products/reagents/flow-cytometry-reagents/research-reagents/single-color-antibodies-ruo/bv786-mouse-anti-human-cd103.743654
4. https://www.bdbiosciences.com/en-de/products/reagents/flow-cytometry-reagents/research-reagents/single-color-antibodies-ruo/buv661-mouse-anti-human-cd11c.612967
5. https://www.bdbiosciences.com/en-de/products/reagents/flow-cytometry-reagents/research-reagents/single-color-antibodies-ruo/apc-r700-mouse-anti-human-cd127.565185
6. https://www.bdbiosciences.com/en-de/products/reagents/flow-cytometry-reagents/research-reagents/single-color-antibodies-ruo/buv615-mouse-anti-human-cd3.751252
7. https://www.bdbiosciences.com/en-de/products/reagents/flow-cytometry-reagents/research-reagents/single-color-antibodies-ruo/pe-cy-7-mouse-anti-human-cd31.563651
8. https://www.bdbiosciences.com/en-de/products/reagents/flow-cytometry-reagents/research-reagents/single-color-antibodies-ruo/buv737-mouse-anti-human-cd45ra.612846
9. https://www.bdbiosciences.com/en-de/products/reagents/flow-cytometry-reagents/research-reagents/single-color-antibodies-ruo/bb700-mouse-anti-human-cd45ro.745807
10. https://www.bdbiosciences.com/en-de/products/reagents/flow-cytometry-reagents/research-reagents/single-color-antibodies-ruo/buv395-mouse-anti-human-cd49a.742363
11. https://www.bdbiosciences.com/en-de/products/reagents/flow-cytometry-reagents/research-reagents/single-color-antibodies-ruo/bb515-mouse-anti-human-cd57.565285
12. https://www.bdbiosciences.com/en-de/products/reagents/flow-cytometry-reagents/research-reagents/single-color-antibodies-ruo/buv805-mouse-anti-human-cd8.612889
13. https://www.bdbiosciences.com/en-de/products/reagents/flow-cytometry-reagents/research-reagents/single-color-antibodies-ruo/bv750-rat-anti-human-cxcr5-cd185.747111
14. https://www.bdbiosciences.com/en-de/products/reagents/flow-cytometry-reagents/research-reagents/single-color-antibodies-ruo/alexa-fluor-647-mouse-anti-lck-py505.558577
15. https://www.bdbiosciences.com/en-de/products/reagents/flow-cytometry-reagents/research-reagents/single-color-antibodies-ruo/alexa-fluor-488-mouse-anti-s6-ps235-ps236.560434
16. This product has been discontinued.
17. https://www.bdbiosciences.com/en-br/products/reagents/flow-cytometry-reagents/research-reagents/single-color-antibodies-ruo/pe-mouse-anti-src-py418.560094
18. https://www.bdbiosciences.com/en-br/products/reagents/flow-cytometry-reagents/research-reagents/single-color-antibodies-ruo/bv421-mouse-anti-akt-ps473.562599
19. https://www.bdbiosciences.com/en-br/products/reagents/flow-cytometry-reagents/research-reagents/single-color-antibodies-ruo/alexa-fluor-488-mouse-anti-p38-mapk-pt180-py182.612594
20. https://www.bdbiosciences.com/en-br/products/reagents/flow-cytometry-reagents/research-reagents/single-color-antibodies-ruo/pe-mouse-anti-zap70-py292.558510
21. https://www.bdbiosciences.com/en-br/products/reagents/flow-cytometry-reagents/research-reagents/single-color-antibodies-ruo/alexa-fluor-647-mouse-anti-human-plc-1-py783.557883
22. https://www.bdbiosciences.com/en-br/products/reagents/flow-cytometry-reagents/research-reagents/single-color-antibodies-ruo/bv421-mouse-anti-erk1-2-pt202-py204.562981
23. https://www.bdbiosciences.com/en-de/products/reagents/flow-cytometry-reagents/research-reagents/single-color-antibodies-ruo/purified-na-le-mouse-anti-human-cd28.555725From Thermo Fisher Scientific
1. https://www.thermofisher.com/antibody/product/CD25-Antibody-clone-CD25-3G10-Monoclonal/MHCD2524
2. https://www.thermofisher.com/antibody/product/CD5-Antibody-clone-UCHT2-Monoclonal/62-0059-42
3. https://www.thermofisher.com/antibody/product/HLA-DR-Antibody-clone-L243-Monoclonal/47-9952-42
4. https://www.thermofisher.com/antibody/product/TCR-gamma-delta-Antibody-clone-B1-1-Monoclonal/46-9959-42

From Cytek Biosciences
1. https://cytekbio.com/products/cfluor-568-anti-human-cd4?variant=32351881986084

From R&D Systems
1. https://www.rndsystems.com/products/human-glut1-pe-conjugated-antibody-202915_fab1418p

From Cell Signalling Technology
1. https://www.cellsignal.com/products/primary-antibodies/lamin-b1-d4q4z-rabbit-mab/12586
2. https://www.cellsignal.com/products/primary-antibodies/b-actin-8h10d10-mouse-mab/3700

From Santa Cruz
1. https://www.scbt.com/de/p/nfat5-antibody-f-9

From Enzo Life Sciences
1. https://www.enzo.com/product/cd3-human-monoclonal-antibody-tr66/

Eukaryotic cell lines

Policy information about cell lines and Sex and Gender in Research

Cell line source(s): A375 cell line, PancO2 and PancO2-EpCAM cell lines were kindly gifted by the Sebastian Kobold lab.

Authentication	A375, STR profiling originally been performed by the provider; PancO2 and PancO2-EpCAM cell lines: authentification performed by the the Eurofins Cell Line Athentification service. PancO2 cells were equipped with mROR1 in-house.
Mycoplasma contamination	Cell lines were regularly tested for mycoplasms contamination
Commonly misidentified lines (See ICLAC register)	*Name any commonly misidentified cell lines used in the study and provide a rationale for their use.*

Palaeontology and Archaeology

Specimen provenance	*Provide provenance information for specimens and describe permits that were obtained for the work (including the name of the issuing authority, the date of issue, and any identifying information). Permits should encompass collection and, where applicable, export.*
Specimen deposition	*Indicate where the specimens have been deposited to permit free access by other researchers.*
Dating methods	*If new dates are provided, describe how they were obtained (e.g. collection, storage, sample pretreatment and measurement), where they were obtained (i.e. lab name), the calibration program and the protocol for quality assurance OR state that no new dates are provided.*

☐ Tick this box to confirm that the raw and calibrated dates are available in the paper or in Supplementary Information.

Ethics oversight	*Identify the organization(s) that approved or provided guidance on the study protocol, OR state that no ethical approval or guidance was required and explain why not.*

Note that full information on the approval of the study protocol must also be provided in the manuscript.

Animals and other research organisms

Policy information about studies involving animals; ARRIVE guidelines recommended for reporting animal research, and Sex and Gender in Research

Laboratory animals	*For laboratory animals, report species, strain and age OR state that the study did not involve laboratory animals.*
Wild animals	*Provide details on animals observed in or captured in the field; report species and age where possible. Describe how animals were caught and transported and what happened to captive animals after the study (if killed, explain why and describe method; if released, say where and when) OR state that the study did not involve wild animals.*
Reporting on sex	*Indicate if findings apply to only one sex; describe whether sex was considered in study design, methods used for assigning sex. Provide data disaggregated for sex where this information has been collected in the source data as appropriate; provide overall numbers in this Reporting Summary. Please state if this information has not been collected. Report sex-based analyses where performed, justify reasons for lack of sex-based analysis.*
Field-collected samples	*For laboratory work with field-collected samples, describe all relevant parameters such as housing, maintenance, temperature, photoperiod and end-of-experiment protocol OR state that the study did not involve samples collected from the field.*
Ethics oversight	*Identify the organization(s) that approved or provided guidance on the study protocol, OR state that no ethical approval or guidance was required and explain why not.*

Note that full information on the approval of the study protocol must also be provided in the manuscript.

Plants

Seed stocks	*Report on the source of all seed stocks or other plant material used. If applicable, state the seed stock centre and catalogue number. If plant specimens were collected from the field, describe the collection location, date and sampling procedures.*
Novel plant genotypes	*Describe the methods by which all novel plant genotypes were produced. This includes those generated by transgenic approaches, gene editing, chemical/radiation-based mutagenesis and hybridization. For transgenic lines, describe the transformation method, the number of independent lines analyzed and the generation upon which experiments were performed. For gene-edited lines, describe the editor used, the endogenous sequence targeted for editing, the targeting guide RNA sequence (if applicable) and how the editor was applied.*
Authentication	*Describe any authentication procedures for each seed stock used or novel genotype generated. Describe any experiments used to assess the effect of a mutation and, where applicable, how potential secondary effects (e.g. second site T-DNA insertions, mosiacism, off-target gene editing) were examined.*

Flow Cytometry

Plots

Confirm that:

☒ The axis labels state the marker and fluorochrome used (e.g. CD4-FITC).

☒ The axis scales are clearly visible. Include numbers along axes only for bottom left plot of group (a 'group' is an analysis of identical markers).

☐ All plots are contour plots with outliers or pseudocolor plots.

☒ A numerical value for number of cells or percentage (with statistics) is provided.

Methodology

Sample preparation	Primary cells were isolated as described in the methods (Ficoll isolation, positive magnetic isolation using microbeads, flow-cytometry assisted cell sorting)
Instrument	BD FACSAria Fusion (BD Biosciences), Cytek Aurora (CYTEK), CytoFLEX (Beckman Coulter), MACSQuant Analyzer (Miltenyi Biotec), BD LSRFortessa (BD Biosciences), BD FACSAriaIII (BD Biosciences), Attune NxT (Thermofisher)
Software	Flowjo Software (version 10.9.0) (Tree Star Inc)
Cell population abundance	For cell sorting, lymphocytes were gated by FSC-A/SSC-A, then singlets were selected by FSC-A/FSC-H and SSC-A/SSC-W, exclusion of dead cells by Hoechst 33258, and further gated for CD4- CD8+ CD45RA-. For protein marker expression analysis, lymphocytes were gated by FSC-A/SSC-A, then singlets were selected by FSC-A/FSC-H, exclusion of dead cells by Zombie dyes or 7-AAD or Hoechst 33258. Protein marker gating based on negative population from unstained control.
Gating strategy	A gating strategy of the memory and naive CD8+ T cells used for functional and transcriptomic analyses has been provided as supplementary figure.

☒ Tick this box to confirm that a figure exemplifying the gating strategy is provided in the Supplementary Information.
